# Development and evaluation of a novel dietary bisphenol A (BPA) exposure risk tool

**DOI:** 10.1186/s40795-022-00634-4

**Published:** 2022-12-06

**Authors:** Jennifer C. Hartle, Roy S. Zawadzki, Joseph Rigdon, Juleen Lam, Christopher D. Gardner

**Affiliations:** 1 Department of Public Health and Recreation, San José State University, San José, CA 95192 USA; 2 Department of Biostatistics, California Polytechnic State University-San Luis Obispo, San Luis Obispo, CA 94307 USA; 3 Department of Statistics, Donald Bren School of Information and Computer Sciences, University of California, Irvine, CA 92697 USA; 4 Department of Biostatistics and Data Science, Wake Forest School of Medicine, Winston-Salem, NC 27157 USA; 5 University of California at San Francisco, San Francisco, CA 94107 USA; 6 California State University East Bay, Hayward, CA 94542 USA; 7 Stanford Prevention Research Center, Stanford University School of Medicine, Stanford, CA 94305 USA

**Keywords:** Bisphenol A, Food packaging, Environmental phenols, Diet, Exposure, Risk, Questionnaire

## Abstract

**Background:**

Exposure to endocrine disrupting chemicals such as bisphenol A (BPA) is primarily from the diet through canned foods. Characterizing dietary exposures can be conducted through biomonitoring and dietary surveys; however, these methods can be time-consuming and challenging to implement.

**Methods:**

We developed a novel dietary exposure risk questionnaire to evaluate BPA exposure and compared these results to 24-hr dietary recall data from participants (*n* = 404) of the Diet Intervention Examining The Factors Interacting with Treatment Success (DIETFITS) study, a dietary clinical trial, to validate questionnaire responses. High BPA exposure foods were identified from the dietary recalls and used to estimate BPA exposure. Linear regression models estimated the association between exposure to BPA and questionnaire responses. A composite risk score was developed to summarize questionnaire responses.

**Results:**

In questionnaire data, 65% of participants ate canned food every week. A composite exposure score validated that the dietary exposure risk questionnaire captured increasing BPA exposure. In the linear regression models, utilizing questionnaire responses vs. 24-hr dietary recall data, participants eating canned foods 1–2 times/week (vs. never) consumed 0.78 more servings (*p* < 0.001) of high BPA exposure foods, and those eating canned foods 3+ times/week (vs. never) consumed 0.89 more servings (*p* = 0.013) of high BPA exposure foods. Participants eating 3+ packaged items/day (vs. never) consumed 62.65 more total grams of high BPA exposure food (*p* = 0.036).

**Conclusions:**

Dietary exposure risk questionnaires may provide an efficient alternative approach to 24-hour dietary recalls to quantify dietary BPA exposure with low participant burden.

**Trial registration:**

The trial was prospectively registered at clinicaltrials.gov as NCT01826591 on April 8, 2013.

**Supplementary Information:**

The online version contains supplementary material available at 10.1186/s40795-022-00634-4.

## Introduction

Bisphenol A (BPA) is a high production volume synthetic chemical with endocrine disrupting properties used in the synthesis of polycarbonate plastic and epoxy resins. Exposure to endocrine disrupting chemicals, which interfere with hormone production, metabolism, and function, are of concern due to potential effects on male and female reproduction, endocrine function, metabolism, obesity, cardiovascular disease, and increased occurrence of breast and prostate cancer [1–5]. Although several studies have determined that residual and unreacted BPA migrates into the foods it contacts [6–10], BPA has been approved for use in food packaging. Due to its widespread use in food packaging and other consumer products, along with the absence of environmental control means for its disposal, BPA is a ubiquitous contaminant of water, air, soil, and dust [11, 12]. As a result, 93% of Americans have measurable concentrations of BPA in their urine [13].

Potential routes of exposure to BPA include inhalation, dermal absorption, and non-dietary ingestion, with its main exposure pathway being dietary ingestion of contaminated food [10–12, 14–20]. Inhalation exposures occur from breathing in contaminated dust [14, 20], dermal absorption occurs from handling thermal receipts [18], and non-dietary ingestion can occur after the application of dental sealants [21]. The main contributor of dietary ingestion exposure to BPA for adults is from consuming canned foods [14, 22]. Other sources of dietary ingestion exposure to BPA includes food or beverages stored and/or heated in polycarbonate plastic containers [23, 24] and from polyvinyl chloride plastic wrap [7].

Characterizing typical chemical exposures of non-persistent chemicals such as BPA can be challenging. BPA is rapidly metabolized in the body and exposure can be measured from conjugated and unconjugated BPA in urine [25]. Urinary measures of BPA reflect recent exposures and exhibit intra-individual variability [26]. As ingestion is the main exposure pathway for BPA, dietary assessments can be utilized to identify BPA exposure sources. Dietary assessment methods include 24-hour dietary recalls, food frequency questionnaires, and dietary logs. Research has shown that 24-hour dietary recalls utilized in dietary exposure assessment are useful to predict urinary biomarkers of BPA exposure [27, 28].

However, collecting biomarkers of exposure and detailed dietary assessments such as 24-hour dietary recalls are time-consuming and expensive processes that must be administered by trained and qualified personnel. It is not always realistic or feasible to utilize urinary BPA exposure measurements or detailed dietary recalls to quantify dietary BPA exposures. In the absence of urinary biomarkers and dietary recalls, the availability of other methods that can help to predict dietary exposures reliably is critical.

One alternative approach to estimate exposure is risk-based questionnaires designed to identify eating patterns that potentially put people at the highest risk of BPA exposure. They are less time intensive, inexpensive, and do not need to be administered by a trained professional. Exposure risk questionnaires have been used to assess dietary exposures to BPA and health outcomes, including BPA exposures and prostate cancer in Hong Kong [29], and BPA exposure and childhood obesity in Samoa [30]. Potential reasons for poor predictive performance could be that the dietary assessment methods are not accurate enough or non-dietary exposures are a greater source of exposure than previously thought.

In this study, we developed a novel dietary exposure risk questionnaire to identify the consumption of foods likely to be contaminated with BPA. We then evaluated our questionnaire by comparing its associations with BPA food consumption patterns identified in 24-hour dietary recalls. Our goal was to assess the correlation between the amount of BPA consumption reported in our questionnaire and the amount of BPA consumption reported in the 24-hour dietary recall to evaluate the questionnaire’s viability as an alternative method to detailed dietary assessments to determine exposure levels.

## Methods

### Study population

Our research utilized data collected during the Diet Intervention Examining The Factors Interacting with Treatment Success (DIETFITS) study, a year-long dietary clinical trial conducted in the San Francisco Bay Area between May 2014–May 2016 [31]. Participants were recruited from the general population and were healthy women and men, 18–50 years of age, with a BMI between 28 and 40 kg/m^2^. Further information on the DIETFITS study and recruitment has been previously published [31]. Our research includes participants of DIETFITS, starting with the trial’s second through its fifth and final cohort.

### Questionnaire development

The BPA dietary exposure risk questionnaire was developed and piloted in April 2014 based upon literature describing which food sources or food preparation methods are most likely to increase exposure to BPA and methods developed to score this type of exposure [29, 32]. The more packaged and processed foods are, the more BPA a food can contain. Specifically, the BPA dietary exposure risk questionnaire (Supplemental Material- Questionnaire, See Additional file 1) asked about the frequency that participants undertake certain dietary behaviors that increase the risk of BPA exposure: eating canned food [27, 33], microwaving food in plastic food storage containers [23], drinking beverages in polycarbonate plastic containers [34], drinking hot beverages from polycarbonate plastic containers [6], microwaving food covered with polyvinyl chloride-based plastic stretch wrap [7], eating microwaveable meals [35, 36], and eating packaged food [36, 37]. Questions were scored using frequency per day or week and Likert-scale style coding.

A composite BPA exposure (BPAe) risk score of the seven questions on the dietary exposure risk questionnaire was created to enable bivariate comparisons between the questionnaire and BPA intake reported in dietary recalls. BPAe was created by (i) recoding questionnaire responses such that lower responses indicated lower frequency and higher responses indicated a higher frequency of conducting BPA exposing activities (e.g., 1 = Never and 5 = Most), (ii) choosing the minimum number of principal components (PCs) that explained at least 90% of the variation in the data, and (iii) calculating the quintile of the sum of the PCs weighted by the percentage of variation explained by each PC. For example, if 3 PCs explained 90% of the variation, e.g., PC1 75%, PC2 10%, and PC3 5%, and the values of PC1, PC2, and PC3, were 1, 1, and 1, respectively, BPAe would be the quintile containing 1*(0.75) + 1*(0.15) + 1*(0.05) = 0.9. Thus, BPAe was equal to 1 for the lowest exposure and 5 for the highest exposure. To validate scores, each of the seven questions was compared to levels of BPAe to ensure increasing values of question responses across the BPAe categories.

### Quantifying dietary BPA intake in dietary recalls

The DIETFITS study assessed dietary behaviors using 24-hour dietary recalls. The recalls were collected using the Nutrition Data System for Research (NDSR) software versions 2012–2015, developed by the Nutrition Coordinating Center (NCC), University of Minnesota, Minneapolis, MN. Three dietary recalls were administered at each data collection time point, aiming for two weekdays and one weekend day, to estimate typical eating patterns. Dietary data collected with NDSR was coded upon collection into a unique food identification code (Food ID) with accompanying Food Description.

The DIETFITS study questionnaire and the dietary recalls were administered over the same two-week window at baseline, 3, 6, and 12 months. Our BPA dietary exposure risk questionnaire was a subset of questions within the main study questionnaire. In our analyses, we considered only data collected at baseline as participants were explicitly instructed over the course of the study to favor whole foods rather than processed foods, and thus BPA exposure was anticipated to be highest at baseline. The average of the baseline dietary recalls was utilized for our analysis.

We identified foods likely to be high in BPA contamination from the dietary recall data. We searched the NDSR Foods 2017 database, containing information for 32,300 food items, for the keywords “canned”, “packaged”, and “microwave” in the NCC Food Descriptions to develop a subset of food items possibly containing BPA. There were 317 items listed in NDSR as having “canned” in their Food Description. There were 866 items that had the word “packaged” in their Food Description. All but two of these packaged items were described as a type of sweet, including snack cakes, ice cream, or frozen treats. Twelve items had the word “microwave” in their Food Description. These were all packaged foods, either frozen meals, microwave-in-a-cup meals, or microwave popcorn. From this method, we created a list of canned foods, packaged food, and microwave foods (Supplemental Tables S1-S3, See Additional file 1).

To estimate BPA exposure from the dietary recalls, we first recorded the number of specific food items consumed per participant from the lists of canned, packaged, and microwave specific BPA foods created from the NDSR Food Descriptions. Next, we created two variables to describe an estimate of BPA exposure: (i) weighted servings and (ii) total grams. Weighted servings were calculated as the sum of the total number of servings of foods consumed from the canned, packaged, and microwave lists, with each item weighted by the potential concentration of BPA contamination: 1 x canned food items, 0.25 x packaged food, and 0.25 x microwave food, a weighting scale developed and applied in previous research [32], reflecting the higher BPA contamination potential of consuming canned foods. Our method differed from the original research as we applied the weighing scale to the dietary recall. Total grams were calculated as the sum of grams consumed from all canned, packaged, and microwave food items identified on the lists. Consequently, each study participant had up to eight dietary BPA outcomes: weighted servings and grams of canned, packaged, microwave, and overall BPA.

### Statistical methods

Study demographics were summarized using means and standard deviations for continuous variables and sample sizes and percentages for categorical variables. The BPAe composite scores were used as a first approach to measuring the association between the dietary exposure risk questionnaire and overall BPA in dietary recall data. Kruskal-Wallis rank-sum tests (K-W) were used to test the null hypothesis that weighted servings and total grams were equal across the five groups of BPAe. Heavily right-skewed outcome data indicated that K-W was more appropriate than one-way ANOVA [38]. If an overall K-W test was significant, a follow-up analysis was conducted using Dunn’s test pairwise comparisons with a Bonferroni adjustment for multiple hypothesis testing.

A second approach to measure the association between the dietary exposure risk questionnaire and dietary recall data involved the use of linear regression models for overall weighted servings and total grams as a function of the seven separate questions on the questionnaire, maintaining each question as a categorical variable. Questionnaire responses were binned into three levels per question to enable easy interpretation of the findings without making an ordinality assumption. For example, the original question for the number of canned foods eaten per week had answer choices none, 1, 2, 3, 4, 5, and > 5, and we binned to none, 1 or 2, and 3 or more per week. Missing data in the questionnaire data were statistically addressed using multiple imputations via chained equations (MICE) to generate 5 imputed data sets, and model estimates and inferences were combined using Rubin’s rules [39]. Furthermore, questions in which the individual participant marked their answer as “I don’t know” were coded as missing and included in the imputation. Pooled adjusted R^2^-squared and 95% confidence intervals were recorded for each model. In addition, the overall F-statistic was computed using an approximation based on chi-squared statistics [40, 41].

Exploratory analyses were used to measure the association between questionnaire responses to the canned, packaged, and microwave food questions and weighted servings and total grams of analogous food consumption reported in the dietary recalls. All statistical analyses were carried out using R version 3.6.1 [42].

## Results

Our analysis included *n* = 404 participants. We excluded five of the 409 participants in DIETFITS cohorts two through five from analysis because they did not respond to the dietary exposure risk questionnaire. The majority of our study population were female (57.4%), white (54.5%), and had college or post-graduate degrees (75.4%) (Table 1). The average age of participants was 39.1 years old. We had significant missing data in our questions “Do you drink hot beverages from a hard, clear plastic cup?” (26.2% missing) and “How often do you microwave your food with a plastic stretch wrap on?” (19.8% missing). However, the majority of other questions reported < 1% missing data (Table 2).

**Table 1:** Participant Baseline demographics. N (%) for categorical variables and mean (±standard deviation) for continuous variables

	Total
	*N* = 404
**Sex**	
Female	232 (57.4%)
Male	172 (42.6%)
**Age**	39.1 (±6.6)
**Education**	
Some grade school	3 (0.7%)
Some high school	1 (0.2%)
High school graduate	8 (2.0%)
Some college	86 (21.3%)
College graduate	137 (33.9%)
Some post-graduate school	22 (5.4%)
Post-graduate degree	146 (36.1%)
Missing	1 (0.2%)
**Race/ethnicity**	
White	220 (54.5%)
Hispanic	94 (23.3%)
Asian	39 (9.7%)
African American	21 (5.2%)
American Indian/Alaska Native/Pacific Islander	3 (0.7%)
Other	27 (6.7%)
**BMI (kg/m**^**2**^**)**	33.3 (±3.4)

**Table 2:** Dietary Exposure Risk Questionnaire- Questions and Results

Exposure source	Question	Response	*n* (total study participants *N* = 404)
Canned food	In a typical week, how often do you eat canned food?	None: 35.0%^a^	141
		1 or 2 /week: 54.1%	218
		3 or more/week: 10.9% Missing: 0.25%	44 1
Microwave in plastic	How often do you microwave food stored in plastic containers?	Never: 23.0%	87
		Infrequently or Sometimes: 56.1%	212
		Often or Always: 20.9% Missing: 6.4%	79 26
Polycarbonate plastic water bottles	How often do you drink beverages from a re-usable, hard plastic bottle?	Don’t know or Never: 13.4%	54
		Infrequently or Sometimes: 42.0%	169
		Often or Always: 44.5% Missing: 0.5%	179 2
Hot drinks in polycarbonate plastic cups	Do you drink hot beverages from a hard, clear plastic cup?	Don’t know or Never: 29.2%	87
		Infrequently or Sometimes: 53.7%	160
		Often or Always: 17.1% Missing: 26.2%	51 106
Microwave with stretch wrap	How often do you microwave your food with plastic stretch wrap on?	Don’t know or Never: 57.7%	187
		Infrequently or Sometimes: 38.0%	123
		Often or Always: 4.3% Missing: 19.8%	14 80
Microwave meals	In a typical day, how many prepared, microwavable meals do you eat?	Don’t know or None: 14.2%	57
		1 or 2 items/day: 17%	68
		3 or more items/day: 68.8% Missing: 0.74%	276 3
Packaged food	In a typical day, how many packaged food items do you eat?	None or 1 item/day: 55.9%	224
		2 or 3 items/day: 37.4%	150
		4 or more items/day: 6.7% Missing: 0.74%	27 3

^a^Percentages calculated include only the number of participants that responded to the particular question. Participants that did not answer the particular question are excluded from the denominator

Our dietary exposure risk questionnaire revealed dietary activities associated with a range of variable BPA exposures. The questionnaire results indicated that 65% of respondents ate canned food on a weekly basis, with 11% consuming three or more canned foods per week. Approximately 21% of respondents often or always microwaved their food in plastic storage container. Another potential source of BPA contamination is drinking hot beverages from plastic cups, a habit that approximately 71% of our respondents reported. Food was microwaved with a plastic stretch wrap on it by approximately 42% of our respondents. When asked about their consumption of package foods per day, 44% of our respondents stated that they ate two or more packaged foods per day (Table 2).

**Fig. 1:**
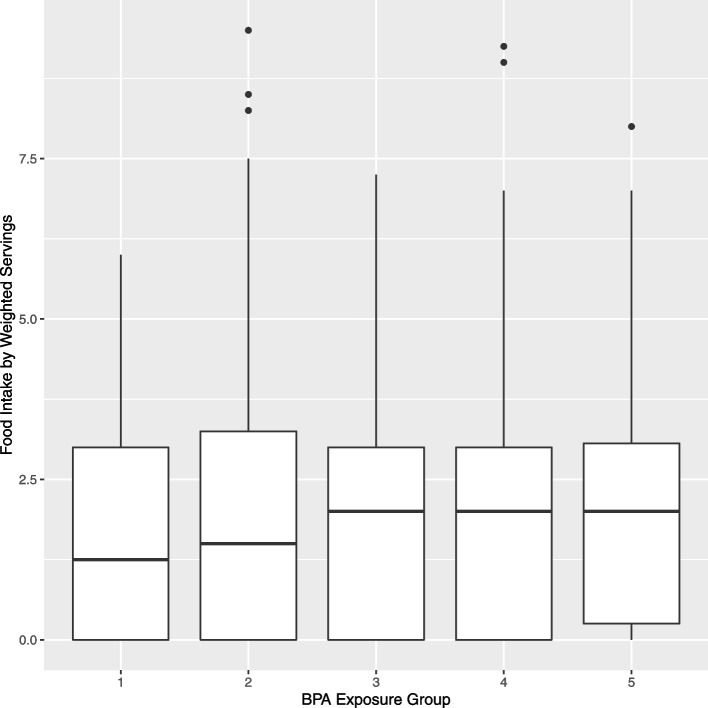
Overall servings by level of BPAe at baseline (Kruskal-Wallis p-value 0.88)

**Fig. 2:**
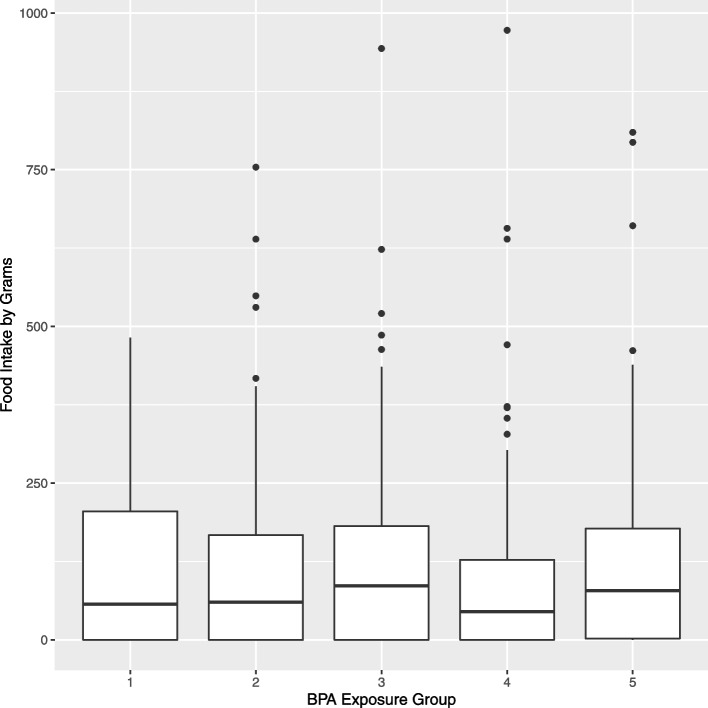
Overall grams by level of BPAe at baseline (Kruskal-Wallis *p*-value 0.77)

Average dietary exposure risk questionnaire responses are shown by level of BPAe in Supplemental Table S4 (See Additional file 1). With the exception of the questionnaire question asking about drinking beverages from re-usable, hard plastic bottles, question responses increased monotonically with increasing level of BPAe, indicating that BPAe captured increasing BPA exposure. Notably, average responses in the low vs. high group of BPAe were 1.8 vs. 3.4 for packaged food-based BPA, 1.4 vs. 3.6 for microwaving in plastic containers, 1.1 vs. 2.6 for microwaving with stretch cling wrap, and 3.0 vs. 4.1 for microwaveable prepared meals. Figures 1 and 2 suggest that the medians of the two measures of exposure increase as the BPAe increases. Kruskal-Wallis tests indicated no significant differences between BPAe risk groups for weighted servings (*p* = 0.88) vs.grams (*p* = 0.77).

From the dietary recalls, median (IQR) weighted servings were equal to 2 (3) and total grams 98.3 (138.7). Linear regression models showed differences in estimated exposure to BPA by levels of questionnaire responses (Table 3). In the weighted servings linear regression model, the average weighted servings of those who were in the canned foods “medium” and “high” exposure groups were significantly higher than those in the “low” exposure group at 0.78 servings (*p <* 0.001) and 0.89 servings (*p* = 0.013), respectively. No significant difference was found between the exposure groups in the linear regression grams model for canned food.

**Table 3:** Linear regression model comparing Dietary Exposure Risk Questionnaire and 24-hr Dietary Recall Data with imputed data

	Model 1: Weighted servings^**a**^		Model 2: Overall grams of BPA Containing Food	
Dietary exposure risk questionnaire responses	Estimate (SE)	*P*-value	Estimate (SE)	*P*-value
How often do you eat canned food? (ref = None)				
1–2 times/week	**0.78 (0.22)**^b^	**<.001**	30.42 (17.53)	0.084
3–5+ times/week	**0.89 (0.36)**	**0.013**	21.69 (28.69)	0.446
How often do you microwave food stored in plastic containers (ref = Never)				
Infrequently/sometimes	−0.23 (0.27)	.403	−11.06 (22.33)	0.621
Often/always	−0.05 (0.35)	.890	−33.02 (27.11)	0.224
How often do you drink beverages from a re-usable, hard plastic bottle? (ref = Don’t know/never)				
Infrequently/sometimes	−0.02 (0.35)	0.957	2.13 (27.48)	0.938
Often/always	−0.09 (0.34)	0.784	2.76 (27.15)	0.919
Do you drink hot beverages from a hard, clear plastic cup? (ref = Don’t know/never)				
Infrequently/sometimes	−0.17 (0.30)	0.565	1.45 (21.91)	0.947
Often/always	0.15 (0.36)	0.670	30.96 (28.92)	0.291
How often do you microwave your food with plastic stretch wrap on? (ref = Don’t know/never)				
Infrequently/sometimes	−0.03 (0.24)	0.904	−15.31 (20.09)	0.449
Often/always	−0.71 (0.62)	0.263	−29.37 (48.36)	0.546
In a typical day, how many prepared, microwavable meals do you eat? (ref = Don’t know/none)				
1–2 items/day	0.18 (0.38)	0.641	22.31 (30.04)	0.458
3+ items/day	0.16 (0.30)	0.594	20.51 (23.94)	0.392
In a typical day, how many packaged food items do you eat? (ref = None)				
1–2 items/day	−0.21 (0.31)	0.495	12.65 (24.32)	0.603
3–5+ items/day	0.34 (0.37)	0.359	**62.65 (29.81)**	**0.036**

^a^Weighted servings calculated with: # of servings x multiplier according to the type of food. Canned food X 1.0, Microwaved food × 0.25, packaged food X 0.25

^b^Bolded values are significant. *P* < 0.05

Weighted servings model: F (17, 39.21) = 1.378, *p* = 0.199, R^2^ 3.2%

Overall grams model: F (17, 36.25) = 0.882, *p* = 0.598, R^2^ 1.7%

For the grams model, the individuals in the “high” exposure group in eating packaged foods had significantly higher exposure to our target BPA foods compared to the “low” exposure group with a difference of 62.65 g (*p* = 0.036). No significant differences in exposure were found for the remaining grams models.

**Fig. 3:**
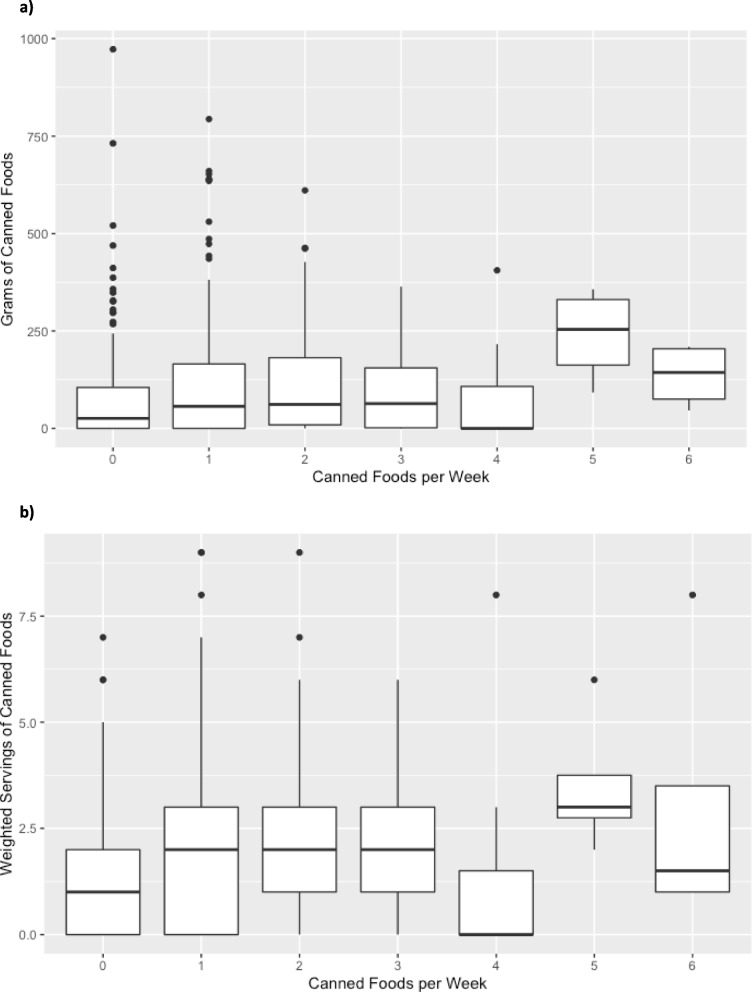
Analysis of Canned Food Consumption- Recall vs. Questionnaire. **a** Grams of canned food identified from dietary recall vs. number of canned foods consumed per week from exposure questionnaire (overall Kruskal-Wallis *p*-value 0.77). **b** Estimated weighted servings of canned foods from the dietary recall compared to the number of canned foods consumed per week from exposure questionnaire (Overall Kruskal-Wallis p-value 0.006; category 0 vs. 1 *p* = 0.03, category 0 vs. 2 *p* = 0.03)

Follow-up analysis between questionnaire responses to the canned food question yielded statistically significant Kruskal-Wallis tests for servings (*p* = 0.006) but not for grams (*p* = 0.772). For both measures, however, Fig. 3a and b show that the two measures of exposure generally increases as frequency of eating canned food increases. Follow-up pairwise comparisons approach a significant increase in exposure between those who responded that they eat canned foods once per week and those who eat canned foods twice per week (*p* = 0.0264 and *p* = 0.0256, respectively, with *p* = 0.025 considered significant due to the Bonferroni correction). An insufficient number of participants reporting in some response categories prevented us from performing this analysis on packaged and microwaved foods.

## Discussion

To determine if our dietary exposure risk questionnaire could potentially be used as a method to differentiate levels of BPA exposure, we examined associations between individual questions and the composite score of a novel questionnaire and 24-hour dietary recall data. Our data found significant associations for select responses to questions about canned food and packaged food. Detecting a significant association between eating canned food in our dietary exposure risk questionnaire and eating canned food in the 24-hour dietary recall reinforces past research that found canned food consumption can be more readily identified from dietary recall food descriptions than other dietary behaviors with BPA exposure potential [27].

We found many food items with high BPA exposure potential missing from Food Description groupings and some helpful groupings missing altogether. To identify foods in the general “packaged” group, the NDSR NCC Food Descriptions list 866 packaged foods, the most out of our three packaging categories. A shortcoming of the “packaged” food group is that almost all packaged foods identified are desserts, 864 of the 866. This misses the identification of many other non-canned packaged foods that are regularly consumed, including grain products such as cereal, crackers, and granola bars. In addition, NDSR food descriptions identify very few microwave foods, only 12, notably missing all frozen pre-prepared microwave meals. A further limitation is that food descriptions do not differentiate between types of food packaging materials (e.g. polyethylene plastic) that have the varying potential for food contamination.

Eating patterns identified in food frequency questionnaires may not always agree with food records documenting daily habits. In a similar study, Nomura, et al. [32] compared food frequency questionnaire data that asked about eating habits over the past 12 months and 24-hour food records (similar to our 24-hour dietary recall, used NDSR for coding, except it was self-recorded). In their comparison of questionnaire data and food records, no associations were found, even for canned food. As only recent BPA exposures are measured in urinary samples, using methods that assess short term eating habits might be better suited to predicting urinary BPA concentrations. As evidenced by the same study by Nomura et al. [32], an association was found between urinary BPA concentration and the 24-hour food record’s total BPA score and canned food consumption; whereas, no associations were found between urinary BPA concentration and the food frequency questionnaire. Building on this knowledge, our questionnaire aimed to measure more recent dietary patterns, with modest success in finding an association between our questionnaire and 24-hour dietary recalls.

Our dietary exposure risk questionnaire asks questions about general eating patterns instead of recording specific types and brands of food, and thus there is no administrative burden to update the food lists with the latest packaged foods available in the supermarket as is required with dietary software. This also eliminates the issue of needing to find a specific food ID or food code to match up with the description of what a participant has eaten. Another common challenge with dietary recalls from a multi-year longitudinal study or pooled dataset drawing from different time points is that as the food lists evolve with a rapidly changing food landscape, new food codes will be added as new foods appear in the marketplace and others will be deactivated as the foods are removed from the marketplace. This makes it difficult to compare data collected from different time points.

In addition to developing a dietary exposure risk questionnaire, we developed a method of identifying and quantifying environmental exposures from food packaging using dietary recall data and their food codes. Using the food descriptions, we identified the types of foods most likely to contain BPA or other chemicals that are used in food packaging using the food description. This is a method that may be useful for a range of chemical exposures, as BPA may not be the only chemical of interest for food contamination. One example are BPA’s chemical analogues; as BPA’s potential for adverse health effects has become known to the public, it has been removed from some food packaging and often times replaced with other bisphenols, such as bisphenol S and bisphenol F [43]. Another compound commonly found in packaged and microwave foods are per- and polyfluoroalkyl substances (PFAS) [44].

An ongoing challenge of assessing dietary exposures from food packaging is that the data collection protocols for 24-hour dietary assessments do not fully document potential sources of food contamination. It is not standard practice for a 24-hour dietary recall to ask what type of packaging that the food came in, what type of vessel was the food heated in, or what type of dishware and utensils were the foods eaten with, all possible sources of BPA exposure. Using the NDSR software, there are limited food IDs that identify the type of packaging. As a consequence of this gap in food packaging identification, it is challenging to align questions about packaging in this study’s dietary exposure risk questionnaire to the 24-hour dietary recall data. During the dietary assessment process, it would be helpful for environmental health research if participants are asked more detailed questions about food packaging and methods of food preparation and storage.

A strength of our research is that the dietary exposure risk questionnaire we developed is brief and easy to understand by people who are seeking an estimate of their typical dietary exposure to BPA. In addition, the questionnaire does not need to be administered in the context of a clinical trial or by a trained professional. The participant burden is low, taking less than 5 mins to complete, and allows for greater flexibility in participation with an online questionnaire format.

An additional strength of our study is that it is a secondary analysis of a clinical trial whose participants were an almost even balance of female and male (57.4 and 42.6%, respectively), and racially/ethnically diverse. This participant makeup reflects the ethnic diversity of the San Francisco Bay Area. The DIETFITS study had more non-Hispanic White and less Asian participants than its geographic area, but otherwise closely resembled the large (over 7 million people in the San Francisco Bay Area) and diverse population it recruited from [45]. We believe that this diversity of gender, race and ethnicity in our study population allows our study to be generalizable to many other populations, predominantly in major metropolitan cities across the United States.

Our study has notable limitations, both in study design and analysis. One limitation was that we did not have urinary measures of BPA to use as a reliable measure of exposure to validate our derived metrics. Second, there is a weakness in comparing the exposure data derived from the questionnaire to the exposure data derived from the 24-hour dietary recall. The dietary exposure risk questionnaire asks about dietary behaviors pertaining to BPA exposures from canned food, polycarbonate plastic, food temperatures, cling wrap use, microwaving, and packaged foods. Comparison to scores from the 24-hour dietary recalls, which only identify canned, packaged, and microwave foods, may be inadequate. Statistical limitations included potential low power to detect differences between the levels of BPA exposure as measured by the individual questions. For example, BPA exposure in ascending answer choices trended upwards but was not statistically significant (see regression coefficients Table 3). In addition, each exposure level had a different number of participants, decreasing the power of each stratum. This suggests that there may be a trend as hypothesized, but due to lack of sample size and unequal distribution among question responses, the magnitude of the coefficients did not reach statistical significance. Secondary analyses using BPAe scores, composite scores, have higher power at the price of lack of granularity in measuring specific associations.

As noted earlier, a strength of our study is that our research population is expected to be generalizable to major metropolitan cities in terms of diverse racial/ethnic representation. However, a concurrent limitation is that generalizability may be limited to other geographic regions. The majority of our participants were highly educated, and all of our participants were from the San Francisco Bay Area, a geographic area with access to fresh fruits and vegetables year-round. Populations from the western parts of the US spend less money on canned vegetables than other regions [46]. Canned fruit spending by western residents is almost as much as the Midwesterners, who spend most of the US regions [46]. Our study population was highly educated, with 75.5% being college graduates or higher. A high level of educational attainment is associated with lower exposure to BPA [47]. Higher educational attainment is also associated with greater diet and health knowledge [48].

## Conclusions

This research developed and tested a novel dietary exposure risk questionnaire aimed at identifying bisphenol A (BPA) exposures from food, with the goal of finding an efficient alternative method to traditional 24-hour dietary recalls. The BPAe composite scores validated that the dietary exposure risk survey questions captured increasing BPA exposure. Our linear regression models confirmed that participants who reported eating more canned food in the risk questionnaire also reported eating more high BPA exposure food in the 24-hour dietary recall. The dietary exposure risk questionnaire could also identify packaged food consumption as an indicator of eating more high BPA exposure food according to the 24-hour dietary recall. We recommend that future methods research developing dietary exposure risk questionnaire compare survey exposure data to BPA exposures measured in biological matrices.

Future research could build on these findings to improve BPA exposure assessment. For example, exposure scores derived from dietary exposure risk questionnaires could be empirically calibrated to accompanying urinary data, offering a close proxy of BPA exposure. Secondly, future research could supplement the 24-dietary recall data with questions about food packaging specifics and food preparation methods to be more closely compared to the dietary exposure risk questionnaire data. Furthermore, a larger sample size could address statistical power issues due to low numbers of respondents at each answer choice. In conclusion, our dietary exposure risk questionnaire has the potential to estimate BPA and other food packaging exposures as a rapid and cost-effective method.

## Supplementary Information

**Additional file 1. **Dietary Exposure Risk Questionnaire- This is the food packaging questionnaire that was embedded into the DIETFITS study questionnaire. **Table S1.** Canned Foods- This table is a listing of all canned foods, as described by their “Food Description” in the NDSR Foods 2017 database. **Table S2.** Packaged Foods- This table is a listing of all packaged foods, as described by their “Food Description” in the NDSR Foods 2017 database. **Table S3.** Microwave Foods- This table is a listing of all microwave foods, as described by their “Food Description” in the NDSR Foods 2017 database. **Table S4.** This table presents the validation of the BPAe (Composite BPA Exposure Score).

## Data Availability

The data generated as part of the dietary exposure risk survey are not publicly available, but are available from the corresponding author upon reasonable request. The data about the participant demographics are not publicly available, but may be available from the principal investigator of the DIETFITS study upon reasonable request.

## Abbreviations

BPA: Bisphenol A
BPAe: Bisphenol A exposure risk composite score
DIETFITS: the Diet Intervention Examining The Factors Interacting with Treatment Success study
Food ID: Food identification code
K-W: Kruskal-Wallis statistical test
NCC: Nutrition Coordinating Center
NDSR: Nutrition Data System for Research
PC: Principal Components
