# Supplementary material for: Development and evaluation of a novel dietary bisphenol A (BPA) exposure risk tool
Source: BMC Nutr. 2022 Dec 6;8:143. doi: 10.1186/s40795-022-00634-4 (PMC9724381; doi:10.1186/s40795-022-00634-4)
Supplement: Supplementary file 1 — Additional file 1. Dietary Exposure Risk Questionnaire- This is the food packaging questionnaire that was embedded into the DIETFITS study questionnaire. Table S1. Canned Foods- This table is a listing of all canned foods, as described by their “Food Description” in the NDSR Foods 2017 database. Table S2. Packaged Foods- This table is a listing of all packaged foods, as described by their “Food Description” in the NDSR Foods 2017 database. Table S3. Microwave Foods- This table is a listing of all microwave foods, as described by their “Food Description” in the NDSR Foods 2017 database. Table S4. This table presents the validation of the BPAe (Composite BPA Exposure Score). [file 40795_2022_634_MOESM1_ESM.docx]

# **Dietary Exposure Risk Questionnaire**

*[All sub-part a’s were used with skip-logic]*

1. In a typical week, how often do you eat canned foods per week? (Never, 1 time/week, 2 times/week, 3 times/week, 4 times/ week, 5 times/week, > 5 times/week)
   1. [if they answer anything greater than 0, survey asks them: Are the cans labeled BPA-free? (Always, Often, Sometimes, Infrequently, Never, I don’t know)
2. How often do you store food in plastic containers? (e.g. Tupperware) (Always, Often, Sometimes, Infrequently, I don’t know)
   1. [If 2 is affirmative] Do you microwave your food in plastic containers? (Yes/No)
3. In a typical week, how often do you drink canned beverages? (Never, 1 time/week, 2 times/week, 3 times/week, 4 times/week. 5 times/week, >5 times/week)
4. How often do you drink beverages from a re-usable, hard plastic bottle? (Always, Often, Sometimes, Infrequently, Never)
   1. [If 4 is affirmative] Are the bottles labeled BPA-free? (Always, Often, Sometimes, Infrequently, Never, I don’t know)
5. How often do you drink beverages from a hard, clear plastic cup? (Always, Often, Sometimes, Infrequently, Never, I don’t know)
   1. [If 5 is affirmative] How often is the beverage hot? (Always, Often, Sometimes, Infrequently, Never, I don’t know)
6. How often do you cover food with plastic stretch wrap? (e.g. Saran Wrap) (Always, Often, Sometimes, Infrequently, Never, I don’t know)
   1. [If 6 is affirmative] How often do you microwave this food with the plastic stretch wrap on? (Always, Often, Sometimes, Infrequently, Never, I don’t know)
7. In a typical day, how many prepared, microwavable meals do you eat? (None or <1 meal/day, 1 meal /day, 2 meals/day, 3 meals/day, >3 meals/day)
8. In a typical day, how many packaged food items do you eat? (e.g. granola bars, cereal, crackers) (None, 1 item/day, 2 items/day, 3 items /day, 4 items /day, 5 items /day, >5 items /day)

# **Supplementary Table S1: Canned Food**

| **Food Description** | **NCC Database Food Group Description** | **USDA Food Group Description** |
| --- | --- | --- |
| gravy, au jus, canned | Gravy and sauces | Gravies from meat, poultry, fish base |
| gravy, beef, canned, regular | Gravy and sauces | Gravies from meat, poultry, fish base |
| gravy, mushroom, prepared with canned mushroom soup | Gravy and sauces | Gravies from meat, poultry, fish base |
| gravy, mushroom, canned | Gravy and sauces | Gravies from meat, poultry, fish base |
| sweeteners, syrup, white sugar or canned fruit syrup | Sugar, syrup, preserves and jelly | Syrups, honey, molasses, sweet toppings |
| vegetables, asparagus, canned - drained, low sodium | Cooked vegetables, fresh, frozen or canned | Other vegetables, cooked |
| vegetables, bamboo shoots - canned and drained | Cooked vegetables, fresh, frozen or canned | Other vegetables, cooked |
| vegetables, beans, refried beans, canned, regular | Vegetable recipes | Dried bean mixtures |
| vegetables, beans, garbanzo beans, canned - drained, regular | Mature dried beans and peas | Dried peas, lentils, and mixtures |
| vegetables, beans, green or string beans, canned - drained, low sodium | Cooked vegetables, fresh, frozen or canned | Other vegetables, cooked |
| vegetables, beans, kidney beans, canned - drained, regular | Mature dried beans and peas | Dried beans |
| vegetables, beans, lima beans, canned - drained, low sodium | Mature dried beans and peas | Other vegetables, cooked |
| vegetables, beans, navy beans, canned - drained, regular | Mature dried beans and peas | Dried beans |
| vegetables, beans, great northern, canned - drained, regular | Mature dried beans and peas | Dried beans |
| vegetables, beans, pinto beans, canned - drained, regular | Mature dried beans and peas | Dried beans |
| vegetables, beans, wax or yellow beans, canned - drained, low sodium | Cooked vegetables, fresh, frozen or canned | Other vegetables, cooked |
| vegetables, beets, canned - drained, low sodium | Cooked vegetables, fresh, frozen or canned | Other vegetables, cooked |
| vegetables, carrots, canned - drained, low sodium | Cooked vegetables, fresh, frozen or canned | Carrots |
| vegetables, corn, yellow, canned, low sodium - drained | Cooked vegetables, fresh, frozen or canned | Other vegetables, cooked |
| vegetables, corn, yellow, canned, regular - drained | Cooked vegetables, fresh, frozen or canned | Other vegetables, cooked |
| vegetables, mushrooms, canned - drained, low sodium | Cooked vegetables, fresh, frozen or canned | Other vegetables, cooked |
| vegetables, onion, fried onion rings, canned | Vegetable recipes | Other cooked vegetables, cooked with sauces, batters, casseroles |
| vegetables, peas, cowpeas, canned - drained | Mature dried beans and peas | Other vegetables, cooked |
| vegetables, peas, green peas, canned - drained, low sodium | Cooked vegetables, fresh, frozen or canned | Other vegetables, cooked |
| vegetables, peppers, hot chili, green, canned - drained | Cooked vegetables, fresh, frozen or canned | Other vegetables, cooked |
| vegetables, potato, canned - drained, low sodium | Cooked vegetables, fresh, frozen or canned | White potatoes, baked and boiled |
| vegetables, pumpkin, canned | Cooked vegetables, fresh, frozen or canned | Pumpkin |
| vegetables, spinach, canned - drained, low sodium | Cooked vegetables, fresh, frozen or canned | Dark-green leafy vegetables |
| vegetables, sprouts, mung bean, canned - drained | Cooked vegetables, fresh, frozen or canned | Other vegetables, cooked |
| vegetables, squash, zucchini, Italian style - canned | Cooked vegetables, fresh, frozen or canned | Other vegetables, cooked |
| vegetables, sweet potato, unknown if baked, boiled, frozen, or canned | Cooked vegetables, fresh, frozen or canned | Sweet potatoes |
| vegetables, tomato, canned, low sodium | Cooked vegetables, fresh, frozen or canned | Tomatoes, cooked |
| vegetables, waterchestnuts, canned | Cooked vegetables, fresh, frozen or canned | Other vegetables, cooked |
| fruit, apple, applesauce, canned, sweetened | Fruits, sweetened | Fruits, excluding berries |
| fruit, apple, applesauce, canned, unsweetened | Fruits, fresh and unsweetened | Fruits, excluding berries |
| fruit, apple, canned, sweetened (drained) | Fruits, sweetened | Fruits, excluding berries |
| fruit, apricot, canned, water pack | Fruits, fresh and unsweetened | Fruits, excluding berries |
| fruit, apricot, canned, juice pack | Fruits, fresh and unsweetened | Fruits, excluding berries |
| fruit, apricot, canned, syrup pack, light | Fruits, sweetened | Fruits, excluding berries |
| fruit, apricot, canned, syrup pack, heavy, not drained | Fruits, sweetened | Fruits, excluding berries |
| fruit, apricot, canned, home canned | Fruits, sweetened | Fruits, excluding berries |
| fruit, blueberries, canned, water pack | Fruits, fresh and unsweetened | Berries |
| fruit, blueberries, canned, syrup pack, heavy | Fruits, sweetened | Berries |
| fruit, blueberries, canned, home canned | Fruits, sweetened | Berries |
| fruit, boysenberries, canned | Fruits, sweetened | Berries |
| fruit, cherries, canned, sweet, water pack | Fruits, fresh and unsweetened | Fruits, excluding berries |
| fruit, cherries, canned, sweet, juice pack | Fruits, fresh and unsweetened | Fruits, excluding berries |
| fruit, cherries, canned, sweet, syrup pack, light | Fruits, sweetened | Fruits, excluding berries |
| fruit, cherries, canned, sweet, syrup pack, heavy, not drained | Fruits, sweetened | Fruits, excluding berries |
| fruit, cherries, canned, sweet, home canned | Fruits, sweetened | Fruits, excluding berries |
| fruit, fruit cocktail, canned, water pack | Fruits, fresh and unsweetened | Mixtures of two or more fruits |
| fruit, fruit cocktail, canned, juice pack | Fruits, fresh and unsweetened | Mixtures of two or more fruits |
| fruit, fruit cocktail, canned, syrup pack, light | Fruits, sweetened | Mixtures of two or more fruits |
| fruit, fruit cocktail, canned, syrup pack, heavy, not drained | Fruits, sweetened | Mixtures of two or more fruits |
| fruit, elderberries, canned | Fruits, fresh and unsweetened | Berries |
| fruit, figs, canned, water pack | Fruits, fresh and unsweetened | Fruits, excluding berries |
| fruit, figs, canned, syrup pack, light | Fruits, sweetened | Fruits, excluding berries |
| fruit, figs, canned, syrup pack, heavy | Fruits, sweetened | Fruits, excluding berries |
| fruit, figs, canned, home canned | Fruits, sweetened | Fruits, excluding berries |
| fruit, gooseberries, canned | Fruits, sweetened | Berries |
| fruit, grapefruit, canned, water pack | Fruits, fresh and unsweetened | Citrus fruits |
| fruit, grapefruit, canned, juice pack | Fruits, fresh and unsweetened | Citrus fruits |
| fruit, grapefruit, canned, syrup pack | Fruits, sweetened | Citrus fruits |
| fruit, grapes, canned, water pack | Fruits, fresh and unsweetened | Fruits, excluding berries |
| fruit, grapes, canned, syrup pack | Fruits, sweetened | Fruits, excluding berries |
| fruit, grapes, canned, home canned | Fruits, sweetened | Fruits, excluding berries |
| fruit, mandarin orange, canned, juice pack, not drained | Fruits, fresh and unsweetened | Citrus fruits |
| fruit, mandarin orange, canned, syrup pack | Fruits, sweetened | Citrus fruits |
| fruit, orange, canned sections | Fruits, sweetened | Citrus fruits |
| fruit, peach, canned, water pack | Fruits, fresh and unsweetened | Fruits, excluding berries |
| fruit, peach, canned, juice pack | Fruits, fresh and unsweetened | Fruits, excluding berries |
| fruit, peach, canned, syrup pack, extra light | Fruits, sweetened | Fruits, excluding berries |
| fruit, peach, canned, syrup pack, light | Fruits, sweetened | Fruits, excluding berries |
| fruit, peach, canned, syrup pack, heavy, not drained | Fruits, sweetened | Fruits, excluding berries |
| fruit, peach, canned, home canned | Fruits, sweetened | Fruits, excluding berries |
| fruit, pear, canned, water pack | Fruits, fresh and unsweetened | Fruits, excluding berries |
| fruit, pear, canned, juice pack | Fruits, fresh and unsweetened | Fruits, excluding berries |
| fruit, pear, canned, syrup pack, light | Fruits, sweetened | Fruits, excluding berries |
| fruit, pear, canned, syrup pack, heavy, not drained | Fruits, sweetened | Fruits, excluding berries |
| fruit, pear, canned, home canned | Fruits, sweetened | Fruits, excluding berries |
| fruit, pineapple, canned, water pack | Fruits, fresh and unsweetened | Fruits, excluding berries |
| fruit, pineapple, canned, juice pack, not drained | Fruits, fresh and unsweetened | Fruits, excluding berries |
| fruit, pineapple, canned, syrup pack, light | Fruits, sweetened | Fruits, excluding berries |
| fruit, pineapple, canned, syrup pack, heavy | Fruits, sweetened | Fruits, excluding berries |
| fruit, pineapple, canned, home canned | Fruits, sweetened | Fruits, excluding berries |
| fruit, plum, canned, water pack | Fruits, fresh and unsweetened | Fruits, excluding berries |
| fruit, plum, canned, juice pack | Fruits, fresh and unsweetened | Fruits, excluding berries |
| fruit, plum, canned, syrup pack, light | Fruits, sweetened | Fruits, excluding berries |
| fruit, plum, canned, syrup pack, heavy, not drained | Fruits, sweetened | Fruits, excluding berries |
| fruit, plum, canned, home canned | Fruits, sweetened | Fruits, excluding berries |
| fruit, prune, canned | Fruits, sweetened | Fruits, excluding berries |
| fruit, raspberries, canned, water pack | Fruits, fresh and unsweetened | Berries |
| fruit, raspberries, canned, syrup pack | Fruits, sweetened | Berries |
| fruit, raspberries, canned, home canned | Fruits, sweetened | Berries |
| fruit, rhubarb, canned, water pack | Fruits, fresh and unsweetened | Fruits, excluding berries |
| fruit, rhubarb, canned, syrup pack, light | Fruits, sweetened | Fruits, excluding berries |
| fruit, rhubarb, canned, syrup pack, heavy | Fruits, sweetened | Fruits, excluding berries |
| fruit, rhubarb, canned, home canned | Fruits, sweetened | Fruits, excluding berries |
| fruit, strawberries, canned, water pack | Fruits, fresh and unsweetened | Berries |
| fruit, strawberries, canned, syrup pack | Fruits, sweetened | Berries |
| mixed dish, macaroni and cheese, plain, canned or frozen | Pasta and rice (includes recipes) | Mixtures, mainly grain, pasta, or bread |
| mixed dish, ravioli, cheese filled, with tomato sauce, canned | Pasta and rice (includes recipes) | Mixtures, mainly grain, pasta, or bread |
| mixed dish, ravioli, meat filled, with tomato sauce, canned | Meat, poultry, and fish recipes | Mixtures, mainly grain, pasta, or bread |
| gravy, chicken or turkey, canned, regular | Gravy and sauces | Gravies from meat, poultry, fish base |
| gravy, unknown type of meat or poultry, canned, regular | Gravy and sauces | Gravies from meat, poultry, fish base |
| beef, corned beef, canned | Beef | Beef roasts, stew meat, corned beef, beef brisket, sandwich steaks |
| poultry, turkey, canned | Poultry | Turkey |
| pork, ham, canned, regular, visible fat eaten | Pork | Ham |
| pork, ham, canned, regular, no visible fat eaten | Pork | Ham |
| pork, ham, canned, extra lean (approx 5% fat) | Pork | Ham |
| vegetables, asparagus, canned - drained, regular | Cooked vegetables, fresh, frozen or canned | Other vegetables, cooked |
| vegetables, beans, green or string beans, canned - drained, regular | Cooked vegetables, fresh, frozen or canned | Other vegetables, cooked |
| vegetables, beans, lima beans, canned - drained, regular | Mature dried beans and peas | Other vegetables, cooked |
| vegetables, beans, wax or yellow beans, canned - drained, regular | Cooked vegetables, fresh, frozen or canned | Other vegetables, cooked |
| vegetables, beets, canned - drained, regular | Cooked vegetables, fresh, frozen or canned | Other vegetables, cooked |
| vegetables, carrots, canned - drained, regular | Cooked vegetables, fresh, frozen or canned | Carrots |
| vegetables, Chinese vegetables - canned and drained, chow mein - without salt | Cooked vegetables, fresh, frozen or canned | Other vegetables, cooked |
| vegetables, corn, yellow, canned, cream style | Cooked vegetables, fresh, frozen or canned | Other vegetables, cooked |
| vegetables, mixed and/or combination vegetables, corn, lima beans, peas, green beans and carrots - plain, canned - drained, regular | Cooked vegetables, fresh, frozen or canned | Other vegetable mixtures, cooked |
| vegetables, mixed and/or combination vegetables, peas and carrots, canned - drained, regular | Cooked vegetables, fresh, frozen or canned | Carrots |
| vegetables, mushrooms, canned - drained, regular | Cooked vegetables, fresh, frozen or canned | Other vegetables, cooked |
| vegetables, peas, green peas, canned - drained, regular | Cooked vegetables, fresh, frozen or canned | Other vegetables, cooked |
| vegetables, potato, canned - drained, regular | Cooked vegetables, fresh, frozen or canned | White potatoes, baked and boiled |
| vegetables, spinach, canned - drained, regular | Cooked vegetables, fresh, frozen or canned | Dark-green leafy vegetables |
| beverages, juice or flavored drink, lemon juice, canned, bottled, or boxed | Fruit juices and drinks | Citrus fruit juices |
| beverages, juice or flavored drink, lime juice, canned, bottled, or boxed | Fruit juices and drinks | Citrus fruit juices |
| grains, bulgur, canned | Cooked cereals, prepared and unprepared | Cooked cereals, rice |
| fish and seafood, abalone, canned | Shellfish | Shellfish |
| fish and seafood, anchovy, smoked - canned in oil and drained | Fish and fish roe | Finfish |
| fish and seafood, clams, canned - drained | Shellfish | Shellfish |
| fish and seafood, crab, canned - drained | Shellfish | Shellfish |
| fish and seafood, herring, smoked or kippered - canned and drained | Fish and fish roe | Finfish |
| fish and seafood, lobster, canned - drained | Shellfish | Shellfish |
| fish and seafood, mackerel, canned - drained | Fish and fish roe | Finfish |
| fish and seafood, oyster, canned - drained | Shellfish | Shellfish |
| fish and seafood, salmon, canned - drained, pink, with salt | Fish and fish roe | Finfish |
| fish and seafood, salmon, canned - drained, pink, without salt | Fish and fish roe | Finfish |
| fish and seafood, sardines, canned in oil, drained | Fish and fish roe | Finfish |
| fish and seafood, sardines, canned in oil, not drained | Fish and fish roe | Finfish |
| fish and seafood, sardines, canned in tomato sauce | Fish and fish roe | Meat, poultry, fish with gravy or sauce or creamed |
| lunchmeats and sausages, canned, chopped or spiced ham, loaf (e.g. Spam), regular, regular | Cold cuts and sausage - pork | Frankfurters, sausages, lunchmeats, meat spreads |
| lunchmeats and sausages, canned, deviled ham | Cold cuts and sausage - pork | Frankfurters, sausages, lunchmeats, meat spreads |
| lunchmeats and sausages, canned, ham patties | Cold cuts and sausage - pork | Frankfurters, sausages, lunchmeats, meat spreads |
| lunchmeats and sausages, canned, ham salad spread | Cold cuts and sausage - pork | Frankfurters, sausages, lunchmeats, meat spreads |
| lunchmeats and sausages, canned, ham and cheese spread | Cold cuts and sausage - pork | Frankfurters, sausages, lunchmeats, meat spreads |
| lunchmeats and sausages, canned, chicken spread | Cold cuts and sausage - poultry | Frankfurters, sausages, lunchmeats, meat spreads |
| lunchmeats and sausages, canned, sandwich spread (meat-based) | Cold cuts and sausage - combinations | Frankfurters, sausages, lunchmeats, meat spreads |
| fish and seafood, shrimp, canned - drained, with salt | Shellfish | Shellfish |
| fish and seafood, shrimp, canned - drained, without salt | Shellfish | Shellfish |
| fish and seafood, tuna, canned, light, oil pack, regular, not drained | Fish and fish roe | Finfish |
| fish and seafood, tuna, canned, light, oil pack, regular, drained | Fish and fish roe | Finfish |
| fish and seafood, tuna, canned, light, oil pack, regular, drained and rinsed | Fish and fish roe | Finfish |
| fish and seafood, tuna, canned, light, oil pack, low sodium, not drained | Fish and fish roe | Finfish |
| fish and seafood, tuna, canned, light, oil pack, low sodium, drained | Fish and fish roe | Finfish |
| fish and seafood, tuna, canned, light, oil pack, low sodium, drained and rinsed | Fish and fish roe | Finfish |
| fish and seafood, tuna, canned, light, water pack, regular, drained and rinsed | Fish and fish roe | Finfish |
| fish and seafood, tuna, canned, light, water pack, regular, drained - not rinsed | Fish and fish roe | Finfish |
| fish and seafood, tuna, canned, light, water pack, low sodium, drained and rinsed | Fish and fish roe | Finfish |
| fish and seafood, tuna, canned, light, water pack, low sodium, drained - not rinsed | Fish and fish roe | Finfish |
| fish and seafood, tuna, canned, light, water pack, no salt - drained | Fish and fish roe | Finfish |
| lunchmeats and sausages, ham, chopped, not canned | Cold cuts and sausage - pork | Frankfurters, sausages, lunchmeats, meat spreads |
| fruit, cranberries, canned, whole berries | Fruits, sweetened | Berries |
| fruit, cranberries, canned, jellied | Fruits, sweetened | Berries |
| fruit, blackberries, canned | Fruits, sweetened | Berries |
| vegetables, beans, garbanzo beans, canned - drained, low sodium | Mature dried beans and peas | Dried peas, lentils, and mixtures |
| vegetables, beans, kidney beans, canned - drained, low sodium | Mature dried beans and peas | Dried beans |
| vegetables, beans, navy beans, canned - drained, low sodium | Mature dried beans and peas | Dried beans |
| vegetables, beans, great northern, canned - drained, low sodium | Mature dried beans and peas | Dried beans |
| vegetables, beans, pinto beans, canned - drained, low sodium | Mature dried beans and peas | Dried beans |
| vegetables, lentils, canned - drained, low sodium | Mature dried beans and peas | Dried peas, lentils, and mixtures |
| vegetables, lentils, canned - drained, regular | Mature dried beans and peas | Dried peas, lentils, and mixtures |
| vegetables, peas, split peas - yellow or green, canned - drained, low sodium | Mature dried beans and peas | Dried peas, lentils, and mixtures |
| vegetables, peas, split peas - yellow or green, canned - drained, regular | Mature dried beans and peas | Dried peas, lentils, and mixtures |
| vegetables, beans, soybeans, canned - drained, low sodium | Mature dried beans and peas | Dried beans |
| vegetables, beans, soybeans, canned - drained, regular | Mature dried beans and peas | Dried beans |
| vegetables, beans, brown, canned - drained, regular | Mature dried beans and peas | Dried beans |
| vegetables, beans, brown, canned - drained, low sodium | Mature dried beans and peas | Dried beans |
| vegetables, beans, black, canned - drained, low sodium | Mature dried beans and peas | Dried beans |
| vegetables, beans, black, canned - drained, regular | Mature dried beans and peas | Dried beans |
| vegetables, tomato, canned, regular, plain | Cooked vegetables, fresh, frozen or canned | Tomatoes, cooked |
| vegetables, yams (sweet potato type), canned, regular, syrup packed - drained | Cooked vegetables, fresh, frozen or canned | Sweet potatoes |
| vegetables, yams (sweet potato type), canned, low sodium - drained | Cooked vegetables, fresh, frozen or canned | Sweet potatoes |
| poultry, chicken, canned | Poultry | Chicken |
| vegetables, peppers, jalapeno pepper, canned - drained | Cooked vegetables, fresh, frozen or canned | Other vegetables, cooked |
| grains, hominy, canned | Cooked cereals, prepared and unprepared | Other vegetables, cooked |
| vegetables, yams (sweet potato type), unknown if baked, boiled, frozen, or canned | Cooked vegetables, fresh, frozen or canned | Sweet potatoes |
| mixed dish, Pima Indian, beef meatball stew - canned | Meat, poultry, and fish recipes | Meat, poultry, fish with starch item and vegetables |
| pork, canned | Pork | Pork, NFS, ground, dehydrated |
| beef, canned | Beef | Beef roasts, stew meat, corned beef, beef brisket, sandwich steaks |
| vegetables, Pima Indian, vegetarian beans - canned | Mature dried beans and peas | Dried beans |
| vegetables, sweet potato, canned, regular, syrup packed - drained | Cooked vegetables, fresh, frozen or canned | Sweet potatoes |
| vegetables, sweet potato, canned, regular, vacuum packed | Cooked vegetables, fresh, frozen or canned | Sweet potatoes |
| vegetables, sweet potato, canned, low sodium - drained | Cooked vegetables, fresh, frozen or canned | Sweet potatoes |
| lunchmeats and sausages, canned, chopped or spiced ham, loaf (e.g. Spam), low fat | Cold cuts and sausage - combinations | Frankfurters, sausages, lunchmeats, meat spreads |
| vegetables, mixed and/or combination vegetables, corn, lima beans, peas, green beans and carrots - plain, canned - drained, low sodium | Cooked vegetables, fresh, frozen or canned | Other vegetable mixtures, cooked |
| vegetables, mixed and/or combination vegetables, peas and carrots, canned - drained, low sodium | Cooked vegetables, fresh, frozen or canned | Carrots |
| fish and seafood, tuna, canned, light, oil pack, no salt - drained | Fish and fish roe | Finfish |
| vegetables, Chinese vegetables - canned and drained, chop suey - with salt | Cooked vegetables, fresh, frozen or canned | Other vegetables, cooked |
| fish and seafood, sardines, canned in water - drained | Fish and fish roe | Finfish |
| milk, mixtures and milk drinks, chocolate, purchased ready-to-drink, regular, brands, Sego Liquid Diet Food - canned, Very Chocolate flavors | Milk-based meal replacement/supplement beverages | Milk-based meal replacements, fluid |
| milk, mixtures and milk drinks, strawberry and other flavors, purchased ready-to-drink, regular, brands, Sego Liquid Diet Food - canned, Very Strawberry | Milk-based meal replacement/supplement beverages | Milk-based meal replacements, fluid |
| vegetables, artichoke, regular globe or French (hearts), canned, marinated in oil mixture | Vegetable recipes | Other vegetables, cooked |
| vegetables, beans, broadbeans, canned - drained, regular | Mature dried beans and peas | Dried beans |
| vegetables, beans, broadbeans, canned - drained, low sodium | Mature dried beans and peas | Dried beans |
| gravy, pork, canned, regular | Gravy and sauces | Gravies from meat, poultry, fish base |
| lunchmeats and sausages, canned, potted meat | Cold cuts and sausage - combinations | Frankfurters, sausages, lunchmeats, meat spreads |
| vegetables, hearts of palm - canned | Cooked vegetables, fresh, frozen or canned | Other vegetables, cooked |
| vegetables, beans, refried beans, canned, fat free | Vegetable recipes | Dried bean mixtures |
| vegetables, potato, unknown if baked, boiled, canned, or roasted, with skin | Cooked vegetables, fresh, frozen or canned | White potatoes, NFS |
| vegetables, potato, unknown if baked, boiled, canned, or roasted, without skin | Cooked vegetables, fresh, frozen or canned | White potatoes, NFS |
| mixed dish, stew, chicken, canned | Meat, poultry, and fish recipes | Meat, poultry, fish with starch item and vegetables |
| mixed dish, stew, beef, canned | Meat, poultry, and fish recipes | Meat, poultry, fish with starch item and vegetables |
| mixed dish, a la king - chicken, canned | Meat, poultry, and fish recipes | Meat, poultry, fish with vegetables (excluding white potatoes) |
| mixed dish, chili, with beans and meat or poultry, regular, canned, beef, regular | Meat, poultry, and fish recipes | Meat, poultry, fish with gravy or sauce or creamed |
| mixed dish, chili, with meat or poultry - no beans, canned, beef, regular | Meat, poultry, and fish recipes | Meat, poultry, fish with gravy or sauce or creamed |
| mixed dish, chow mein (noodles not included), beef, canned | Meat, poultry, and fish recipes | Meat, poultry, fish with vegetables (excluding white potatoes) |
| mixed dish, chow mein (noodles not included), beef, unknown if from recipe or canned | Meat, poultry, and fish recipes | Meat, poultry, fish with vegetables (excluding white potatoes) |
| mixed dish, chow mein (noodles not included), chicken, canned | Meat, poultry, and fish recipes | Meat, poultry, fish with vegetables (excluding white potatoes) |
| mixed dish, chow mein (noodles not included), chicken, unknown if from recipe or canned | Meat, poultry, and fish recipes | Meat, poultry, fish with vegetables (excluding white potatoes) |
| mixed dish, hash, roast beef, canned | Meat, poultry, and fish recipes | Meat, poultry, fish with starch item (includes white potatoes) |
| mixed dish, spaghetti - main dish (noodles and sauce), with tomato sauce, with meat, canned (e.g. SpaghettiOs) | Meat, poultry, and fish recipes | Mixtures, mainly grain, pasta, or bread |
| mixed dish, spaghetti - main dish (noodles and sauce), with tomato sauce, with frankfurters, canned (e.g. SpaghettiOs) | Meat, poultry, and fish recipes | Mixtures, mainly grain, pasta, or bread |
| mixed dish, spaghetti - main dish (noodles and sauce), with tomato sauce, without meat or poultry, canned (e.g. SpaghettiOs) | Pasta and rice (includes recipes) | Mixtures, mainly grain, pasta, or bread |
| mixed dish, hash, corned beef, canned | Meat, poultry, and fish recipes | Meat, poultry, fish with starch item (includes white potatoes) |
| mixed dish, macaroni and beef with tomato sauce - canned | Meat, poultry, and fish recipes | Meat, poultry, fish with starch item (includes white potatoes) |
| mixed dish, tamale, canned | Meat, poultry, and fish recipes | Mixtures, mainly grain, pasta, or bread |
| mixed dish, chow mein (noodles not included), pork, canned | Meat, poultry, and fish recipes | Meat, poultry, fish with vegetables (excluding white potatoes) |
| mixed dish, chow mein (noodles not included), pork, unknown if from recipe or canned | Meat, poultry, and fish recipes | Meat, poultry, fish with vegetables (excluding white potatoes) |
| mixed dish, lasagna, canned | Meat, poultry, and fish recipes | Mixtures, mainly grain, pasta, or bread |
| beverages, coffee, cappuccino, purchased ready-to-drink (bottled or canned) | Coffee | Coffees |
| milk, mixtures and milk drinks, chocolate, purchased ready-to-drink, light, Sego Lite Liquid Diet Food - canned, chocolate flavors | Milk-based meal replacement/supplement beverages | Milk-based meal replacements, fluid |
| milk, mixtures and milk drinks, strawberry and other flavors, purchased ready-to-drink, regular, brands, Sego Liquid Diet Food - canned, Very Vanilla | Milk-based meal replacement/supplement beverages | Milk-based meal replacements, fluid |
| milk, mixtures and milk drinks, strawberry and other flavors, purchased ready-to-drink, light, brands, Sego Lite Liquid Diet Food - canned, French Vanilla | Milk-based meal replacement/supplement beverages | Milk-based meal replacements, fluid |
| milk, mixtures and milk drinks, strawberry and other flavors, purchased ready-to-drink, light, brands, Sego Lite Liquid Diet Food - canned, Strawberry | Milk-based meal replacement/supplement beverages | Milk-based meal replacements, fluid |
| milk, mixtures and milk drinks, strawberry and other flavors, purchased ready-to-drink, light, brands, Sego Lite Liquid Diet Food - canned, Vanilla | Milk-based meal replacement/supplement beverages | Milk-based meal replacements, fluid |
| vegetables, yams (sweet potato type), canned, regular, vacuum packed | Cooked vegetables, fresh, frozen or canned | Sweet potatoes |
| milk, soy milk, canned, fat free - sweetened | Polyunsaturated vegetable fat - filled milk | Milk, fluid, imitation |
| vegetables, artichoke, regular globe or French (hearts), canned, in water | Cooked vegetables, fresh, frozen or canned | Other vegetables, cooked |
| vegetables, tomato, canned, regular, stewed (with onions, green peppers, and celery) | Vegetable recipes | Tomatoes, cooked |
| salad, bean, three bean, canned (no fat) | Vegetable recipes | Other vegetable mixtures, cooked |
| gravy, beef, canned, fat free | Gravy and sauces | Gravies from meat, poultry, fish base |
| gravy, chicken or turkey, canned, fat free | Gravy and sauces | Gravies from meat, poultry, fish base |
| gravy, pork, canned, fat free | Gravy and sauces | Gravies from meat, poultry, fish base |
| gravy, unknown type of meat or poultry, canned, fat free | Gravy and sauces | Gravies from meat, poultry, fish base |
| vegetables, collards, cooked from canned | Cooked vegetables, fresh, frozen or canned | Dark-green leafy vegetables |
| lunchmeats and sausages, canned, chopped or spiced ham, loaf (e.g. Spam), regular, low sodium | Cold cuts and sausage - combinations | Frankfurters, sausages, lunchmeats, meat spreads |
| lunchmeats and sausages, canned, chopped or spiced ham, spread | Cold cuts and sausage - combinations | Frankfurters, sausages, lunchmeats, meat spreads |
| vegetables, peppers, hot chili, red, canned - drained | Cooked vegetables, fresh, frozen or canned | Other vegetables, cooked |
| mixed dish, chili, with beans - meatless, canned | Vegetable recipes | Dried bean mixtures |
| mixed dish, chili, with beans and meat or poultry, regular, canned, turkey | Meat, poultry, and fish recipes | Meat, poultry, fish with gravy or sauce or creamed |
| mixed dish, chili, with meat or poultry - no beans, canned, turkey | Meat, poultry, and fish recipes | Meat, poultry, fish with gravy or sauce or creamed |
| fruit, apricot, canned, syrup pack, heavy, drained | Fruits, sweetened | Fruits, excluding berries |
| fruit, cherries, canned, sweet, syrup pack, heavy, drained | Fruits, sweetened | Fruits, excluding berries |
| fruit, fruit cocktail, canned, syrup pack, heavy, drained | Fruits, sweetened | Mixtures of two or more fruits |
| fruit, mandarin orange, canned, juice pack, drained | Fruits, fresh and unsweetened | Citrus fruits |
| fruit, peach, canned, syrup pack, heavy, drained | Fruits, sweetened | Fruits, excluding berries |
| fruit, pear, canned, syrup pack, heavy, drained | Fruits, sweetened | Fruits, excluding berries |
| fruit, pineapple, canned, juice pack, drained | Fruits, fresh and unsweetened | Fruits, excluding berries |
| fruit, plum, canned, syrup pack, heavy, drained | Fruits, sweetened | Fruits, excluding berries |
| fruit, apple, canned, unsweetened | Fruits, fresh and unsweetened | Fruits, excluding berries |
| fish and seafood, Alaska Native, salmon, king or chinook, kippered - canned | Fish and fish roe | Finfish |
| fish and seafood, Alaska Native, salmon, king or chinook, smoked and canned | Fish and fish roe | Finfish |
| vegetables, grape leaves (canned) | Cooked vegetables, fresh, frozen or canned | Dark-green leafy vegetables |
| vegetables, mushrooms, straw - canned, drained | Cooked vegetables, fresh, frozen or canned | Other vegetables, cooked |
| fish and seafood, Alaska Native, salmon, red (sockeye), canned - bones removed | Fish and fish roe | Finfish |
| fish and seafood, Alaska Native, salmon, red (sockeye), canned - smoked | Fish and fish roe | Finfish |
| fish and seafood, Alaska Native, steelhead trout - boiled, canned | Fish and fish roe | Finfish |
| fruit, jackfruit, canned - syrup pack | Fruits, sweetened | Fruits, excluding berries |
| fruit, mangosteen, canned - syrup pack | Fruits, sweetened | Fruits, excluding berries |
| fruit, rambutan - canned, syrup pack | Fruits, sweetened | Fruits, excluding berries |
| nuts and seeds, ginkgo nuts, canned | Nuts and nut butters | Nuts |
| fruit, cherries, canned, sour, water pack, not drained | Fruits, fresh and unsweetened | Fruits, excluding berries |
| fruit, cherries, canned, sour, syrup pack, light | Fruits, sweetened | Fruits, excluding berries |
| fruit, cherries, canned, sour, syrup pack, heavy | Fruits, sweetened | Fruits, excluding berries |
| beverages, coffee, frappuccino, purchased ready-to-drink (bottled or canned), regular | Coffee | Coffees |
| beverages, coffee, frappuccino, purchased ready-to-drink (bottled or canned), light | Coffee | Coffees |
| fruit, blueberries, canned, syrup pack, light (drained) | Fruits, sweetened | Berries |
| fruit, blueberries, wild, canned (heavy syrup, drained) | Fruits, sweetened | Berries |
| vegetables, beans, refried beans, canned, vegetarian | Vegetable recipes | Dried bean mixtures |
| vegetables, beans, refried beans, canned, red | Vegetable recipes | Dried bean mixtures |
| vegetables, corn, white, canned, regular - drained | Cooked vegetables, fresh, frozen or canned | Other vegetables, cooked |
| vegetables, corn, white, canned, low sodium - drained | Cooked vegetables, fresh, frozen or canned | Other vegetables, cooked |
| vegetables, corn, white, canned, cream style | Cooked vegetables, fresh, frozen or canned | Other vegetables, cooked |
| vegetables, corn, yellow and white, canned, regular - drained | Cooked vegetables, fresh, frozen or canned | Other vegetables, cooked |
| vegetables, corn, yellow and white, canned, low sodium - drained | Cooked vegetables, fresh, frozen or canned | Other vegetables, cooked |
| vegetables, corn, yellow and white, canned, cream style | Cooked vegetables, fresh, frozen or canned | Other vegetables, cooked |
| fruit, papaya, canned (heavy syrup, drained) | Fruits, sweetened | Fruits, excluding berries |
| fish and seafood, tuna, canned, white albacore, oil pack, regular, not drained | Fish and fish roe | Finfish |
| fish and seafood, tuna, canned, white albacore, oil pack, regular, drained | Fish and fish roe | Finfish |
| fish and seafood, tuna, canned, white albacore, oil pack, regular, drained and rinsed | Fish and fish roe | Finfish |
| fish and seafood, tuna, canned, white albacore, oil pack, low sodium, not drained | Fish and fish roe | Finfish |
| fish and seafood, tuna, canned, white albacore, oil pack, low sodium, drained | Fish and fish roe | Finfish |
| fish and seafood, tuna, canned, white albacore, oil pack, low sodium, drained and rinsed | Fish and fish roe | Finfish |
| fish and seafood, tuna, canned, white albacore, oil pack, no salt - drained | Fish and fish roe | Finfish |
| fish and seafood, tuna, canned, white albacore, water pack, regular, drained and rinsed | Fish and fish roe | Finfish |
| fish and seafood, tuna, canned, white albacore, water pack, regular, drained - not rinsed | Fish and fish roe | Finfish |
| fish and seafood, tuna, canned, white albacore, water pack, low sodium, drained and rinsed | Fish and fish roe | Finfish |
| fish and seafood, tuna, canned, white albacore, water pack, low sodium, drained - not rinsed | Fish and fish roe | Finfish |
| fish and seafood, tuna, canned, white albacore, water pack, no salt - drained | Fish and fish roe | Finfish |
| vegetables, peas, green peas, canned - drained, regular (drained and rinsed) | Cooked vegetables, fresh, frozen or canned | Other vegetables, cooked |
| vegetables, corn, yellow, canned, regular - drained and rinsed | Cooked vegetables, fresh, frozen or canned | Other vegetables, cooked |
| vegetables, beans, garbanzo beans, canned - drained, regular (drained and rinsed) | Mature dried beans and peas | Dried peas, lentils, and mixtures |
| vegetables, beans, kidney beans, canned - drained, regular (drained and rinsed) | Mature dried beans and peas | Dried beans |
| vegetables, beans, pinto beans, canned - drained, regular (drained and rinsed) | Mature dried beans and peas | Dried beans |
| fruit, tropical fruit mixture, canned, juice pack | Fruits, fresh and unsweetened | Mixtures of two or more fruits |
| fruit, tropical fruit mixture, canned, syrup pack | Fruits, sweetened | Mixtures of two or more fruits |
| vegetables, beans, refried beans, canned, reduced sodium | Vegetable recipes | Dried bean mixtures |
| fruit, cherries, canned, sour, water pack, drained | Fruits, fresh and unsweetened | Fruits, excluding berries |
| fish and seafood, salmon, canned - drained, chum, with salt | Fish and fish roe | Finfish |
| fish and seafood, salmon, canned - drained, chum, without salt | Fish and fish roe | Finfish |
| fish and seafood, salmon, canned - drained, sockeye (red), with salt | Fish and fish roe | Finfish |
| fish and seafood, salmon, canned - drained, sockeye (red), without salt | Fish and fish roe | Finfish |
| fruit, nance, canned (syrup, drained) | Fruits, sweetened | Fruits, excluding berries |
| vegetables, peppers, sweet red, canned | Cooked vegetables, fresh, frozen or canned | Other vegetables, cooked |
| mixed dish, chili, with beans and meat or poultry, regular, canned, beef, less sodium | Meat, poultry, and fish recipes | Meat, poultry, fish with gravy or sauce or creamed |
| mixed dish, chili, with beans and meat or poultry, regular, canned, chicken | Meat, poultry, and fish recipes | Meat, poultry, fish with gravy or sauce or creamed |
| mixed dish, chili, with meat or poultry - no beans, canned, beef, less sodium | Meat, poultry, and fish recipes | Meat, poultry, fish with gravy or sauce or creamed |

# **Supplementary Table S2: Packaged Food**

| **Food Description** | **NCC Database Food Group Description** | **USDA Food Group Description** |
| --- | --- | --- |
| desserts - miscellaneous, snacks - commercial packaged, brand name listing, Pillsbury, Pillsbury Turnover - Apple | Commercial snack - miscellaneous desserts | Cobblers, eclairs, turnovers, other pastries |
| desserts - miscellaneous, snacks - commercial packaged, brand name listing, Hostess, Hostess Cupcake - Chocolate | Commercial snack cakes and muffins | Cakes |
| desserts - miscellaneous, snacks - commercial packaged, brand name listing, Hostess, Hostess Sno Balls | Commercial snack cakes and muffins | Cakes |
| desserts - miscellaneous, snacks - commercial packaged, brand name listing, Hostess, Hostess Ding Dongs | Commercial snack cakes and muffins | Cakes |
| desserts - miscellaneous, snacks - commercial packaged, brand name listing, Hostess, Hostess Ho Hos | Commercial snack cakes and muffins | Cakes |
| desserts - miscellaneous, snacks - commercial packaged, brand name listing, Tastykake, Tastykake Junior - Chocolate | Commercial snack cakes and muffins | Cakes |
| desserts - miscellaneous, snacks - commercial packaged, brand name listing, Little Debbie, Little Debbie Devil Squares | Commercial snack cakes and muffins | Cakes |
| desserts - miscellaneous, snacks - commercial packaged, brand name listing, Little Debbie, Little Debbie Easter Basket Cakes - Chocolate | Commercial snack cakes and muffins | Cakes |
| desserts - miscellaneous, snacks - commercial packaged, brand name listing, Little Debbie, Little Debbie Dessert Cakes - Yellow | Commercial snack cakes and muffins | Cakes |
| desserts - miscellaneous, snacks - commercial packaged, brand name listing, Tastykake, Tastykake Junior - Coconut | Commercial snack cakes and muffins | Cakes |
| desserts - miscellaneous, snacks - commercial packaged, brand name listing, Hostess, Hostess Twinkies | Commercial snack cakes and muffins | Cakes |
| desserts - miscellaneous, snacks - commercial packaged, brand name listing, Drake's, Drake's Yankee Doodles | Commercial snack cakes and muffins | Cakes |
| desserts - miscellaneous, snacks - commercial packaged, brand name listing, Tastykake, Tastykake Cupcake - Chocolate Lover's | Commercial snack cakes and muffins | Cakes |
| desserts - miscellaneous, snacks - commercial packaged, brand name listing, Hostess, Hostess Crumb Cake | Commercial snack cakes and muffins | Coffee cakes, not yeast |
| desserts - miscellaneous, snacks - commercial packaged, brand name listing, Drake's, Drake's Devil Dogs | Commercial snack cakes and muffins | Cakes |
| desserts - miscellaneous, snacks - commercial packaged, brand name listing, Tastykake, Tastykake Krimpet - Butterscotch | Commercial snack cakes and muffins | Cakes |
| desserts - miscellaneous, snacks - commercial packaged, brand name listing, Tastykake, Tastykake Krimpet - Jelly | Commercial snack cakes and muffins | Cakes |
| desserts - miscellaneous, snacks - commercial packaged, brand name listing, Hostess, Hostess Suzy Q's - Banana | Commercial snack cakes and muffins | Cakes |
| ice cream and frozen desserts, sherbet, packaged with ice cream | Ice cream, ice milk, sherbet, nondairy frozen dessert, and milkshakes | Milk desserts, frozen |
| ice cream and frozen desserts, sherbet, packaged with ice milk | Ice cream, ice milk, sherbet, nondairy frozen dessert, and milkshakes | Milk desserts, frozen |
| ice cream and frozen desserts, treats (frozen prepackaged), brand name listing, Dole, Dole Fruit 'N Juice Bar - all flavors | Frozen treats | Mixtures of fruits and nonfruit items |
| ice cream and frozen desserts, treats (frozen prepackaged), brand name listing, Eskimo Pie, Eskimo Pie - original | Frozen treats | Milk desserts, frozen |
| ice cream and frozen desserts, treats (frozen prepackaged), brand name listing, Haagen-Dazs, Haagen-Dazs Ice Cream Bar - vanilla & milk chocolate | Frozen treats | Milk desserts, frozen |
| ice cream and frozen desserts, treats (frozen prepackaged), brand name listing, Nestle, Heath Ice Cream Bar | Frozen treats | Milk desserts, frozen |
| ice cream and frozen desserts, treats (frozen prepackaged), brand name listing, Weight Watchers, Weight Watchers Vanilla Sandwich Bar | Frozen treats | Milk desserts, frozen |
| ice cream and frozen desserts, treats (frozen prepackaged), brand name listing, Weight Watchers, Weight Watchers Chocolate Treat Bar | Frozen treats | Milk desserts, frozen |
| ice cream and frozen desserts, treats (frozen prepackaged), brand name listing, Welch's, Welch's Fruit Juice Bar - all flavors | Frozen treats | Mixtures of fruits and nonfruit items |
| ice cream and frozen desserts, treats (frozen prepackaged), ice cream bar, regular, plain | Frozen treats | Milk desserts, frozen |
| ice cream and frozen desserts, treats (frozen prepackaged), ice cream bar, regular, with toffee | Frozen treats | Milk desserts, frozen |
| ice cream and frozen desserts, treats (frozen prepackaged), ice cream cookie sandwich | Frozen treats | Milk desserts, frozen |
| ice cream and frozen desserts, treats (frozen prepackaged), ice cream sandwich, regular | Frozen treats | Milk desserts, frozen |
| ice cream and frozen desserts, treats (frozen prepackaged), ice milk bar (chocolate coated), plain | Frozen treats | Milk desserts, frozen |
| ice cream and frozen desserts, treats (frozen prepackaged), Dreamsicle or Creamsicle, regular | Frozen treats | Milk desserts, frozen |
| ice cream and frozen desserts, treats (frozen prepackaged), fruit juice bar, regular | Frozen treats | Mixtures of fruits and nonfruit items |
| ice cream and frozen desserts, treats (frozen prepackaged), fudge type bar (fudgesicle), regular | Frozen treats | Milk desserts, frozen |
| ice cream and frozen desserts, treats (frozen prepackaged), Popsicle, regular | Frozen treats | Ices or popsicles |
| ice cream and frozen desserts, treats (frozen prepackaged), pudding pop | Frozen treats | Puddings, custards, and other milk desserts |
| ice cream and frozen desserts, treats (frozen prepackaged), brand name listing, Haagen-Dazs, Haagen-Dazs Ice Cream Bar - chocolate & dark chocolate | Frozen treats | Milk desserts, frozen |
| desserts - miscellaneous, snacks - commercial packaged, brand name listing, Tastykake, Tastykake Pie - Coconut Creme | Commercial snack pies | Pies |
| desserts - miscellaneous, snacks - commercial packaged, brand name listing, Tastykake, Tastykake Cupcake - Koffee Kake | Commercial snack cakes and muffins | Cakes |
| desserts - miscellaneous, snacks - commercial packaged, brand name listing, Hostess, Hostess Fruit Pie - Pineapple | Commercial snack pies | Pies |
| mixed dish, pizza, from packaged mix, without meat | Miscellaneous grain recipes | Mixtures, mainly grain, pasta, or bread |
| mixed dish, pizza, from packaged mix, with meat | Meat, poultry, and fish recipes | Mixtures, mainly grain, pasta, or bread |
| ice cream and frozen desserts, treats (frozen prepackaged), brand name listing, Kemps, Kemps Kempwich | Frozen treats | Milk desserts, frozen |
| desserts - miscellaneous, snacks - commercial packaged, brand name listing, Hostess, Hostess Honey Bun - Glazed | Commercial snack sweet rolls and coffee cakes | White breads, rolls |
| desserts - miscellaneous, snacks - commercial packaged, brand name listing, Tastykake, Tastykake Creamies - Banana | Commercial snack cakes and muffins | Cakes |
| desserts - miscellaneous, snacks - commercial packaged, brand name listing, Tastykake, Tastykake Creamies - Vanilla | Commercial snack cakes and muffins | Cakes |
| desserts - miscellaneous, snacks - commercial packaged, brand name listing, Tastykake, Tastykake Cupcake - Chocolate | Commercial snack cakes and muffins | Cakes |
| desserts - miscellaneous, snacks - commercial packaged, brand name listing, Lance, Lance Pecan Pie | Commercial snack pies | Pies |
| desserts - miscellaneous, snacks - commercial packaged, brand name listing, Tastykake, Tastykake Kreme Kup | Commercial snack cakes and muffins | Cakes |
| desserts - miscellaneous, snacks - commercial packaged, brand name listing, Tastykake, Tastykake Donuts - Cinnamon | Commercial snack doughnuts | Danish, breakfast pastries, doughnuts, granola bars |
| desserts - miscellaneous, snacks - commercial packaged, brand name listing, Tastykake, Tastykake Donuts - Honey Wheat | Commercial snack doughnuts | Danish, breakfast pastries, doughnuts, granola bars |
| desserts - miscellaneous, snacks - commercial packaged, brand name listing, Tastykake, Tastykake Donuts - Orange Glazed | Commercial snack doughnuts | Danish, breakfast pastries, doughnuts, granola bars |
| desserts - miscellaneous, snacks - commercial packaged, brand name listing, Tastykake, Tastykake Donuts - Powdered Sugar | Commercial snack doughnuts | Danish, breakfast pastries, doughnuts, granola bars |
| desserts - miscellaneous, snacks - commercial packaged, brand name listing, Tastykake, Tastykake Junior - Koffee Kake | Commercial snack cakes and muffins | Cakes |
| desserts - miscellaneous, snacks - commercial packaged, brand name listing, Tastykake, Tastykake Kandy Kake - Chocolate | Commercial snack cakes and muffins | Cakes |
| desserts - miscellaneous, snacks - commercial packaged, brand name listing, Little Debbie, Little Debbie Be My Valentine Cakes - Chocolate | Commercial snack cakes and muffins | Cakes |
| desserts - miscellaneous, snacks - commercial packaged, brand name listing, Little Debbie, Little Debbie Chocolate Twins Cakes | Commercial snack cakes and muffins | Cakes |
| desserts - miscellaneous, snacks - commercial packaged, brand name listing, Little Debbie, Little Debbie Donut Sticks | Commercial snack doughnuts | Danish, breakfast pastries, doughnuts |
| desserts - miscellaneous, snacks - commercial packaged, brand name listing, Little Debbie, Little Debbie Golden Cremes | Commercial snack cakes and muffins | Cakes |
| desserts - miscellaneous, snacks - commercial packaged, brand name listing, Little Debbie, Little Debbie Holiday Snack Cakes - Chocolate Frosted | Commercial snack cakes and muffins | Cakes |
| desserts - miscellaneous, snacks - commercial packaged, brand name listing, Little Debbie, Little Debbie Holiday Snack Cakes - Vanilla Frosted | Commercial snack cakes and muffins | Cakes |
| desserts - miscellaneous, snacks - commercial packaged, brand name listing, Hostess, Hostess Chocodiles | Commercial snack cakes and muffins | Cakes |
| desserts - miscellaneous, snacks - commercial packaged, brand name listing, Hostess, Hostess Cupcake - Orange | Commercial snack cakes and muffins | Cakes |
| desserts - miscellaneous, snacks - commercial packaged, brand name listing, Hostess, Hostess Dessert Cups | Commercial snack cakes and muffins | Cakes |
| desserts - miscellaneous, snacks - commercial packaged, brand name listing, Hostess, Hostess Donettes - Chocolate Frosted | Commercial snack doughnuts | Danish, breakfast pastries, doughnuts, granola bars |
| desserts - miscellaneous, snacks - commercial packaged, brand name listing, Hostess, Hostess Donettes - Powdered | Commercial snack doughnuts | Danish, breakfast pastries, doughnuts, granola bars |
| desserts - miscellaneous, snacks - commercial packaged, brand name listing, Hostess, Hostess Donuts - Plain | Commercial snack doughnuts | Danish, breakfast pastries, doughnuts, granola bars |
| desserts - miscellaneous, snacks - commercial packaged, brand name listing, Hostess, Hostess Donuts - Powdered | Commercial snack doughnuts | Danish, breakfast pastries, doughnuts, granola bars |
| desserts - miscellaneous, snacks - commercial packaged, brand name listing, Hostess, Hostess Donuts - Old Fashioned, Plain | Commercial snack doughnuts | Danish, breakfast pastries, doughnuts, granola bars |
| desserts - miscellaneous, snacks - commercial packaged, brand name listing, Hostess, Hostess Donuts - Old Fashioned, Glazed | Commercial snack doughnuts | Danish, breakfast pastries, doughnuts, granola bars |
| desserts - miscellaneous, snacks - commercial packaged, brand name listing, Hostess, Hostess Lil Angels | Commercial snack cakes and muffins | Cakes |
| desserts - miscellaneous, snacks - commercial packaged, brand name listing, Hostess, Hostess O's - Raspberry Filled Powdered Donut | Commercial snack doughnuts | Danish, breakfast pastries, doughnuts, granola bars |
| desserts - miscellaneous, snacks - commercial packaged, brand name listing, Hostess, Hostess Suzy Q's | Commercial snack cakes and muffins | Cakes |
| desserts - miscellaneous, snacks - commercial packaged, brand name listing, Dolly Madison, Dolly Madison Buttercrumb Cake | Commercial snack cakes and muffins | Coffee cakes, not yeast |
| desserts - miscellaneous, snacks - commercial packaged, brand name listing, Dolly Madison, Dolly Madison Cinnamon Swirl Coffee Cake | Commercial snack sweet rolls and coffee cakes | Coffee cakes, not yeast |
| desserts - miscellaneous, snacks - commercial packaged, brand name listing, Dolly Madison, Dolly Madison Apple Crumb Cake | Commercial snack cakes and muffins | Coffee cakes, not yeast |
| desserts - miscellaneous, snacks - commercial packaged, brand name listing, Dolly Madison, Dolly Madison Carrot Cake | Commercial snack cakes and muffins | Cakes |
| desserts - miscellaneous, snacks - commercial packaged, brand name listing, Dolly Madison, Dolly Madison Bear Claw | Commercial snack sweet rolls and coffee cakes | Danish, breakfast pastries, doughnuts |
| desserts - miscellaneous, snacks - commercial packaged, brand name listing, Dolly Madison, Dolly Madison Chocolate Cupcake | Commercial snack cakes and muffins | Cakes |
| desserts - miscellaneous, snacks - commercial packaged, brand name listing, Dolly Madison, Dolly Madison Cream Cheese Danish | Commercial snack sweet rolls and coffee cakes | Danish, breakfast pastries, doughnuts, granola bars |
| desserts - miscellaneous, snacks - commercial packaged, brand name listing, Dolly Madison, Dolly Madison Donut Gems - Cinnamon Sugar | Commercial snack doughnuts | Danish, breakfast pastries, doughnuts, granola bars |
| desserts - miscellaneous, snacks - commercial packaged, brand name listing, Dolly Madison, Dolly Madison Donut Gems - Chocolate Coated | Commercial snack doughnuts | Danish, breakfast pastries, doughnuts, granola bars |
| desserts - miscellaneous, snacks - commercial packaged, brand name listing, Dolly Madison, Dolly Madison Donut Gems - Powder Sugar | Commercial snack doughnuts | Danish, breakfast pastries, doughnuts, granola bars |
| desserts - miscellaneous, snacks - commercial packaged, brand name listing, Dolly Madison, Dolly Madison Angel Food Bar - mini | Commercial snack cakes and muffins | Cakes |
| desserts - miscellaneous, snacks - commercial packaged, brand name listing, Dolly Madison, Dolly Madison Honey Bun | Commercial snack sweet rolls and coffee cakes | White breads, rolls |
| desserts - miscellaneous, snacks - commercial packaged, brand name listing, Dolly Madison, Dolly Madison Pecan Rollers | Commercial snack sweet rolls and coffee cakes | Danish, breakfast pastries, doughnuts, granola bars |
| desserts - miscellaneous, snacks - commercial packaged, brand name listing, Dolly Madison, Dolly Madison Pie - Apple | Commercial snack pies | Pies |
| desserts - miscellaneous, snacks - commercial packaged, brand name listing, Dolly Madison, Dolly Madison Pie - Blueberry | Commercial snack pies | Pies |
| desserts - miscellaneous, snacks - commercial packaged, brand name listing, Dolly Madison, Dolly Madison Mini Pound Cake | Commercial snack cakes and muffins | Cakes |
| desserts - miscellaneous, snacks - commercial packaged, brand name listing, Dolly Madison, Dolly Madison Sweet Roll - Apple | Commercial snack sweet rolls and coffee cakes | White breads, rolls |
| desserts - miscellaneous, snacks - commercial packaged, brand name listing, Dolly Madison, Dolly Madison Koo Koo's | Commercial snack cakes and muffins | Cakes |
| desserts - miscellaneous, snacks - commercial packaged, brand name listing, Dolly Madison, Dolly Madison Zingers - Iced Devil's Food | Commercial snack cakes and muffins | Cakes |
| desserts - miscellaneous, snacks - commercial packaged, brand name listing, Dolly Madison, Dolly Madison Creme Cakes | Commercial snack cakes and muffins | Cakes |
| desserts - miscellaneous, snacks - commercial packaged, brand name listing, Dolly Madison, Dolly Madison Zingers - Raspberry | Commercial snack cakes and muffins | Cakes |
| desserts - miscellaneous, snacks - commercial packaged, brand name listing, Tastykake, Tastykake Cupcake - Buttercremes | Commercial snack cakes and muffins | Cakes |
| desserts - miscellaneous, snacks - commercial packaged, brand name listing, Tastykake, Tastykake Chocolate Kreamies | Commercial snack cakes and muffins | Cakes |
| desserts - miscellaneous, snacks - commercial packaged, brand name listing, Drake's, Drake's Ring Dings | Commercial snack cakes and muffins | Cakes |
| desserts - miscellaneous, snacks - commercial packaged, cupcake (commercially packaged), regular | Commercial snack cakes and muffins | Cakes |
| desserts - miscellaneous, snacks - commercial packaged, fruit pie or turnover | Commercial snack pies | Pies |
| desserts - miscellaneous, snacks - commercial packaged, brand name listing, Hostess, Hostess Cupcake - Chocolate low fat | Commercial snack cakes and muffins | Cakes |
| ice cream and frozen desserts, treats (frozen prepackaged), brand name listing, Buster Bar | Frozen treats | Milk desserts, frozen |
| ice cream and frozen desserts, treats (frozen prepackaged), ice milk bar (chocolate coated), with nuts | Frozen treats | Milk desserts, frozen |
| ice cream and frozen desserts, treats (frozen prepackaged), fruit juice bar, no sugar added (fortified with vitamin C) | Frozen treats | Mixtures of fruits and nonfruit items |
| ice cream and frozen desserts, treats (frozen prepackaged), Popsicle, sugar free | Frozen treats | Ices or popsicles |
| desserts - miscellaneous, snacks - commercial packaged, brand name listing, Weight Watchers, Weight Watchers Brownie Cheesecake | Commercial snack cakes and muffins | Cakes |
| desserts - miscellaneous, snacks - commercial packaged, brand name listing, Weight Watchers, Weight Watchers Chocolate Frosted Brownie | Cookies | Cookies |
| desserts - miscellaneous, snacks - commercial packaged, brand name listing, Weight Watchers, Weight Watchers Chocolate Mocha Pie | Commercial snack pies | Pies |
| desserts - miscellaneous, snacks - commercial packaged, brand name listing, Weight Watchers, Weight Watchers Smart Ones Chocolate Mousse | Commercial snack - miscellaneous desserts | Puddings, custards, and other milk desserts |
| desserts - miscellaneous, snacks - commercial packaged, brand name listing, Weight Watchers, Weight Watchers Praline Pecan Mousse | Commercial snack - miscellaneous desserts | Puddings, custards, and other milk desserts |
| desserts - miscellaneous, snacks - commercial packaged, brand name listing, Entenmann's, Entenmann's Cherry Filled Coffee Cake - fat free | Commercial snack sweet rolls and coffee cakes | Coffee cakes, not yeast |
| desserts - miscellaneous, snacks - commercial packaged, brand name listing, Entenmann's, Entenmann's Cinnamon Apple Coffee Cake - fat free | Commercial snack sweet rolls and coffee cakes | Coffee cakes, not yeast |
| desserts - miscellaneous, snacks - commercial packaged, brand name listing, Entenmann's, Entenmann's Banana Crunch Cake - fat free | Commercial snack cakes and muffins | Coffee cakes, not yeast |
| desserts - miscellaneous, snacks - commercial packaged, brand name listing, Entenmann's, Entenmann's Blueberry Crunch Cake - fat free | Commercial snack cakes and muffins | Coffee cakes, not yeast |
| desserts - miscellaneous, snacks - commercial packaged, brand name listing, Entenmann's, Entenmann's Pineapple Crunch Cake - fat free | Commercial snack cakes and muffins | Coffee cakes, not yeast |
| desserts - miscellaneous, snacks - commercial packaged, brand name listing, Entenmann's, Entenmann's Golden Loaf Cake - fat free | Commercial snack cakes and muffins | Cakes |
| desserts - miscellaneous, snacks - commercial packaged, brand name listing, Entenmann's, Entenmann's Cherry Cheese Pastry - fat free | Commercial snack sweet rolls and coffee cakes | Danish, breakfast pastries, doughnuts, granola bars |
| desserts - miscellaneous, snacks - commercial packaged, brand name listing, Entenmann's, Entenmann's Pineapple Cheese Pastry - fat free | Commercial snack sweet rolls and coffee cakes | Danish, breakfast pastries, doughnuts, granola bars |
| desserts - miscellaneous, snacks - commercial packaged, brand name listing, Entenmann's, Entenmann's Raspberry Twist - fat free | Commercial snack sweet rolls and coffee cakes | Danish, breakfast pastries, doughnuts, granola bars |
| desserts - miscellaneous, snacks - commercial packaged, brand name listing, Hostess, Hostess Muffin - Mini, Blueberry | Commercial snack cakes and muffins | Other muffins, popovers |
| desserts - miscellaneous, snacks - commercial packaged, brand name listing, Little Debbie, Little Debbie Coconut Creme Cake | Commercial snack cakes and muffins | Cakes |
| desserts - miscellaneous, snacks - commercial packaged, brand name listing, Little Debbie, Little Debbie Devil Cremes | Commercial snack cakes and muffins | Cakes |
| desserts - miscellaneous, snacks - commercial packaged, brand name listing, Little Debbie, Little Debbie Fancy Cakes | Commercial snack cakes and muffins | Cakes |
| desserts - miscellaneous, snacks - commercial packaged, brand name listing, Little Debbie, Little Debbie Strawberry Shortcake Rolls | Commercial snack cakes and muffins | Cakes |
| desserts - miscellaneous, snacks - commercial packaged, brand name listing, Little Debbie, Little Debbie Peanut Clusters | Commercial snack cakes and muffins | Cakes |
| desserts - miscellaneous, snacks - commercial packaged, brand name listing, Little Debbie, Little Debbie Dessert Cakes - Spice | Commercial snack cakes and muffins | Cakes |
| desserts - miscellaneous, snacks - commercial packaged, brand name listing, Little Debbie, Little Debbie Dessert Cakes - Chocolate | Commercial snack cakes and muffins | Cakes |
| desserts - miscellaneous, snacks - commercial packaged, brand name listing, Little Debbie, Little Debbie Chocolate Chip Cakes | Commercial snack cakes and muffins | Cakes |
| desserts - miscellaneous, snacks - commercial packaged, brand name listing, Little Debbie, Little Debbie Swiss Cake Rolls | Commercial snack cakes and muffins | Cakes |
| ice cream and frozen desserts, treats (frozen prepackaged), brand name listing, Wells' Blue Bunny, Blue Bunny Sweet Freedom Fruit Juice Bar - all flavors | Frozen treats | Mixtures of fruits and nonfruit items |
| ice cream and frozen desserts, treats (frozen prepackaged), brand name listing, Wells' Blue Bunny, Blue Bunny Sweet Freedom Ice Cream Lites | Frozen treats | Milk desserts, frozen |
| ice cream and frozen desserts, treats (frozen prepackaged), brand name listing, Nestle, Nestle Cool Creations - Mickey Mouse Bar | Frozen treats | Milk desserts, frozen |
| desserts - miscellaneous, snacks - commercial packaged, brand name listing, Pepperidge Farm, Pepperidge Farm Dumpling - Apple | Commercial snack - miscellaneous desserts | Cobblers, eclairs, turnovers, other pastries |
| desserts - miscellaneous, snacks - commercial packaged, brand name listing, Pepperidge Farm, Pepperidge Farm Turnover - Apple | Commercial snack - miscellaneous desserts | Cobblers, eclairs, turnovers, other pastries |
| desserts - miscellaneous, snacks - commercial packaged, brand name listing, Pepperidge Farm, Pepperidge Farm Turnover - Blueberry | Commercial snack - miscellaneous desserts | Cobblers, eclairs, turnovers, other pastries |
| desserts - miscellaneous, snacks - commercial packaged, brand name listing, Pepperidge Farm, Pepperidge Farm Turnover - Cherry | Commercial snack - miscellaneous desserts | Cobblers, eclairs, turnovers, other pastries |
| desserts - miscellaneous, snacks - commercial packaged, brand name listing, Pepperidge Farm, Pepperidge Farm Turnover - Peach | Commercial snack - miscellaneous desserts | Cobblers, eclairs, turnovers, other pastries |
| desserts - miscellaneous, snacks - commercial packaged, brand name listing, Pepperidge Farm, Pepperidge Farm Turnover - Raspberry | Commercial snack - miscellaneous desserts | Cobblers, eclairs, turnovers, other pastries |
| desserts - miscellaneous, snacks - commercial packaged, brand name listing, Sara Lee, Sara Lee Pound Cake - snack size | Commercial snack cakes and muffins | Cakes |
| desserts - miscellaneous, snacks - commercial packaged, brand name listing, Sara Lee, Sara Lee Free & Light Pound Cake | Commercial snack cakes and muffins | Cakes |
| desserts - miscellaneous, snacks - commercial packaged, brand name listing, Sara Lee, Sara Lee Strawberry French Cheesecake | Commercial snack cakes and muffins | Cakes |
| desserts - miscellaneous, snacks - commercial packaged, brand name listing, Tastykake, Tastykake Donuts - Rich Frosted | Commercial snack doughnuts | Danish, breakfast pastries, doughnuts, granola bars |
| desserts - miscellaneous, snacks - commercial packaged, brand name listing, Tastykake, Tastykake Honeybun - Glazed | Commercial snack sweet rolls and coffee cakes | White breads, rolls |
| desserts - miscellaneous, snacks - commercial packaged, brand name listing, Tastykake, Tastykake Honeybun - Iced | Commercial snack sweet rolls and coffee cakes | White breads, rolls |
| desserts - miscellaneous, snacks - commercial packaged, brand name listing, Tastykake, Tastykake Kandy Kake - Coconut | Commercial snack cakes and muffins | Cakes |
| desserts - miscellaneous, snacks - commercial packaged, brand name listing, Tastykake, Tastykake Kandy Kake - Peanut Butter | Commercial snack cakes and muffins | Cakes |
| desserts - miscellaneous, snacks - commercial packaged, brand name listing, Tastykake, Tastykake Krimpet - Strawberry | Commercial snack cakes and muffins | Cakes |
| desserts - miscellaneous, snacks - commercial packaged, brand name listing, Tastykake, Tastykake Pecan Swirls | Commercial snack sweet rolls and coffee cakes | Danish, breakfast pastries, doughnuts |
| desserts - miscellaneous, snacks - commercial packaged, brand name listing, Tastykake, Tastykake Pie - French Apple | Commercial snack pies | Pies |
| desserts - miscellaneous, snacks - commercial packaged, brand name listing, Tastykake, Tastykake Pie - Pineapple Cheese | Commercial snack pies | Pies |
| desserts - miscellaneous, snacks - commercial packaged, brand name listing, Tastykake, Tastykake Pie - Pumpkin | Commercial snack pies | Pies |
| ice cream and frozen desserts, treats (frozen prepackaged), ice milk sandwich | Frozen treats | Milk desserts, frozen |
| ice cream and frozen desserts, treats (frozen prepackaged), brand name listing, Good Humor, Good Humor Klondike - original | Frozen treats | Milk desserts, frozen |
| ice cream and frozen desserts, treats (frozen prepackaged), brand name listing, Dove, Dove Bite Size - all flavors | Frozen treats | Milk desserts, frozen |
| ice cream and frozen desserts, treats (frozen prepackaged), brand name listing, M & M/Mars, Snickers Ice Cream Bar | Frozen treats | Milk desserts, frozen |
| desserts - miscellaneous, snacks - commercial packaged, brand name listing, Tastykake, Tastykake Pie - Tasty Klair | Commercial snack pies | Pies |
| ice cream and frozen desserts, treats (frozen prepackaged), ice cream bar, no sugar added | Frozen treats | Milk desserts, frozen |
| ice cream and frozen desserts, treats (frozen prepackaged), brand name listing, M & M/Mars, 3 Musketeers Ice Cream Bars - all flavors | Frozen treats | Milk desserts, frozen |
| ice cream and frozen desserts, treats (frozen prepackaged), brand name listing, Baskin-Robbins, Baskin-Robbins Tiny Toon Bar - mint chocolate chip | Frozen treats | Milk desserts, frozen |
| ice cream and frozen desserts, treats (frozen prepackaged), brand name listing, Baskin-Robbins, Baskin-Robbins Tiny Toon Bar - vanilla | Frozen treats | Milk desserts, frozen |
| ice cream and frozen desserts, treats (frozen prepackaged), brand name listing, Ben & Jerry's, Ben & Jerry's Cherry Garcia Yogurt Peace Pop | Frozen treats | Milk desserts, frozen |
| ice cream and frozen desserts, treats (frozen prepackaged), brand name listing, Ben & Jerry's, Ben & Jerry's Chocolate Cookie Dough Peace Pop | Frozen treats | Milk desserts, frozen |
| ice cream and frozen desserts, treats (frozen prepackaged), brand name listing, Ben & Jerry's, Ben & Jerry's Vanilla with Heath Coffee Crunch Peace Pop | Frozen treats | Milk desserts, frozen |
| ice cream and frozen desserts, treats (frozen prepackaged), brand name listing, Blue Bell, Blue Bell 3-D Funshape Bar | Frozen treats | Ices and popsicles |
| ice cream and frozen desserts, treats (frozen prepackaged), brand name listing, Blue Bell, Blue Bell Banana Fudge Bar | Frozen treats | Milk desserts, frozen |
| ice cream and frozen desserts, treats (frozen prepackaged), brand name listing, Blue Bell, Blue Bell Big Red | Frozen treats | Ices and popsicles |
| ice cream and frozen desserts, treats (frozen prepackaged), brand name listing, Blue Bell, Blue Bell Big Shot | Frozen treats | Ices and popsicles |
| ice cream and frozen desserts, treats (frozen prepackaged), brand name listing, Blue Bell, Blue Bell Bullet - cherry | Frozen treats | Ices and popsicles |
| ice cream and frozen desserts, treats (frozen prepackaged), brand name listing, Blue Bell, Blue Bell Bullet - coconut | Frozen treats | Ices and popsicles |
| ice cream and frozen desserts, treats (frozen prepackaged), brand name listing, Blue Bell, Blue Bell Bullet - sugar free | Frozen treats | Ices and popsicles |
| ice cream and frozen desserts, treats (frozen prepackaged), brand name listing, Blue Bell, Blue Bell Cherry Cream Bar | Frozen treats | Milk desserts, frozen |
| ice cream and frozen desserts, treats (frozen prepackaged), brand name listing, Blue Bell, Blue Bell Cherry Freeze Bar | Frozen treats | Ices and popsicles |
| ice cream and frozen desserts, treats (frozen prepackaged), brand name listing, Blue Bell, Blue Bell Chocolate Fudge Bar | Frozen treats | Milk desserts, frozen |
| ice cream and frozen desserts, treats (frozen prepackaged), brand name listing, Blue Bell, Blue Bell Cookies 'N Cream Sandwich | Frozen treats | Milk desserts, frozen |
| ice cream and frozen desserts, treats (frozen prepackaged), brand name listing, Blue Bell, Blue Bell Creme Pop | Frozen treats | Milk desserts, frozen |
| ice cream and frozen desserts, treats (frozen prepackaged), brand name listing, Blue Bell, Blue Bell Double Fudge Bar | Frozen treats | Milk desserts, frozen |
| ice cream and frozen desserts, treats (frozen prepackaged), brand name listing, Blue Bell, Blue Bell Dream Bar | Frozen treats | Milk desserts, frozen |
| ice cream and frozen desserts, treats (frozen prepackaged), brand name listing, Blue Bell, Blue Bell Frostbite | Frozen treats | Ices and popsicles |
| ice cream and frozen desserts, treats (frozen prepackaged), brand name listing, Blue Bell, Blue Bell Fudge Bar - diet | Frozen treats | Milk desserts, frozen |
| ice cream and frozen desserts, treats (frozen prepackaged), brand name listing, Blue Bell, Blue Bell Fudge Bar - fat free | Frozen treats | Milk desserts, frozen |
| ice cream and frozen desserts, treats (frozen prepackaged), brand name listing, Blue Bell, Blue Bell Grizzly Bar | Frozen treats | Milk desserts, frozen |
| ice cream and frozen desserts, treats (frozen prepackaged), brand name listing, Blue Bell, Blue Bell Homemade Vanilla Bar | Frozen treats | Milk desserts, frozen |
| ice cream and frozen desserts, treats (frozen prepackaged), brand name listing, Blue Bell, Blue Bell Ice Cream Cup - homemade vanilla | Ice cream, ice milk, sherbet, nondairy frozen dessert, and milkshakes | Milk desserts, frozen |
| ice cream and frozen desserts, treats (frozen prepackaged), brand name listing, Blue Bell, Blue Bell Mini Sandwich | Frozen treats | Milk desserts, frozen |
| ice cream and frozen desserts, treats (frozen prepackaged), brand name listing, Blue Bell, Blue Bell Mini Sandwich - light | Frozen treats | Milk desserts, frozen |
| ice cream and frozen desserts, treats (frozen prepackaged), brand name listing, Blue Bell, Blue Bell Mooo Bar | Frozen treats | Milk desserts, frozen |
| ice cream and frozen desserts, treats (frozen prepackaged), brand name listing, Blue Bell, Blue Bell Mooo Bar - diet | Frozen treats | Milk desserts, frozen |
| ice cream and frozen desserts, treats (frozen prepackaged), brand name listing, Blue Bell, Blue Bell Neapolitan Sandwich | Frozen treats | Milk desserts, frozen |
| ice cream and frozen desserts, treats (frozen prepackaged), brand name listing, Blue Bell, Blue Bell Nutzo | Frozen treats | Milk desserts, frozen |
| ice cream and frozen desserts, treats (frozen prepackaged), brand name listing, Blue Bell, Blue Bell Rainbow Freeze | Frozen treats | Ices and popsicles |
| ice cream and frozen desserts, treats (frozen prepackaged), brand name listing, Blue Bell, Blue Bell Rainbow Fruiti Freeze | Frozen treats | Ices and popsicles |
| ice cream and frozen desserts, treats (frozen prepackaged), brand name listing, Blue Bell, Blue Bell Ice Cream Sandwich | Frozen treats | Milk desserts, frozen |
| ice cream and frozen desserts, treats (frozen prepackaged), brand name listing, Blue Bell, Blue Bell Twin Pop | Frozen treats | Ices and popsicles |
| ice cream and frozen desserts, treats (frozen prepackaged), brand name listing, Borden, Borden Chocolate Coated Ice Cream Bar | Frozen treats | Milk desserts, frozen |
| ice cream and frozen desserts, treats (frozen prepackaged), brand name listing, Borden, Borden Chocolate Coated Ice Cream Bar - lowfat | Frozen treats | Milk desserts, frozen |
| ice cream and frozen desserts, treats (frozen prepackaged), brand name listing, Borden, Borden Fudge Bars - all flavors | Frozen treats | Milk desserts, frozen |
| ice cream and frozen desserts, treats (frozen prepackaged), brand name listing, Borden, Borden Cracker Jack Butter Toffee Ice Cream Bar | Frozen treats | Milk desserts, frozen |
| ice cream and frozen desserts, treats (frozen prepackaged), brand name listing, Borden, Borden Cracker Jack Original Ice Cream Bar | Frozen treats | Milk desserts, frozen |
| ice cream and frozen desserts, treats (frozen prepackaged), brand name listing, Borden, Borden Frosty Dream Bar | Frozen treats | Milk desserts, frozen |
| ice cream and frozen desserts, treats (frozen prepackaged), brand name listing, Borden, Borden Ice Cream Sandwich - Mississippi mud pie | Frozen treats | Milk desserts, frozen |
| ice cream and frozen desserts, treats (frozen prepackaged), brand name listing, Borden, Borden Ice Cream Sandwich - neapolitan | Frozen treats | Milk desserts, frozen |
| ice cream and frozen desserts, treats (frozen prepackaged), brand name listing, Borden, Borden Ice Cream Sandwich - vanilla | Frozen treats | Milk desserts, frozen |
| ice cream and frozen desserts, treats (frozen prepackaged), brand name listing, Borden, Borden Juice Sticks | Frozen treats | Mixtures of fruits and nonfruit items |
| ice cream and frozen desserts, treats (frozen prepackaged), brand name listing, Borden, Borden Nutty Buddy Sundae Cone - vanilla ice cream | Frozen treats | Milk desserts, frozen |
| ice cream and frozen desserts, treats (frozen prepackaged), brand name listing, Borden, Borden Nutty Buddy Sundae Cone - vanilla ice milk | Frozen treats | Milk desserts, frozen |
| ice cream and frozen desserts, treats (frozen prepackaged), brand name listing, Borden, Borden Nutty Buddy Sundae Cone - vanilla mellorine | Frozen treats | Milk desserts, frozen |
| ice cream and frozen desserts, treats (frozen prepackaged), brand name listing, Borden, Borden Orange Flavored Dream Bar | Frozen treats | Milk desserts, frozen |
| ice cream and frozen desserts, treats (frozen prepackaged), brand name listing, Borden, Borden Orange Flavored Dream Bar - junior | Frozen treats | Milk desserts, frozen |
| ice cream and frozen desserts, treats (frozen prepackaged), brand name listing, Borden, Borden Pops Juniors | Frozen treats | Ices and popsicles |
| ice cream and frozen desserts, treats (frozen prepackaged), brand name listing, Nestle, Butterfinger Ice Cream Bar | Frozen treats | Milk desserts, frozen |
| ice cream and frozen desserts, treats (frozen prepackaged), brand name listing, Dole, Dole Fruit Juice Bar - all flavors | Frozen treats | Mixtures of fruits and nonfruit items |
| ice cream and frozen desserts, treats (frozen prepackaged), brand name listing, Dove, Dove Bar - almond | Frozen treats | Milk desserts, frozen |
| ice cream and frozen desserts, treats (frozen prepackaged), brand name listing, Dove, Dove Bar - caramel creme swirl with toffee chips | Frozen treats | Milk desserts, frozen |
| ice cream and frozen desserts, treats (frozen prepackaged), brand name listing, Dove, Dove Bar - chocolate with dark chocolate | Frozen treats | Milk desserts, frozen |
| ice cream and frozen desserts, treats (frozen prepackaged), brand name listing, Dove, Dove Bar - mocha cashew crunch | Frozen treats | Milk desserts, frozen |
| ice cream and frozen desserts, treats (frozen prepackaged), brand name listing, Dove, Dove Bar - vanilla with dark chocolate | Frozen treats | Milk desserts, frozen |
| ice cream and frozen desserts, treats (frozen prepackaged), brand name listing, Dove, Dove Bar - vanilla with milk chocolate | Frozen treats | Milk desserts, frozen |
| ice cream and frozen desserts, treats (frozen prepackaged), brand name listing, Nestle, Flintstones Cool Cream | Frozen treats | Milk desserts, frozen |
| ice cream and frozen desserts, treats (frozen prepackaged), brand name listing, Nestle, Flintstones Push-Up - all flavors | Frozen treats | Milk desserts, frozen |
| ice cream and frozen desserts, treats (frozen prepackaged), brand name listing, Haagen-Dazs, Haagen-Dazs Ice Cream Bar - coffee & almond crunch | Frozen treats | Milk desserts, frozen |
| ice cream and frozen desserts, treats (frozen prepackaged), brand name listing, Haagen-Dazs, Haagen-Dazs Ice Cream Bar - vanilla & almonds | Frozen treats | Milk desserts, frozen |
| ice cream and frozen desserts, treats (frozen prepackaged), brand name listing, Haagen-Dazs, Haagen-Dazs Ice Cream Bar - vanilla & dark chocolate | Frozen treats | Milk desserts, frozen |
| ice cream and frozen desserts, treats (frozen prepackaged), brand name listing, Haagen-Dazs, Haagen-Dazs Yogurt Bar - cherry chocolate fudge | Frozen treats | Milk desserts, frozen |
| ice cream and frozen desserts, treats (frozen prepackaged), brand name listing, Haagen-Dazs, Haagen-Dazs Yogurt Bar - peach | Frozen treats | Milk desserts, frozen |
| ice cream and frozen desserts, treats (frozen prepackaged), brand name listing, Haagen-Dazs, Haagen-Dazs Yogurt Bar - pina colada | Frozen treats | Milk desserts, frozen |
| ice cream and frozen desserts, treats (frozen prepackaged), brand name listing, Haagen-Dazs, Haagen-Dazs Yogurt Bar - raspberry & vanilla | Frozen treats | Milk desserts, frozen |
| ice cream and frozen desserts, treats (frozen prepackaged), brand name listing, Haagen-Dazs, Haagen-Dazs Yogurt Bar - strawberry daiquiri | Frozen treats | Milk desserts, frozen |
| ice cream and frozen desserts, treats (frozen prepackaged), brand name listing, Haagen-Dazs, Haagen-Dazs Yogurt Bar - tropical orange passion | Frozen treats | Milk desserts, frozen |
| ice cream and frozen desserts, treats (frozen prepackaged), brand name listing, Kemps, Kemps All American Pop | Frozen treats | Ices and popsicles |
| ice cream and frozen desserts, treats (frozen prepackaged), brand name listing, Kemps, Kemps Float Bar | Frozen treats | Milk desserts, frozen |
| ice cream and frozen desserts, treats (frozen prepackaged), brand name listing, Kemps, Kemps Frosty Santa | Frozen treats | Milk desserts, frozen |
| ice cream and frozen desserts, treats (frozen prepackaged), brand name listing, Kemps, Kemps Frosty Tree | Frozen treats | Milk desserts, frozen |
| ice cream and frozen desserts, treats (frozen prepackaged), brand name listing, Kemps, Kemps Fudge Bar | Frozen treats | Milk desserts, frozen |
| ice cream and frozen desserts, treats (frozen prepackaged), brand name listing, Kemps, Kemps Fudge Jr.'s | Frozen treats | Milk desserts, frozen |
| ice cream and frozen desserts, treats (frozen prepackaged), brand name listing, Kemps, Kemps Ice Cream Bar | Frozen treats | Milk desserts, frozen |
| ice cream and frozen desserts, treats (frozen prepackaged), brand name listing, Kemps, Kemps Ice Cream Cup Jr.'s - chocolate | Ice cream, ice milk, sherbet, nondairy frozen dessert, and milkshakes | Milk desserts, frozen |
| ice cream and frozen desserts, treats (frozen prepackaged), brand name listing, Kemps, Kemps Ice Cream Cup Jr.'s - vanilla | Ice cream, ice milk, sherbet, nondairy frozen dessert, and milkshakes | Milk desserts, frozen |
| ice cream and frozen desserts, treats (frozen prepackaged), brand name listing, Kemps, Kemps Ice Cream Sandwich | Frozen treats | Milk desserts, frozen |
| ice cream and frozen desserts, treats (frozen prepackaged), brand name listing, Kemps, Kemps Juice Kooler | Frozen treats | Mixtures of fruits and nonfruit items |
| ice cream and frozen desserts, treats (frozen prepackaged), brand name listing, Kemps, Kemps Krunch Bar | Frozen treats | Milk desserts, frozen |
| ice cream and frozen desserts, treats (frozen prepackaged), brand name listing, Kemps, Kemps Winter Snowmen | Frozen treats | Milk desserts, frozen |
| ice cream and frozen desserts, treats (frozen prepackaged), brand name listing, Kemps, Kemps Orange Cream Bar | Frozen treats | Milk desserts, frozen |
| ice cream and frozen desserts, treats (frozen prepackaged), brand name listing, Kemps, Kemps Pop Jr.'s | Frozen treats | Ices and popsicles |
| ice cream and frozen desserts, treats (frozen prepackaged), brand name listing, Kemps, Kemps Toffee Bar | Frozen treats | Milk desserts, frozen |
| ice cream and frozen desserts, treats (frozen prepackaged), brand name listing, Kemps, Kemps Twin Pops | Frozen treats | Ices and popsicles |
| ice cream and frozen desserts, treats (frozen prepackaged), brand name listing, Wells' Blue Bunny, Looney Surprise - berry cherry | Frozen treats | Ices and popsicles |
| ice cream and frozen desserts, treats (frozen prepackaged), brand name listing, Wells' Blue Bunny, Looney Tunes Bugs Bunny Bar | Frozen treats | Ices and popsicles |
| ice cream and frozen desserts, treats (frozen prepackaged), brand name listing, Wells' Blue Bunny, Looney Tunes Tweety Bird Face Bar | Frozen treats | Ices and popsicles |
| ice cream and frozen desserts, treats (frozen prepackaged), brand name listing, M & M/Mars, Milky Way Ice Cream Bars - all flavors | Frozen treats | Milk desserts, frozen |
| ice cream and frozen desserts, treats (frozen prepackaged), brand name listing, Natural Nectar, Natural Nectar Cream Freeze Bar - cocoa fudge-n-cream | Frozen treats | Milk desserts, frozen |
| ice cream and frozen desserts, treats (frozen prepackaged), brand name listing, Natural Nectar, Natural Nectar Cream Freeze Bar - wildberry fruit-n-cream | Frozen treats | Milk desserts, frozen |
| ice cream and frozen desserts, treats (frozen prepackaged), brand name listing, Natural Nectar, Natural Nectar 100% Juice Bar | Frozen treats | Mixtures of fruits and nonfruit items |
| ice cream and frozen desserts, treats (frozen prepackaged), brand name listing, Natural Nectar, Natural Nectar Pie - mocha | Frozen treats | Milk desserts, frozen |
| ice cream and frozen desserts, treats (frozen prepackaged), brand name listing, Natural Nectar, Natural Nectar Pie - nectar | Frozen treats | Milk desserts, frozen |
| ice cream and frozen desserts, treats (frozen prepackaged), brand name listing, Nestle, Nestle Bon Bons | Frozen treats | Milk desserts, frozen |
| ice cream and frozen desserts, treats (frozen prepackaged), brand name listing, Nestle, Nestle Crunch Ice Cream Bar - chocolate | Frozen treats | Milk desserts, frozen |
| ice cream and frozen desserts, treats (frozen prepackaged), brand name listing, Nestle, Nestle Crunch Ice Cream Bar - vanilla | Frozen treats | Milk desserts, frozen |
| ice cream and frozen desserts, treats (frozen prepackaged), brand name listing, Nestle, Nestle Drumstick - chocolate | Frozen treats | Milk desserts, frozen |
| ice cream and frozen desserts, treats (frozen prepackaged), brand name listing, Nestle, Nestle Drumstick - vanilla | Frozen treats | Milk desserts, frozen |
| ice cream and frozen desserts, treats (frozen prepackaged), brand name listing, Nestle, Nestle Drumstick - vanilla fudge | Frozen treats | Milk desserts, frozen |
| ice cream and frozen desserts, treats (frozen prepackaged), brand name listing, Schwan's, Schwan's Ice Cream Bar | Frozen treats | Milk desserts, frozen |
| ice cream and frozen desserts, treats (frozen prepackaged), brand name listing, Schwan's, Schwan's Chocolate Pudding Bar | Frozen treats | Milk desserts, frozen |
| ice cream and frozen desserts, treats (frozen prepackaged), brand name listing, Schwan's, Schwan's Sundae Crunch Bar | Frozen treats | Milk desserts, frozen |
| ice cream and frozen desserts, treats (frozen prepackaged), brand name listing, Schwan's, Schwan's English Toffee Bar | Frozen treats | Milk desserts, frozen |
| ice cream and frozen desserts, treats (frozen prepackaged), brand name listing, Schwan's, Schwan's Fudge Stick | Frozen treats | Milk desserts, frozen |
| ice cream and frozen desserts, treats (frozen prepackaged), brand name listing, Schwan's, Schwan's Gold 'N Nugit Bar | Frozen treats | Milk desserts, frozen |
| ice cream and frozen desserts, treats (frozen prepackaged), brand name listing, Schwan's, Schwan's Ice Cream Sandwich | Frozen treats | Milk desserts, frozen |
| ice cream and frozen desserts, treats (frozen prepackaged), brand name listing, Schwan's, Schwan's Peanut Stick | Frozen treats | Milk desserts, frozen |
| ice cream and frozen desserts, treats (frozen prepackaged), brand name listing, Schwan's, Schwan's Pop | Frozen treats | Ices and popsicles |
| ice cream and frozen desserts, treats (frozen prepackaged), brand name listing, Schwan's, Schwan's Push-Em - chocolate malt | Frozen treats | Milk desserts, frozen |
| ice cream and frozen desserts, treats (frozen prepackaged), brand name listing, Schwan's, Schwan's Push-Em - sherbet | Frozen treats | Milk desserts, frozen |
| ice cream and frozen desserts, treats (frozen prepackaged), brand name listing, Schwan's, Schwan's Push-Em - strawberry shake | Frozen treats | Milk desserts, frozen |
| ice cream and frozen desserts, treats (frozen prepackaged), brand name listing, Schwan's, Schwan's Push-Em - yogurt, all flavors | Frozen treats | Milk desserts, frozen |
| ice cream and frozen desserts, treats (frozen prepackaged), brand name listing, Schwan's, Schwan's Rainbow Stick | Frozen treats | Milk desserts, frozen |
| ice cream and frozen desserts, treats (frozen prepackaged), brand name listing, Schwan's, Schwan's Root Beer Float Bar | Frozen treats | Milk desserts, frozen |
| ice cream and frozen desserts, treats (frozen prepackaged), brand name listing, Schwan's, Schwan's Silver Mint Bar | Frozen treats | Milk desserts, frozen |
| ice cream and frozen desserts, treats (frozen prepackaged), brand name listing, Schwan's, Schwan's Strawberry Fruit Bar | Frozen treats | Mixtures of fruits and nonfruit items |
| ice cream and frozen desserts, treats (frozen prepackaged), brand name listing, Schwan's, Schwan's Sundae Cone | Frozen treats | Milk desserts, frozen |
| ice cream and frozen desserts, treats (frozen prepackaged), brand name listing, Schwan's, Schwan's Sundae Cone Pecan Praline | Frozen treats | Milk desserts, frozen |
| ice cream and frozen desserts, treats (frozen prepackaged), brand name listing, Schwan's, Schwan's Sundae Cup - chocolate | Ice cream, ice milk, sherbet, nondairy frozen dessert, and milkshakes | Milk desserts, frozen |
| ice cream and frozen desserts, treats (frozen prepackaged), brand name listing, Schwan's, Schwan's Sundae Cup - strawberry | Ice cream, ice milk, sherbet, nondairy frozen dessert, and milkshakes | Milk desserts, frozen |
| ice cream and frozen desserts, treats (frozen prepackaged), brand name listing, Schwan's, Schwan's Trim Creations Chocolate Fudge Stick | Frozen treats | Milk desserts, frozen |
| ice cream and frozen desserts, treats (frozen prepackaged), brand name listing, Schwan's, Schwan's Twin Pops | Frozen treats | Ices and popsicles |
| ice cream and frozen desserts, treats (frozen prepackaged), brand name listing, Schwan's, Schwan's Ice Cream Cup - vanilla | Ice cream, ice milk, sherbet, nondairy frozen dessert, and milkshakes | Milk desserts, frozen |
| ice cream and frozen desserts, treats (frozen prepackaged), brand name listing, TCBY, TCBY Yog-A-Bar - raspberry swirl | Frozen treats | Milk desserts, frozen |
| ice cream and frozen desserts, treats (frozen prepackaged), brand name listing, Tofutti, Tofutti Cuties - Chocolate | Frozen treats | Soybean derived products (excluding milks) |
| ice cream and frozen desserts, treats (frozen prepackaged), brand name listing, Tofutti, Tofutti Cuties - Vanilla | Frozen treats | Soybean derived products (excluding milks) |
| ice cream and frozen desserts, treats (frozen prepackaged), brand name listing, Weight Watchers, Weight Watchers Chocolate Mousse Bar | Frozen treats | Milk desserts, frozen |
| ice cream and frozen desserts, treats (frozen prepackaged), brand name listing, Weight Watchers, Weight Watchers English Toffee Crunch Bar | Frozen treats | Milk desserts, frozen |
| ice cream and frozen desserts, treats (frozen prepackaged), brand name listing, Weight Watchers, Weight Watchers Orange Vanilla Treat | Frozen treats | Milk desserts, frozen |
| ice cream and frozen desserts, treats (frozen prepackaged), brand name listing, Welch's, Welch's Tropical Blend Fruit Juice Bar - all flavors | Frozen treats | Mixtures of fruits and nonfruit items |
| ice cream and frozen desserts, treats (frozen prepackaged), brand name listing, Wells' Blue Bunny, Blue Bunny Bomb Pop - all flavors | Frozen treats | Ices and popsicles |
| ice cream and frozen desserts, treats (frozen prepackaged), brand name listing, Wells' Blue Bunny, Blue Bunny Bozo Float Bar | Frozen treats | Milk desserts, frozen |
| ice cream and frozen desserts, treats (frozen prepackaged), brand name listing, Wells' Blue Bunny, Blue Bunny Cherry Screwball Cone | Frozen treats | Ices and popsicles |
| ice cream and frozen desserts, treats (frozen prepackaged), brand name listing, Wells' Blue Bunny, Blue Bunny Chocolate Creme Bar | Frozen treats | Milk desserts, frozen |
| ice cream and frozen desserts, treats (frozen prepackaged), brand name listing, Wells' Blue Bunny, Blue Bunny Citrus Snacks | Frozen treats | Mixtures of fruits and nonfruit items |
| ice cream and frozen desserts, treats (frozen prepackaged), brand name listing, Wells' Blue Bunny, Blue Bunny Cool Tubes Orange Sherbet Push-Up | Frozen treats | Milk desserts, frozen |
| ice cream and frozen desserts, treats (frozen prepackaged), brand name listing, Wells' Blue Bunny, Blue Bunny Chocolate Fudge Stick | Frozen treats | Milk desserts, frozen |
| ice cream and frozen desserts, treats (frozen prepackaged), brand name listing, Wells' Blue Bunny, Blue Bunny English Toffee Stick | Frozen treats | Milk desserts, frozen |
| ice cream and frozen desserts, treats (frozen prepackaged), brand name listing, Wells' Blue Bunny, Blue Bunny Banana Fudge Bomb | Frozen treats | Milk desserts, frozen |
| ice cream and frozen desserts, treats (frozen prepackaged), brand name listing, Wells' Blue Bunny, Blue Bunny Ice Cream Candy Bar - caramel nut nutt'n better | Frozen treats | Milk desserts, frozen |
| ice cream and frozen desserts, treats (frozen prepackaged), brand name listing, Wells' Blue Bunny, Blue Bunny Ice Cream Bar - Heath | Frozen treats | Milk desserts, frozen |
| ice cream and frozen desserts, treats (frozen prepackaged), brand name listing, Wells' Blue Bunny, Blue Bunny Ice Cream Cup - chocolate | Ice cream, ice milk, sherbet, nondairy frozen dessert, and milkshakes | Milk desserts, frozen |
| ice cream and frozen desserts, treats (frozen prepackaged), brand name listing, Wells' Blue Bunny, Blue Bunny Ice Cream Cup - chocolate marble | Ice cream, ice milk, sherbet, nondairy frozen dessert, and milkshakes | Milk desserts, frozen |
| ice cream and frozen desserts, treats (frozen prepackaged), brand name listing, Wells' Blue Bunny, Blue Bunny Ice Cream Cup - strawberry | Ice cream, ice milk, sherbet, nondairy frozen dessert, and milkshakes | Milk desserts, frozen |
| ice cream and frozen desserts, treats (frozen prepackaged), brand name listing, Wells' Blue Bunny, Blue Bunny Ice Cream Cup - vanilla | Ice cream, ice milk, sherbet, nondairy frozen dessert, and milkshakes | Milk desserts, frozen |
| ice cream and frozen desserts, treats (frozen prepackaged), brand name listing, Wells' Blue Bunny, Blue Bunny Ice Cream Sandwich - vanilla | Frozen treats | Milk desserts, frozen |
| ice cream and frozen desserts, treats (frozen prepackaged), brand name listing, Wells' Blue Bunny, Blue Bunny Ice Cream Sandwich - homemade vanilla | Frozen treats | Milk desserts, frozen |
| ice cream and frozen desserts, treats (frozen prepackaged), brand name listing, Wells' Blue Bunny, Blue Bunny Ice Cream Sandwich - mini | Frozen treats | Milk desserts, frozen |
| ice cream and frozen desserts, treats (frozen prepackaged), brand name listing, Wells' Blue Bunny, Blue Bunny Ice Cream Sandwich - Mississippi mud | Frozen treats | Milk desserts, frozen |
| ice cream and frozen desserts, treats (frozen prepackaged), brand name listing, Wells' Blue Bunny, Blue Bunny Ice Cream Sandwich - neapolitan | Frozen treats | Milk desserts, frozen |
| ice cream and frozen desserts, treats (frozen prepackaged), brand name listing, Wells' Blue Bunny, Blue Bunny Juice Bomb Pop Jr's - all flavors | Frozen treats | Mixtures of fruits and nonfruit items |
| ice cream and frozen desserts, treats (frozen prepackaged), brand name listing, Wells' Blue Bunny, Blue Bunny Krunch Stick | Frozen treats | Milk desserts, frozen |
| ice cream and frozen desserts, treats (frozen prepackaged), brand name listing, Wells' Blue Bunny, Blue Bunny Mini Pop Jr. | Frozen treats | Ices and popsicles |
| ice cream and frozen desserts, treats (frozen prepackaged), brand name listing, Wells' Blue Bunny, Blue Bunny Ninja Turtle Face Bar - all flavors | Frozen treats | Ices and popsicles |
| ice cream and frozen desserts, treats (frozen prepackaged), brand name listing, Wells' Blue Bunny, Blue Bunny Frozen Yogurt and Fruit Snacks - nonfat | Frozen treats | Milk desserts, frozen |
| ice cream and frozen desserts, treats (frozen prepackaged), brand name listing, Wells' Blue Bunny, Blue Bunny Vanilla Nutty Sundae Cone | Frozen treats | Milk desserts, frozen |
| ice cream and frozen desserts, treats (frozen prepackaged), brand name listing, Wells' Blue Bunny, Blue Bunny Peanut Butter Stick | Frozen treats | Milk desserts, frozen |
| ice cream and frozen desserts, treats (frozen prepackaged), brand name listing, Wells' Blue Bunny, Blue Bunny Pink Panther Face Bar | Frozen treats | Ices and popsicles |
| ice cream and frozen desserts, treats (frozen prepackaged), brand name listing, Wells' Blue Bunny, Blue Bunny Polar Pop - all flavors | Frozen treats | Ices and popsicles |
| ice cream and frozen desserts, treats (frozen prepackaged), brand name listing, Wells' Blue Bunny, Blue Bunny Premium Homemade Vanilla Ice Cream Bar | Frozen treats | Milk desserts, frozen |
| ice cream and frozen desserts, treats (frozen prepackaged), brand name listing, Wells' Blue Bunny, Blue Bunny Cherry Slush Bunny | Frozen treats | Ices and popsicles |
| ice cream and frozen desserts, treats (frozen prepackaged), brand name listing, Wells' Blue Bunny, Blue Bunny Slush Pop - all flavors | Frozen treats | Ices and popsicles |
| ice cream and frozen desserts, treats (frozen prepackaged), brand name listing, Wells' Blue Bunny, Blue Bunny Star Stick | Frozen treats | Milk desserts, frozen |
| ice cream and frozen desserts, treats (frozen prepackaged), brand name listing, Wells' Blue Bunny, Blue Bunny Sundae Crunch Bar - chocolate | Frozen treats | Milk desserts, frozen |
| ice cream and frozen desserts, treats (frozen prepackaged), brand name listing, Wells' Blue Bunny, Blue Bunny Sundae Crunch Bar - strawberry | Frozen treats | Milk desserts, frozen |
| ice cream and frozen desserts, treats (frozen prepackaged), brand name listing, Wells' Blue Bunny, Blue Bunny Sweet Freedom Bomb Pop Jr. | Frozen treats | Ices and popsicles |
| ice cream and frozen desserts, treats (frozen prepackaged), brand name listing, Wells' Blue Bunny, Blue Bunny Sweet Freedom Fudge Lite Bar | Frozen treats | Milk desserts, frozen |
| ice cream and frozen desserts, treats (frozen prepackaged), brand name listing, Wells' Blue Bunny, Blue Bunny Sweet Freedom Ice Cream Sandwich - reduced fat | Frozen treats | Milk desserts, frozen |
| ice cream and frozen desserts, treats (frozen prepackaged), brand name listing, Wells' Blue Bunny, Blue Bunny Sweet Freedom Krunch Lite | Frozen treats | Milk desserts, frozen |
| ice cream and frozen desserts, treats (frozen prepackaged), brand name listing, Wells' Blue Bunny, Blue Bunny Teenage Mutant Ninja Turtle Squeeze Up | Frozen treats | Milk desserts, frozen |
| ice cream and frozen desserts, treats (frozen prepackaged), brand name listing, Wells' Blue Bunny, Blue Bunny Twin Pop - all flavors | Frozen treats | Ices and popsicles |
| ice cream and frozen desserts, treats (frozen prepackaged), brand name listing, TCBY, TCBY Yogwich | Frozen treats | Milk desserts, frozen |
| ice cream and frozen desserts, treats (frozen prepackaged), drumstick (sundae cone), regular | Frozen treats | Milk desserts, frozen |
| ice cream and frozen desserts, treats (frozen prepackaged), fudge type bar (fudgesicle), no sugar added | Frozen treats | Milk desserts, frozen |
| desserts - miscellaneous, snacks - commercial packaged, cupcake (commercially packaged), low fat | Commercial snack cakes and muffins | Cakes |
| ice cream and frozen desserts, treats (frozen prepackaged), brand name listing, Wells' Blue Bunny, Blue Bunny Banana Pop | Frozen treats | Ices and popsicles |
| ice cream and frozen desserts, treats (frozen prepackaged), brand name listing, Wells' Blue Bunny, Blue Bunny Chocolate Malt Ice Cream Krunch Bomb | Frozen treats | Milk desserts, frozen |
| ice cream and frozen desserts, treats (frozen prepackaged), brand name listing, Wells' Blue Bunny, Blue Bunny Chocolate Vanilla Pudding Bar | Frozen treats | Milk desserts, frozen |
| ice cream and frozen desserts, treats (frozen prepackaged), brand name listing, Wells' Blue Bunny, Blue Bunny Crazy Peanut Stick | Frozen treats | Milk desserts, frozen |
| ice cream and frozen desserts, treats (frozen prepackaged), brand name listing, Wells' Blue Bunny, Blue Bunny Crunch Bar | Frozen treats | Milk desserts, frozen |
| ice cream and frozen desserts, treats (frozen prepackaged), brand name listing, Wells' Blue Bunny, Blue Bunny All American Super Chiller Pops | Frozen treats | Ices and popsicles |
| ice cream and frozen desserts, treats (frozen prepackaged), brand name listing, Wells' Blue Bunny, Blue Bunny Tazmanian Devil Face | Frozen treats | Ices and popsicles |
| desserts - miscellaneous, snacks - commercial packaged, brand name listing, Dolly Madison, Dolly Madison Banana Flip | Commercial snack cakes and muffins | Cakes |
| desserts - miscellaneous, snacks - commercial packaged, brand name listing, Dolly Madison, Dolly Madison Crumb Cake - low fat | Commercial snack cakes and muffins | Coffee cakes, not yeast |
| desserts - miscellaneous, snacks - commercial packaged, brand name listing, Dolly Madison, Dolly Madison Donut Gems - Crunch | Commercial snack doughnuts | Danish, breakfast pastries, doughnuts, granola bars |
| desserts - miscellaneous, snacks - commercial packaged, brand name listing, Dolly Madison, Dolly Madison Dunkin Stix | Commercial snack doughnuts | Danish, breakfast pastries, doughnuts, granola bars |
| desserts - miscellaneous, snacks - commercial packaged, brand name listing, Dolly Madison, Dolly Madison Muffins - Blueberry | Commercial snack cakes and muffins | Other muffins, popovers |
| desserts - miscellaneous, snacks - commercial packaged, brand name listing, Dolly Madison, Dolly Madison Pie - Cherry | Commercial snack pies | Pies |
| desserts - miscellaneous, snacks - commercial packaged, brand name listing, Dolly Madison, Dolly Madison Pie - Lemon | Commercial snack pies | Pies |
| desserts - miscellaneous, snacks - commercial packaged, brand name listing, Dolly Madison, Dolly Madison Pie - Peach | Commercial snack pies | Pies |
| desserts - miscellaneous, snacks - commercial packaged, brand name listing, Dolly Madison, Dolly Madison Snack Squares | Commercial snack cakes and muffins | Cakes |
| desserts - miscellaneous, snacks - commercial packaged, brand name listing, Dolly Madison, Dolly Madison Sweet Roll - Cherry | Commercial snack sweet rolls and coffee cakes | White breads, rolls |
| desserts - miscellaneous, snacks - commercial packaged, brand name listing, Dolly Madison, Dolly Madison Sweet Roll - Raspberry | Commercial snack sweet rolls and coffee cakes | White breads, rolls |
| desserts - miscellaneous, snacks - commercial packaged, brand name listing, Dolly Madison, Dolly Madison Zingers - Yellow | Commercial snack cakes and muffins | Cakes |
| desserts - miscellaneous, snacks - commercial packaged, brand name listing, Drake's, Drake's All Butter Pound Cake | Commercial snack cakes and muffins | Cakes |
| desserts - miscellaneous, snacks - commercial packaged, brand name listing, Drake's, Drake's Boston Creme | Commercial snack cakes and muffins | Cakes |
| desserts - miscellaneous, snacks - commercial packaged, brand name listing, Drake's, Drake's Coffee Cake | Commercial snack sweet rolls and coffee cakes | Coffee cakes, not yeast |
| desserts - miscellaneous, snacks - commercial packaged, brand name listing, Drake's, Drake's Coffee Cake Jr. | Commercial snack sweet rolls and coffee cakes | Coffee cakes, not yeast |
| desserts - miscellaneous, snacks - commercial packaged, brand name listing, Drake's, Drake's Funny Bones | Commercial snack cakes and muffins | Cakes |
| desserts - miscellaneous, snacks - commercial packaged, brand name listing, Drake's, Drake's Pies - Apple | Commercial snack pies | Pies |
| desserts - miscellaneous, snacks - commercial packaged, brand name listing, Drake's, Drake's Pies - Blueberry Fruit | Commercial snack pies | Pies |
| desserts - miscellaneous, snacks - commercial packaged, brand name listing, Drake's, Drake's Pies - Cherry | Commercial snack pies | Pies |
| desserts - miscellaneous, snacks - commercial packaged, brand name listing, Drake's, Drake's Pies - Lemon | Commercial snack pies | Pies |
| desserts - miscellaneous, snacks - commercial packaged, brand name listing, Drake's, Drake's Sunny Doodles | Commercial snack cakes and muffins | Cakes |
| desserts - miscellaneous, snacks - commercial packaged, brand name listing, Drake's, Drake's Yodels | Commercial snack cakes and muffins | Cakes |
| desserts - miscellaneous, snacks - commercial packaged, brand name listing, Hostess, Hostess Caramel Pecan Swirls | Commercial snack sweet rolls and coffee cakes | Danish, breakfast pastries, doughnuts, granola bars |
| desserts - miscellaneous, snacks - commercial packaged, brand name listing, Hostess, Hostess Chocolicious | Commercial snack cakes and muffins | Cakes |
| desserts - miscellaneous, snacks - commercial packaged, brand name listing, Hostess, Hostess Cinnaminis | Commercial snack sweet rolls and coffee cakes | White breads, rolls |
| desserts - miscellaneous, snacks - commercial packaged, brand name listing, Hostess, Hostess Cinnamon Roll | Commercial snack sweet rolls and coffee cakes | White breads, rolls |
| desserts - miscellaneous, snacks - commercial packaged, brand name listing, Hostess, Hostess Cinnamon Crumb Cake light | Commercial snack sweet rolls and coffee cakes | Coffee cakes, not yeast |
| desserts - miscellaneous, snacks - commercial packaged, brand name listing, Hostess, Hostess Donuts - Cinnamon | Commercial snack doughnuts | Danish, breakfast pastries, doughnuts, granola bars |
| desserts - miscellaneous, snacks - commercial packaged, brand name listing, Hostess, Hostess Donuts - Crumb | Commercial snack doughnuts | Danish, breakfast pastries, doughnuts, granola bars |
| desserts - miscellaneous, snacks - commercial packaged, brand name listing, Hostess, Hostess Donuts - Chocolate Frosted | Commercial snack doughnuts | Danish, breakfast pastries, doughnuts, granola bars |
| desserts - miscellaneous, snacks - commercial packaged, brand name listing, Hostess, Hostess Donuts - Glazed Party | Commercial snack doughnuts | Danish, breakfast pastries, doughnuts, granola bars |
| desserts - miscellaneous, snacks - commercial packaged, brand name listing, Hostess, Hostess Donuts - Glazed Whirl | Commercial snack doughnuts | Danish, breakfast pastries, doughnuts, granola bars |
| desserts - miscellaneous, snacks - commercial packaged, brand name listing, Hostess, Hostess Donuts - Jumbo, Frosted | Commercial snack doughnuts | Danish, breakfast pastries, doughnuts, granola bars |
| desserts - miscellaneous, snacks - commercial packaged, brand name listing, Hostess, Hostess Donuts - Jumbo, Plain | Commercial snack doughnuts | Danish, breakfast pastries, doughnuts, granola bars |
| desserts - miscellaneous, snacks - commercial packaged, brand name listing, Hostess, Hostess Donuts - Jumbo, Powdered | Commercial snack doughnuts | Danish, breakfast pastries, doughnuts, granola bars |
| desserts - miscellaneous, snacks - commercial packaged, brand name listing, Hostess, Hostess Donuts - Mini, Chocolate | Commercial snack doughnuts | Danish, breakfast pastries, doughnuts, granola bars |
| desserts - miscellaneous, snacks - commercial packaged, brand name listing, Hostess, Hostess Donettes - Cinnamon | Commercial snack doughnuts | Danish, breakfast pastries, doughnuts, granola bars |
| desserts - miscellaneous, snacks - commercial packaged, brand name listing, Hostess, Hostess Donettes - Crumb | Commercial snack doughnuts | Danish, breakfast pastries, doughnuts, granola bars |
| desserts - miscellaneous, snacks - commercial packaged, brand name listing, Hostess, Hostess Donettes - Frosted Strawberry Filled | Commercial snack doughnuts | Danish, breakfast pastries, doughnuts, granola bars |
| desserts - miscellaneous, snacks - commercial packaged, brand name listing, Hostess, Hostess Donettes - Powdered Strawberry Filled | Commercial snack doughnuts | Danish, breakfast pastries, doughnuts, granola bars |
| desserts - miscellaneous, snacks - commercial packaged, brand name listing, Hostess, Hostess Holiday Cakes | Commercial snack cakes and muffins | Cakes |
| desserts - miscellaneous, snacks - commercial packaged, brand name listing, Hostess, Hostess Honey Bun - Iced | Commercial snack sweet rolls and coffee cakes | White breads, rolls |
| desserts - miscellaneous, snacks - commercial packaged, brand name listing, Hostess, Hostess Hopper Cakes | Commercial snack cakes and muffins | Cakes |
| desserts - miscellaneous, snacks - commercial packaged, brand name listing, Hostess, Hostess Muffin - Oat Bran | Commercial snack cakes and muffins | Other muffins, popovers |
| desserts - miscellaneous, snacks - commercial packaged, brand name listing, Hostess, Hostess Muffin - Oat Bran Banana Nut | Commercial snack cakes and muffins | Other muffins, popovers |
| desserts - miscellaneous, snacks - commercial packaged, brand name listing, Hostess, Hostess Muffin - Mini, Chocolate Chip | Commercial snack cakes and muffins | Other muffins, popovers |
| desserts - miscellaneous, snacks - commercial packaged, brand name listing, Hostess, Hostess Muffin - Mini, Cinnamon Apple | Commercial snack cakes and muffins | Other muffins, popovers |
| desserts - miscellaneous, snacks - commercial packaged, brand name listing, Hostess, Hostess Muffin Loaf - Blueberry | Commercial snack cakes and muffins | Other quick breads |
| desserts - miscellaneous, snacks - commercial packaged, brand name listing, Hostess, Hostess Pecan Spinners | Commercial snack sweet rolls and coffee cakes | Danish, breakfast pastries, doughnuts, granola bars |
| desserts - miscellaneous, snacks - commercial packaged, brand name listing, Hostess, Hostess Twinkies - Strawberry Creme | Commercial snack cakes and muffins | Cakes |
| desserts - miscellaneous, snacks - commercial packaged, brand name listing, Hostess, Hostess Twinkies low fat | Commercial snack cakes and muffins | Cakes |
| desserts - miscellaneous, snacks - commercial packaged, brand name listing, Little Debbie, Little Debbie Muffin Loaf - Banana Nut | Commercial snack cakes and muffins | Other quick breads |
| desserts - miscellaneous, snacks - commercial packaged, brand name listing, Little Debbie, Little Debbie Christmas Tree Cakes - Chocolate Frosted | Commercial snack cakes and muffins | Cakes |
| desserts - miscellaneous, snacks - commercial packaged, brand name listing, Little Debbie, Little Debbie Coffee Cake - Apple Streusel | Commercial snack sweet rolls and coffee cakes | Coffee cakes, not yeast |
| desserts - miscellaneous, snacks - commercial packaged, brand name listing, Little Debbie, Little Debbie Honey Buns | Commercial snack sweet rolls and coffee cakes | White breads, rolls |
| desserts - miscellaneous, snacks - commercial packaged, brand name listing, Little Debbie, Little Debbie Oatmeal Lights - low fat | Commercial snack cakes and muffins | Cookies |
| desserts - miscellaneous, snacks - commercial packaged, brand name listing, Little Debbie, Little Debbie Pecan Spinwheels | Commercial snack sweet rolls and coffee cakes | Danish, breakfast pastries, doughnuts |
| desserts - miscellaneous, snacks - commercial packaged, brand name listing, Little Debbie, Little Debbie Swiss Roll | Commercial snack cakes and muffins | Cakes |
| desserts - miscellaneous, snacks - commercial packaged, brand name listing, Little Debbie, Little Debbie Zebra Cakes | Commercial snack cakes and muffins | Cakes |
| desserts - miscellaneous, snacks - commercial packaged, brand name listing, Moon Pie, Moon Pie - Chocolate | Commercial snack cakes and muffins | Cookies |
| desserts - miscellaneous, snacks - commercial packaged, brand name listing, Moon Pie, Moon Pie - Vanilla | Commercial snack cakes and muffins | Cookies |
| desserts - miscellaneous, snacks - commercial packaged, brand name listing, Pepperidge Farm, Pepperidge Farm Apple Fruit Square | Commercial snack pies | Cobblers, eclairs, turnovers, other pastries |
| desserts - miscellaneous, snacks - commercial packaged, brand name listing, Pepperidge Farm, Pepperidge Farm Cinnamon Roll | Commercial snack sweet rolls and coffee cakes | White breads, rolls |
| desserts - miscellaneous, snacks - commercial packaged, brand name listing, Pepperidge Farm, Pepperidge Farm Danish - Apple | Commercial snack sweet rolls and coffee cakes | Danish, breakfast pastries, doughnuts, granola bars |
| desserts - miscellaneous, snacks - commercial packaged, brand name listing, Pepperidge Farm, Pepperidge Farm Danish - Cheese | Commercial snack sweet rolls and coffee cakes | Danish, breakfast pastries, doughnuts, granola bars |
| desserts - miscellaneous, snacks - commercial packaged, brand name listing, Pepperidge Farm, Pepperidge Farm Danish - Raspberry | Commercial snack sweet rolls and coffee cakes | Danish, breakfast pastries, doughnuts, granola bars |
| desserts - miscellaneous, snacks - commercial packaged, brand name listing, Pepperidge Farm, Pepperidge Farm Wholesome Choice Muffin - Apple Oatmeal | Commercial snack cakes and muffins | Other muffins, popovers |
| desserts - miscellaneous, snacks - commercial packaged, brand name listing, Pepperidge Farm, Pepperidge Farm Wholesome Choice Muffin - Blueberry | Commercial snack cakes and muffins | Other muffins, popovers |
| desserts - miscellaneous, snacks - commercial packaged, brand name listing, Pepperidge Farm, Pepperidge Farm Wholesome Choice Muffin - Bran with Raisins | Commercial snack cakes and muffins | Other muffins, popovers |
| desserts - miscellaneous, snacks - commercial packaged, brand name listing, Pepperidge Farm, Pepperidge Farm Wholesome Choice Muffin - Corn | Commercial snack cakes and muffins | Cornbread, corn muffins, tortillas |
| desserts - miscellaneous, snacks - commercial packaged, brand name listing, Pillsbury, Pillsbury Turnover - Cherry | Commercial snack - miscellaneous desserts | Cobblers, eclairs, turnovers, other pastries |
| desserts - miscellaneous, snacks - commercial packaged, brand name listing, Tastykake, Tastykake Cupcake Mini - Cream Filled Butter Cream Iced | Commercial snack cakes and muffins | Cakes |
| desserts - miscellaneous, snacks - commercial packaged, brand name listing, Tastykake, Tastykake Cupcake Mini - Cream Filled Chocolate Iced | Commercial snack cakes and muffins | Cakes |
| desserts - miscellaneous, snacks - commercial packaged, brand name listing, Tastykake, Tastykake Cupcake Mini - Koffee Kake | Commercial snack cakes and muffins | Cakes |
| desserts - miscellaneous, snacks - commercial packaged, brand name listing, Tastykake, Tastykake Cupcake Mini - Vanilla Cream Filled Chocolate Iced | Commercial snack cakes and muffins | Cakes |
| desserts - miscellaneous, snacks - commercial packaged, brand name listing, Tastykake, Tastykake Donuts - Plain | Commercial snack doughnuts | Danish, breakfast pastries, doughnuts, granola bars |
| desserts - miscellaneous, snacks - commercial packaged, brand name listing, Tastykake, Tastykake Donuts Mini - Honey Wheat | Commercial snack doughnuts | Danish, breakfast pastries, doughnuts, granola bars |
| desserts - miscellaneous, snacks - commercial packaged, brand name listing, Tastykake, Tastykake Donuts Mini - Powdered Sugar | Commercial snack doughnuts | Danish, breakfast pastries, doughnuts, granola bars |
| desserts - miscellaneous, snacks - commercial packaged, brand name listing, Tastykake, Tastykake Pastry Pocket - Apple | Commercial snack pies | Cobblers, eclairs, turnovers, other pastries |
| desserts - miscellaneous, snacks - commercial packaged, brand name listing, Tastykake, Tastykake Pastry Pocket - Cheese | Commercial snack pies | Cobblers, eclairs, turnovers, other pastries |
| desserts - miscellaneous, snacks - commercial packaged, brand name listing, Tastykake, Tastykake Pastry Pocket - Cherry | Commercial snack pies | Cobblers, eclairs, turnovers, other pastries |
| desserts - miscellaneous, snacks - commercial packaged, brand name listing, Tastykake, Tastykake Cupcake - Cream Filled Chocolate lowfat | Commercial snack cakes and muffins | Cakes |
| desserts - miscellaneous, snacks - commercial packaged, brand name listing, Tastykake, Tastykake Cupcake - Vanilla low fat | Commercial snack cakes and muffins | Cakes |
| desserts - miscellaneous, snacks - commercial packaged, brand name listing, Weight Watchers, Weight Watchers Smart Ones Brownie A La Mode | Commercial snack - miscellaneous desserts | Cookies |
| desserts - miscellaneous, snacks - commercial packaged, brand name listing, Weight Watchers, Weight Watchers Smart Ones Chocolate Chip Cookie Dough Sundae | Commercial snack - miscellaneous desserts | Milk desserts, frozen |
| desserts - miscellaneous, snacks - commercial packaged, brand name listing, Weight Watchers, Weight Watchers Smart Ones Chocolate Eclair | Commercial snack - miscellaneous desserts | Cobblers, eclairs, turnovers, other pastries |
| desserts - miscellaneous, snacks - commercial packaged, brand name listing, Weight Watchers, Weight Watchers Double Fudge Brownie Parfait | Commercial snack - miscellaneous desserts | Milk desserts, frozen |
| desserts - miscellaneous, snacks - commercial packaged, brand name listing, Weight Watchers, Weight Watchers Smart Ones Double Fudge Cake | Commercial snack cakes and muffins | Cakes |
| desserts - miscellaneous, snacks - commercial packaged, brand name listing, Weight Watchers, Weight Watchers Mississippi Mud Pie | Commercial snack pies | Pies |
| desserts - miscellaneous, snacks - commercial packaged, brand name listing, Weight Watchers, Weight Watchers Peanut Butter Fudge Brownie | Cookies | Cookies |
| desserts - miscellaneous, snacks - commercial packaged, brand name listing, Weight Watchers, Weight Watchers Praline Toffee Crunch Parfait | Commercial snack - miscellaneous desserts | Milk desserts, frozen |
| desserts - miscellaneous, snacks - commercial packaged, brand name listing, Weight Watchers, Weight Watchers Triple Chocolate Caramel Mousse | Commercial snack - miscellaneous desserts | Puddings, custards, and other milk desserts |
| desserts - miscellaneous, snacks - commercial packaged, brand name listing, Weight Watchers, Weight Watchers Triple Chocolate Cheesecake | Commercial snack cakes and muffins | Cakes |
| desserts - miscellaneous, snacks - commercial packaged, brand name listing, Hostess, Hostess King Dons | Commercial snack cakes and muffins | Cakes |
| desserts - miscellaneous, snacks - commercial packaged, brand name listing, Tastykake, Tastykake Pie - Blueberry | Commercial snack pies | Pies |
| desserts - miscellaneous, snacks - commercial packaged, brand name listing, Little Debbie, Little Debbie Banana Twins | Commercial snack cakes and muffins | Cakes |
| desserts - miscellaneous, snacks - commercial packaged, brand name listing, Tastykake, Tastykake Donuts Mini - Rich Frosted | Commercial snack doughnuts | Danish, breakfast pastries, doughnuts, granola bars |
| desserts - miscellaneous, snacks - commercial packaged, brand name listing, Hostess, Hostess Donuts - Old Fashioned, Glazed, Honey Wheat | Commercial snack doughnuts | Danish, breakfast pastries, doughnuts, granola bars |
| desserts - miscellaneous, snacks - commercial packaged, brand name listing, Hostess, Hostess Muffin - Mini, Banana Nut | Commercial snack cakes and muffins | Other muffins, popovers |
| desserts - miscellaneous, snacks - commercial packaged, brand name listing, Little Debbie, Little Debbie Angel Cakes - lowfat Raspberry | Commercial snack cakes and muffins | Cakes |
| desserts - miscellaneous, snacks - commercial packaged, brand name listing, Entenmann's, Entenmann's All Butter French Crumb Cake - 50% less fat | Commercial snack cakes and muffins | Coffee cakes, not yeast |
| desserts - miscellaneous, snacks - commercial packaged, brand name listing, Entenmann's, Entenmann's All Butter Loaf - 50% less fat | Commercial snack cakes and muffins | Cakes |
| desserts - miscellaneous, snacks - commercial packaged, brand name listing, Entenmann's, Entenmann's All Butter Marble Loaf - 50% less fat | Commercial snack cakes and muffins | Cakes |
| desserts - miscellaneous, snacks - commercial packaged, brand name listing, Entenmann's, Entenmann's Apple Beehive Pie - fat free | Commercial snack pies | Pies |
| desserts - miscellaneous, snacks - commercial packaged, brand name listing, Entenmann's, Entenmann's Apple Buns - fat free | Commercial snack sweet rolls and coffee cakes | Danish, breakfast pastries, doughnuts, granola bars |
| desserts - miscellaneous, snacks - commercial packaged, brand name listing, Entenmann's, Entenmann's Apple Pie - 50% less fat | Commercial snack pies | Pies |
| desserts - miscellaneous, snacks - commercial packaged, brand name listing, Entenmann's, Entenmann's Apple Spice Crumb Cake - fat free | Commercial snack sweet rolls and coffee cakes | Coffee cakes, not yeast |
| desserts - miscellaneous, snacks - commercial packaged, brand name listing, Entenmann's, Entenmann's Apple Topped Cake - fat free | Commercial snack cakes and muffins | Cakes |
| desserts - miscellaneous, snacks - commercial packaged, brand name listing, Entenmann's, Entenmann's Apricot Twist - fat free | Commercial snack sweet rolls and coffee cakes | Danish, breakfast pastries, doughnuts, granola bars |
| desserts - miscellaneous, snacks - commercial packaged, brand name listing, Entenmann's, Entenmann's Banana Crunch Cake - 50% less fat | Commercial snack cakes and muffins | Cakes |
| desserts - miscellaneous, snacks - commercial packaged, brand name listing, Entenmann's, Entenmann's Banana Loaf - fat free | Commercial snack cakes and muffins | Other quick breads |
| desserts - miscellaneous, snacks - commercial packaged, brand name listing, Entenmann's, Entenmann's Black Forest Pastry - fat free | Commercial snack sweet rolls and coffee cakes | Danish, breakfast pastries, doughnuts, granola bars |
| desserts - miscellaneous, snacks - commercial packaged, brand name listing, Entenmann's, Entenmann's Blackberry Topped Cake - fat free | Commercial snack cakes and muffins | Cakes |
| desserts - miscellaneous, snacks - commercial packaged, brand name listing, Entenmann's, Entenmann's Blueberry Cheese Buns - fat free | Commercial snack sweet rolls and coffee cakes | Danish, breakfast pastries, doughnuts, granola bars |
| desserts - miscellaneous, snacks - commercial packaged, brand name listing, Entenmann's, Entenmann's Blueberry Filled Cheesecake - fat free | Commercial snack cakes and muffins | Cakes |
| desserts - miscellaneous, snacks - commercial packaged, brand name listing, Entenmann's, Entenmann's Blueberry Muffins - fat free | Commercial snack cakes and muffins | Other muffins, popovers |
| desserts - miscellaneous, snacks - commercial packaged, brand name listing, Entenmann's, Entenmann's Caramel Sweet Rolls - fat free | Commercial snack sweet rolls and coffee cakes | White breads, rolls |
| desserts - miscellaneous, snacks - commercial packaged, brand name listing, Entenmann's, Entenmann's Carrot Cake - fat free | Commercial snack cakes and muffins | Cakes |
| desserts - miscellaneous, snacks - commercial packaged, brand name listing, Entenmann's, Entenmann's Cheese Coffee Cake - 50% less fat | Commercial snack sweet rolls and coffee cakes | Coffee cakes, not yeast |
| desserts - miscellaneous, snacks - commercial packaged, brand name listing, Entenmann's, Entenmann's Cheese Filled Crumb Coffee Cake - 40% less fat | Commercial snack sweet rolls and coffee cakes | Coffee cakes, not yeast |
| desserts - miscellaneous, snacks - commercial packaged, brand name listing, Entenmann's, Entenmann's Cheese Filled Crumb Pastry - fat free | Commercial snack sweet rolls and coffee cakes | Danish, breakfast pastries, doughnuts, granola bars |
| desserts - miscellaneous, snacks - commercial packaged, brand name listing, Entenmann's, Entenmann's Cheese Filled Ring - fat free | Commercial snack sweet rolls and coffee cakes | Danish, breakfast pastries, doughnuts, granola bars |
| desserts - miscellaneous, snacks - commercial packaged, brand name listing, Entenmann's, Entenmann's Cherry Beehive Pie - fat free | Commercial snack pies | Pies |
| desserts - miscellaneous, snacks - commercial packaged, brand name listing, Entenmann's, Entenmann's Chocolate Crumb Delight Cake - 50% less fat | Commercial snack cakes and muffins | Cakes |
| desserts - miscellaneous, snacks - commercial packaged, brand name listing, Entenmann's, Entenmann's Chocolate Crunch Cake - fat free | Commercial snack cakes and muffins | Cakes |
| desserts - miscellaneous, snacks - commercial packaged, brand name listing, Entenmann's, Entenmann's Chocolate Fudge Cake - 50% less fat | Commercial snack cakes and muffins | Cakes |
| desserts - miscellaneous, snacks - commercial packaged, brand name listing, Entenmann's, Entenmann's Golden Chocolatey Chip Loaf - fat free | Commercial snack cakes and muffins | Other quick breads |
| desserts - miscellaneous, snacks - commercial packaged, brand name listing, Entenmann's, Entenmann's Cinnamon Apple Twist - fat free | Commercial snack sweet rolls and coffee cakes | White breads, rolls |
| desserts - miscellaneous, snacks - commercial packaged, brand name listing, Entenmann's, Entenmann's Cinnamon Bun - 50% less fat | Commercial snack sweet rolls and coffee cakes | White breads, rolls |
| desserts - miscellaneous, snacks - commercial packaged, brand name listing, Entenmann's, Entenmann's Cinnamon Donut - 50% less fat | Commercial snack doughnuts | Danish, breakfast pastries, doughnuts, granola bars |
| desserts - miscellaneous, snacks - commercial packaged, brand name listing, Entenmann's, Entenmann's Cinnamon Raisin Bun - fat free | Commercial snack sweet rolls and coffee cakes | White breads, rolls |
| desserts - miscellaneous, snacks - commercial packaged, brand name listing, Entenmann's, Entenmann's Cinnamon Twist - fat free | Commercial snack sweet rolls and coffee cakes | White breads, rolls |
| desserts - miscellaneous, snacks - commercial packaged, brand name listing, Entenmann's, Entenmann's Cranberry Orange Oval - fat free | Commercial snack sweet rolls and coffee cakes | Danish, breakfast pastries, doughnuts, granola bars |
| desserts - miscellaneous, snacks - commercial packaged, brand name listing, Entenmann's, Entenmann's Crumb Delight - 50% less fat | Commercial snack cakes and muffins | Cakes |
| desserts - miscellaneous, snacks - commercial packaged, brand name listing, Entenmann's, Entenmann's Cupcakes - Chocolate Creme Filled fat free | Commercial snack cakes and muffins | Cakes |
| desserts - miscellaneous, snacks - commercial packaged, brand name listing, Entenmann's, Entenmann's Devil's Food Crumb Donut - 50% less fat | Commercial snack doughnuts | Danish, breakfast pastries, doughnuts, granola bars |
| desserts - miscellaneous, snacks - commercial packaged, brand name listing, Entenmann's, Entenmann's Dutch Apple Crumb Pie - fat free | Commercial snack pies | Pies |
| desserts - miscellaneous, snacks - commercial packaged, brand name listing, Entenmann's, Entenmann's Fantastic Fudge Donut - 50% less fat | Commercial snack doughnuts | Danish, breakfast pastries, doughnuts, granola bars |
| desserts - miscellaneous, snacks - commercial packaged, brand name listing, Entenmann's, Entenmann's Fudge Iced Chocolate Cake - fat free | Commercial snack cakes and muffins | Cakes |
| desserts - miscellaneous, snacks - commercial packaged, brand name listing, Entenmann's, Entenmann's Fudge Iced Golden Cake - 50% less fat | Commercial snack cakes and muffins | Cakes |
| desserts - miscellaneous, snacks - commercial packaged, brand name listing, Entenmann's, Entenmann's Fudge Iced Golden Cake - fat free | Commercial snack cakes and muffins | Cakes |
| desserts - miscellaneous, snacks - commercial packaged, brand name listing, Entenmann's, Entenmann's Glazed Devil's Food Donuts - 50% less fat | Commercial snack doughnuts | Danish, breakfast pastries, doughnuts, granola bars |
| desserts - miscellaneous, snacks - commercial packaged, brand name listing, Entenmann's, Entenmann's Glazed Donuts - 50% less fat | Commercial snack doughnuts | Danish, breakfast pastries, doughnuts, granola bars |
| desserts - miscellaneous, snacks - commercial packaged, brand name listing, Entenmann's, Entenmann's Golden Cake - fat free | Commercial snack cakes and muffins | Cakes |
| desserts - miscellaneous, snacks - commercial packaged, brand name listing, Entenmann's, Entenmann's Golden French Crumb Cake - fat free | Commercial snack cakes and muffins | Cakes |
| desserts - miscellaneous, snacks - commercial packaged, brand name listing, Entenmann's, Entenmann's Golden Lemon Delight - 50% less fat | Commercial snack cakes and muffins | Cakes |
| desserts - miscellaneous, snacks - commercial packaged, brand name listing, Entenmann's, Entenmann's Lemon Twist - fat free | Commercial snack sweet rolls and coffee cakes | Danish, breakfast pastries, doughnuts, granola bars |
| desserts - miscellaneous, snacks - commercial packaged, brand name listing, Entenmann's, Entenmann's Louisiana Crunch Cake - fat free | Commercial snack cakes and muffins | Cakes |
| desserts - miscellaneous, snacks - commercial packaged, brand name listing, Entenmann's, Entenmann's Marble Loaf - fat free | Commercial snack cakes and muffins | Cakes |
| desserts - miscellaneous, snacks - commercial packaged, brand name listing, Entenmann's, Entenmann's Marshmallow Iced Devil's Food Cake - fat free | Commercial snack cakes and muffins | Cakes |
| desserts - miscellaneous, snacks - commercial packaged, brand name listing, Entenmann's, Entenmann's Metropolitan Cake - 50% less fat | Commercial snack cakes and muffins | Cakes |
| desserts - miscellaneous, snacks - commercial packaged, brand name listing, Entenmann's, Entenmann's Mocha Iced Chocolate Cake - fat free | Commercial snack cakes and muffins | Cakes |
| desserts - miscellaneous, snacks - commercial packaged, brand name listing, Entenmann's, Entenmann's Old Fashioned Donuts - 50% less fat | Commercial snack doughnuts | Danish, breakfast pastries, doughnuts, granola bars |
| desserts - miscellaneous, snacks - commercial packaged, brand name listing, Entenmann's, Entenmann's Orange Crunch Loaf - 50% less fat | Commercial snack cakes and muffins | Other quick breads |
| desserts - miscellaneous, snacks - commercial packaged, brand name listing, Entenmann's, Entenmann's Orange Oval - fat free | Commercial snack sweet rolls and coffee cakes | Danish, breakfast pastries, doughnuts, granola bars |
| desserts - miscellaneous, snacks - commercial packaged, brand name listing, Entenmann's, Entenmann's Party Cupcakes - Eclair Creme Filled fat free | Commercial snack cakes and muffins | Cakes |
| desserts - miscellaneous, snacks - commercial packaged, brand name listing, Entenmann's, Entenmann's Peach Topped Cake - fat free | Commercial snack cakes and muffins | Cakes |
| desserts - miscellaneous, snacks - commercial packaged, brand name listing, Entenmann's, Entenmann's Pecan Danish Ring - 33% less fat | Commercial snack sweet rolls and coffee cakes | Danish, breakfast pastries, doughnuts, granola bars |
| desserts - miscellaneous, snacks - commercial packaged, brand name listing, Entenmann's, Entenmann's Pineapple Cheese Buns - fat free | Commercial snack sweet rolls and coffee cakes | Danish, breakfast pastries, doughnuts, granola bars |
| desserts - miscellaneous, snacks - commercial packaged, brand name listing, Entenmann's, Entenmann's Pineapple Filled Cheesecake - fat free | Commercial snack cakes and muffins | Cakes |
| desserts - miscellaneous, snacks - commercial packaged, brand name listing, Entenmann's, Entenmann's Pineapple Topped Cake - fat free | Commercial snack cakes and muffins | Cakes |
| desserts - miscellaneous, snacks - commercial packaged, brand name listing, Entenmann's, Entenmann's Powdered Sugar Donut - 50% less fat | Commercial snack doughnuts | Danish, breakfast pastries, doughnuts, granola bars |
| desserts - miscellaneous, snacks - commercial packaged, brand name listing, Entenmann's, Entenmann's Raisin Loaf - fat free | Commercial snack cakes and muffins | Other quick breads |
| desserts - miscellaneous, snacks - commercial packaged, brand name listing, Entenmann's, Entenmann's Raspberry Cheese Buns - fat free | Commercial snack sweet rolls and coffee cakes | Danish, breakfast pastries, doughnuts, granola bars |
| desserts - miscellaneous, snacks - commercial packaged, brand name listing, Entenmann's, Entenmann's Raspberry Cheese Pastry - fat free | Commercial snack sweet rolls and coffee cakes | Danish, breakfast pastries, doughnuts, granola bars |
| desserts - miscellaneous, snacks - commercial packaged, brand name listing, Entenmann's, Entenmann's Raspberry Cheese Ring - fat free | Commercial snack sweet rolls and coffee cakes | Danish, breakfast pastries, doughnuts, granola bars |
| desserts - miscellaneous, snacks - commercial packaged, brand name listing, Entenmann's, Entenmann's Raspberry Danish Twist - 50% less fat | Commercial snack sweet rolls and coffee cakes | Danish, breakfast pastries, doughnuts, granola bars |
| desserts - miscellaneous, snacks - commercial packaged, brand name listing, Entenmann's, Entenmann's Raspberry Filled Cheesecake - fat free | Commercial snack cakes and muffins | Cakes |
| desserts - miscellaneous, snacks - commercial packaged, brand name listing, Entenmann's, Entenmann's Raspberry Glazed Fudge Donut - 50% less fat | Commercial snack doughnuts | Danish, breakfast pastries, doughnuts, granola bars |
| desserts - miscellaneous, snacks - commercial packaged, brand name listing, Entenmann's, Entenmann's Sour Cream Chip & Nut Loaf - 50% less fat | Commercial snack cakes and muffins | Other quick breads |
| desserts - miscellaneous, snacks - commercial packaged, brand name listing, Entenmann's, Entenmann's Strawberry Loaf - 50% less fat | Commercial snack cakes and muffins | Other quick breads |
| desserts - miscellaneous, snacks - commercial packaged, brand name listing, Entenmann's, Entenmann's Strawberry Twist - fat free | Commercial snack sweet rolls and coffee cakes | Danish, breakfast pastries, doughnuts, granola bars |
| desserts - miscellaneous, snacks - commercial packaged, brand name listing, Entenmann's, Entenmann's Twist O' Lemon Loaf - 50% less fat | Commercial snack cakes and muffins | Other quick breads |
| desserts - miscellaneous, snacks - commercial packaged, brand name listing, Entenmann's, Entenmann's Walnut Danish Ring - 33% less fat | Commercial snack sweet rolls and coffee cakes | Danish, breakfast pastries, doughnuts, granola bars |
| desserts - miscellaneous, snacks - commercial packaged, brand name listing, Entenmann's, Entenmann's Wild Blueberry Crunch Loaf - 50% less fat | Commercial snack cakes and muffins | Other quick breads |
| desserts - miscellaneous, snacks - commercial packaged, brand name listing, Tastykake, Tastykake Creamies - Sprinkled | Commercial snack cakes and muffins | Cakes |
| desserts - miscellaneous, snacks - commercial packaged, brand name listing, Tastykake, Tastykake Witchy Treats | Commercial snack cakes and muffins | Cakes |
| desserts - miscellaneous, snacks - commercial packaged, brand name listing, Tastykake, Tastykake Dunkin Stix | Commercial snack doughnuts | Danish, breakfast pastries, doughnuts, granola bars |
| desserts - miscellaneous, snacks - commercial packaged, brand name listing, Tastykake, Tastykake Junior - Pound Kake | Commercial snack cakes and muffins | Cakes |
| desserts - miscellaneous, snacks - commercial packaged, brand name listing, Tastykake, Tastykake Kandy Kake - Frosty | Commercial snack cakes and muffins | Cakes |
| desserts - miscellaneous, snacks - commercial packaged, brand name listing, Tastykake, Tastykake Koffee Kake - low fat Apple Filled | Commercial snack sweet rolls and coffee cakes | Coffee cakes, not yeast |
| desserts - miscellaneous, snacks - commercial packaged, brand name listing, Tastykake, Tastykake Koffee Kake - low fat Lemon Filled | Commercial snack sweet rolls and coffee cakes | Coffee cakes, not yeast |
| desserts - miscellaneous, snacks - commercial packaged, brand name listing, Tastykake, Tastykake Cupcake - Raspberry Koffee Kake low fat | Commercial snack cakes and muffins | Cakes |
| desserts - miscellaneous, snacks - commercial packaged, brand name listing, Tastykake, Tastykake Kreme Krimpies | Commercial snack cakes and muffins | Cakes |
| desserts - miscellaneous, snacks - commercial packaged, brand name listing, Tastykake, Tastykake Krimpet - low fat Apple Filled | Commercial snack cakes and muffins | Cakes |
| desserts - miscellaneous, snacks - commercial packaged, brand name listing, Tastykake, Tastykake Krimpet - low fat Jelly Filled | Commercial snack cakes and muffins | Cakes |
| desserts - miscellaneous, snacks - commercial packaged, brand name listing, Tastykake, Tastykake Krimpet - low fat Lemon Filled | Commercial snack cakes and muffins | Cakes |
| desserts - miscellaneous, snacks - commercial packaged, brand name listing, Tastykake, Tastykake Muffin - Banana Nut | Commercial snack cakes and muffins | Other muffins, popovers |
| desserts - miscellaneous, snacks - commercial packaged, brand name listing, Tastykake, Tastykake Muffin - Blueberry | Commercial snack cakes and muffins | Other muffins, popovers |
| desserts - miscellaneous, snacks - commercial packaged, brand name listing, Tastykake, Tastykake Muffin - Blueberry low fat | Commercial snack cakes and muffins | Other muffins, popovers |
| desserts - miscellaneous, snacks - commercial packaged, brand name listing, Tastykake, Tastykake Muffin - Corn | Commercial snack cakes and muffins | Cornbread, corn muffins, tortillas |
| desserts - miscellaneous, snacks - commercial packaged, brand name listing, Tastykake, Tastykake Muffin - Cranberry Orange low fat | Commercial snack cakes and muffins | Other muffins, popovers |
| desserts - miscellaneous, snacks - commercial packaged, brand name listing, Tastykake, Tastykake Muffin - Raisin Bran low fat | Commercial snack cakes and muffins | Other muffins, popovers |
| desserts - miscellaneous, snacks - commercial packaged, brand name listing, Tastykake, Tastykake Pie - Apple | Commercial snack pies | Pies |
| desserts - miscellaneous, snacks - commercial packaged, brand name listing, Tastykake, Tastykake Pie - Cherry | Commercial snack pies | Pies |
| desserts - miscellaneous, snacks - commercial packaged, brand name listing, Tastykake, Tastykake Pie - Lemon | Commercial snack pies | Pies |
| desserts - miscellaneous, snacks - commercial packaged, brand name listing, Tastykake, Tastykake Pie - Peach | Commercial snack pies | Pies |
| desserts - miscellaneous, snacks - commercial packaged, brand name listing, Tastykake, Tastykake Pie - Pineapple | Commercial snack pies | Pies |
| desserts - miscellaneous, snacks - commercial packaged, brand name listing, Tastykake, Tastykake Pie - Strawberry | Commercial snack pies | Pies |
| desserts - miscellaneous, snacks - commercial packaged, brand name listing, Tastykake, Tastykake Whirly Twirls | Commercial snack sweet rolls and coffee cakes | Danish, breakfast pastries, doughnuts, granola bars |
| desserts - miscellaneous, snacks - commercial packaged, brand name listing, Little Debbie, Little Debbie Be My Valentine Cakes - Pink | Commercial snack cakes and muffins | Cakes |
| desserts - miscellaneous, snacks - commercial packaged, brand name listing, Little Debbie, Little Debbie Coffee Cake | Commercial snack sweet rolls and coffee cakes | Coffee cakes, not yeast |
| desserts - miscellaneous, snacks - commercial packaged, brand name listing, Little Debbie, Little Debbie Cupcakes - Chocolate | Commercial snack cakes and muffins | Cakes |
| desserts - miscellaneous, snacks - commercial packaged, brand name listing, Little Debbie, Little Debbie Easter Basket Cakes - Yellow | Commercial snack cakes and muffins | Cakes |
| desserts - miscellaneous, snacks - commercial packaged, brand name listing, Little Debbie, Little Debbie Fall Party Cakes - Chocolate | Commercial snack cakes and muffins | Cakes |
| desserts - miscellaneous, snacks - commercial packaged, brand name listing, Little Debbie, Little Debbie Fall Party Cakes - Vanilla | Commercial snack cakes and muffins | Cakes |
| desserts - miscellaneous, snacks - commercial packaged, brand name listing, Little Debbie, Little Debbie Frosted Fudge Cakes | Commercial snack cakes and muffins | Cakes |
| desserts - miscellaneous, snacks - commercial packaged, brand name listing, Little Debbie, Little Debbie Holiday Cake Rolls - Cherry Creme | Commercial snack cakes and muffins | Cakes |
| desserts - miscellaneous, snacks - commercial packaged, brand name listing, Little Debbie, Little Debbie Muffin Loaf - Blueberry | Commercial snack cakes and muffins | Other muffins, popovers |
| desserts - miscellaneous, snacks - commercial packaged, brand name listing, Little Debbie, Little Debbie Tiger Cakes | Commercial snack cakes and muffins | Cakes |
| desserts - miscellaneous, snacks - commercial packaged, brand name listing, Sara Lee, Sara Lee Banana Cake | Commercial snack cakes and muffins | Cakes |
| desserts - miscellaneous, snacks - commercial packaged, brand name listing, Sara Lee, Sara Lee Carrot Cake | Commercial snack cakes and muffins | Cakes |
| desserts - miscellaneous, snacks - commercial packaged, brand name listing, Sara Lee, Sara Lee Chocolate Cake | Commercial snack cakes and muffins | Cakes |
| desserts - miscellaneous, snacks - commercial packaged, brand name listing, Sara Lee, Sara Lee Cinnamon Roll | Commercial snack sweet rolls and coffee cakes | White breads, rolls |
| desserts - miscellaneous, snacks - commercial packaged, brand name listing, Sara Lee, Sara Lee Danish - Apple | Commercial snack sweet rolls and coffee cakes | Danish, breakfast pastries, doughnuts, granola bars |
| desserts - miscellaneous, snacks - commercial packaged, brand name listing, Sara Lee, Sara Lee Danish - Cheese | Commercial snack sweet rolls and coffee cakes | Danish, breakfast pastries, doughnuts, granola bars |
| desserts - miscellaneous, snacks - commercial packaged, brand name listing, Sara Lee, Sara Lee Danish - Cinnamon Raisin | Commercial snack sweet rolls and coffee cakes | Danish, breakfast pastries, doughnuts, granola bars |
| desserts - miscellaneous, snacks - commercial packaged, brand name listing, Sara Lee, Sara Lee Danish - Iced Cheese | Commercial snack sweet rolls and coffee cakes | Danish, breakfast pastries, doughnuts, granola bars |
| desserts - miscellaneous, snacks - commercial packaged, brand name listing, Sara Lee, Sara Lee Danish - Nut Caramel | Commercial snack sweet rolls and coffee cakes | Danish, breakfast pastries, doughnuts, granola bars |
| desserts - miscellaneous, snacks - commercial packaged, brand name listing, Sara Lee, Sara Lee Danish - Raspberry | Commercial snack sweet rolls and coffee cakes | Danish, breakfast pastries, doughnuts, granola bars |
| desserts - miscellaneous, snacks - commercial packaged, brand name listing, Sara Lee, Sara Lee Muffin - Banana Nut | Commercial snack cakes and muffins | Other muffins, popovers |
| desserts - miscellaneous, snacks - commercial packaged, brand name listing, Sara Lee, Sara Lee Muffin - Blueberry | Commercial snack cakes and muffins | Other muffins, popovers |
| desserts - miscellaneous, snacks - commercial packaged, brand name listing, Sara Lee, Sara Lee Muffin - Bran | Commercial snack cakes and muffins | Other muffins, popovers |
| desserts - miscellaneous, snacks - commercial packaged, brand name listing, Sara Lee, Sara Lee Muffin - Carrot Nut | Commercial snack cakes and muffins | Other muffins, popovers |
| desserts - miscellaneous, snacks - commercial packaged, brand name listing, Sara Lee, Sara Lee Muffin - Cheese Streusel | Commercial snack cakes and muffins | Other muffins, popovers |
| desserts - miscellaneous, snacks - commercial packaged, brand name listing, Sara Lee, Sara Lee Muffin - Chocolate Chunk | Commercial snack cakes and muffins | Other muffins, popovers |
| desserts - miscellaneous, snacks - commercial packaged, brand name listing, Sara Lee, Sara Lee Muffin - Corn | Commercial snack cakes and muffins | Cornbread, corn muffins, tortillas |
| desserts - miscellaneous, snacks - commercial packaged, brand name listing, Pillsbury, Pillsbury Apple Cinnamon Roll with Icing | Commercial snack sweet rolls and coffee cakes | White breads, rolls |
| desserts - miscellaneous, snacks - commercial packaged, brand name listing, Pillsbury, Pillsbury Caramel Roll | Commercial snack sweet rolls and coffee cakes | White breads, rolls |
| desserts - miscellaneous, snacks - commercial packaged, brand name listing, Pillsbury, Pillsbury Cinnamon Raisin Roll with Icing | Commercial snack sweet rolls and coffee cakes | White breads, rolls |
| desserts - miscellaneous, snacks - commercial packaged, brand name listing, Pillsbury, Pillsbury Cinnamon Roll with Icing - all flavors | Commercial snack sweet rolls and coffee cakes | White breads, rolls |
| desserts - miscellaneous, snacks - commercial packaged, brand name listing, Pillsbury, Pillsbury Orange Sweet Roll with Icing | Commercial snack sweet rolls and coffee cakes | White breads, rolls |
| desserts - miscellaneous, snacks - commercial packaged, brand name listing, Pepperidge Farm, Pepperidge Farm Dumpling - Peach | Commercial snack - miscellaneous desserts | Cobblers, eclairs, turnovers, other pastries |
| desserts - miscellaneous, snacks - commercial packaged, brand name listing, Pepperidge Farm, Pepperidge Farm Turnover - Apple Mini | Commercial snack - miscellaneous desserts | Cobblers, eclairs, turnovers, other pastries |
| desserts - miscellaneous, snacks - commercial packaged, brand name listing, Pepperidge Farm, Pepperidge Farm Turnover - Apple with Vanilla Icing | Commercial snack - miscellaneous desserts | Cobblers, eclairs, turnovers, other pastries |
| desserts - miscellaneous, snacks - commercial packaged, brand name listing, Pepperidge Farm, Pepperidge Farm Turnover - Cherry Mini | Commercial snack - miscellaneous desserts | Cobblers, eclairs, turnovers, other pastries |
| desserts - miscellaneous, snacks - commercial packaged, brand name listing, Pepperidge Farm, Pepperidge Farm Turnover - Cherry with Vanilla Icing | Commercial snack - miscellaneous desserts | Cobblers, eclairs, turnovers, other pastries |
| desserts - miscellaneous, snacks - commercial packaged, brand name listing, Pepperidge Farm, Pepperidge Farm Turnover - Peach Cobbler Mini | Commercial snack - miscellaneous desserts | Cobblers, eclairs, turnovers, other pastries |
| desserts - miscellaneous, snacks - commercial packaged, brand name listing, Pepperidge Farm, Pepperidge Farm Turnover - Raspberry with Vanilla Icing | Commercial snack - miscellaneous desserts | Cobblers, eclairs, turnovers, other pastries |
| desserts - miscellaneous, snacks - commercial packaged, brand name listing, Pepperidge Farm, Pepperidge Farm Turnover - Strawberry Mini | Commercial snack - miscellaneous desserts | Cobblers, eclairs, turnovers, other pastries |
| desserts - miscellaneous, snacks - commercial packaged, brand name listing, Hostess, Hostess Fruit Pie - Apple | Commercial snack pies | Pies |
| desserts - miscellaneous, snacks - commercial packaged, brand name listing, Hostess, Hostess Fruit Pie - Blueberry | Commercial snack pies | Pies |
| desserts - miscellaneous, snacks - commercial packaged, brand name listing, Hostess, Hostess Fruit Pie - Cherry | Commercial snack pies | Pies |
| desserts - miscellaneous, snacks - commercial packaged, brand name listing, Hostess, Hostess Fruit Pie - Lemon | Commercial snack pies | Pies |
| desserts - miscellaneous, snacks - commercial packaged, brand name listing, Hostess, Hostess Fruit Pie - Peach | Commercial snack pies | Pies |
| desserts - miscellaneous, snacks - commercial packaged, brand name listing, Hostess, Hostess Honey Bun - Frosted | Commercial snack sweet rolls and coffee cakes | White breads, rolls |
| desserts - miscellaneous, snacks - commercial packaged, brand name listing, Hostess, Hostess Muffin Loaf - Raspberry | Commercial snack cakes and muffins | Other muffins, popovers |
| desserts - miscellaneous, snacks - commercial packaged, brand name listing, Dolly Madison, Dolly Madison Cake Delights | Commercial snack cakes and muffins | Cakes |
| desserts - miscellaneous, snacks - commercial packaged, brand name listing, Dolly Madison, Dolly Madison Cherry Bun | Commercial snack sweet rolls and coffee cakes | Danish, breakfast pastries, doughnuts, granola bars |
| desserts - miscellaneous, snacks - commercial packaged, brand name listing, Dolly Madison, Dolly Madison Cinnamon Bun | Commercial snack sweet rolls and coffee cakes | White breads, rolls |
| desserts - miscellaneous, snacks - commercial packaged, brand name listing, Dolly Madison, Dolly Madison Coffee Cake | Commercial snack sweet rolls and coffee cakes | Coffee cakes, not yeast |
| desserts - miscellaneous, snacks - commercial packaged, brand name listing, Dolly Madison, Dolly Madison Creme Boat | Commercial snack cakes and muffins | Cobblers, eclairs, turnovers, other pastries |
| desserts - miscellaneous, snacks - commercial packaged, brand name listing, Dolly Madison, Dolly Madison Devil's Food Flip | Commercial snack cakes and muffins | Cakes |
| desserts - miscellaneous, snacks - commercial packaged, brand name listing, Dolly Madison, Dolly Madison English Cruller | Commercial snack sweet rolls and coffee cakes | Danish, breakfast pastries, doughnuts, granola bars |
| desserts - miscellaneous, snacks - commercial packaged, brand name listing, Dolly Madison, Dolly Madison German Chocolate Cake | Commercial snack cakes and muffins | Cakes |
| desserts - miscellaneous, snacks - commercial packaged, brand name listing, Dolly Madison, Dolly Madison Googles | Commercial snack cakes and muffins | Cakes |
| desserts - miscellaneous, snacks - commercial packaged, brand name listing, Dolly Madison, Dolly Madison Honey Wheat Cinnamon Twirl | Commercial snack sweet rolls and coffee cakes | White breads, rolls |
| desserts - miscellaneous, snacks - commercial packaged, brand name listing, Dolly Madison, Dolly Madison Muffins - Banana Walnut | Commercial snack cakes and muffins | Other muffins, popovers |
| desserts - miscellaneous, snacks - commercial packaged, brand name listing, Dolly Madison, Dolly Madison Pie - Pecan | Commercial snack pies | Pies |
| desserts - miscellaneous, snacks - commercial packaged, brand name listing, Dolly Madison, Dolly Madison Pie - Pineapple | Commercial snack pies | Pies |
| desserts - miscellaneous, snacks - commercial packaged, brand name listing, Dolly Madison, Dolly Madison Raspberry Squares | Commercial snack cakes and muffins | Cakes |
| desserts - miscellaneous, snacks - commercial packaged, brand name listing, Dolly Madison, Dolly Madison Spice Cupcake | Commercial snack cakes and muffins | Cakes |
| desserts - miscellaneous, snacks - commercial packaged, brand name listing, Dolly Madison, Dolly Madison Sweet Potato Flip | Commercial snack cakes and muffins | Cakes |
| desserts - miscellaneous, snacks - commercial packaged, brand name listing, Dolly Madison, Dolly Madison Sweet Roll - Lemon | Commercial snack sweet rolls and coffee cakes | White breads, rolls |
| desserts - miscellaneous, snacks - commercial packaged, brand name listing, Weight Watchers, Weight Watchers Chocolate Raspberry Royale | Commercial snack - miscellaneous desserts | Cakes |
| desserts - miscellaneous, snacks - commercial packaged, brand name listing, Weight Watchers, Weight Watchers French Style Cheesecake | Commercial snack cakes and muffins | Cakes |
| desserts - miscellaneous, snacks - commercial packaged, brand name listing, Weight Watchers, Weight Watchers New York Style Cheesecake | Commercial snack cakes and muffins | Cakes |
| desserts - miscellaneous, snacks - commercial packaged, brand name listing, Weight Watchers, Weight Watchers Strawberry Parfait Royale | Commercial snack - miscellaneous desserts | Milk desserts, frozen |
| desserts - miscellaneous, snacks - commercial packaged, brand name listing, Weight Watchers, Weight Watchers Triple Chocolate Eclair | Commercial snack pies | Cobblers, eclairs, turnovers, other pastries |
| desserts - miscellaneous, snacks - commercial packaged, brand name listing, Dolly Madison, Dolly Madison Pie - Chocolate | Commercial snack pies | Pies |
| desserts - miscellaneous, snacks - commercial packaged, brand name listing, Weight Watchers, Weight Watchers Blueberry Muffin - fat free | Commercial snack cakes and muffins | Other muffins, popovers |
| ice cream and frozen desserts, treats (frozen prepackaged), brand name listing, Baskin-Robbins, Baskin-Robbins Cappy Blast Bar - cappuccino blast | Frozen treats | Milk desserts, frozen |
| ice cream and frozen desserts, treats (frozen prepackaged), brand name listing, Baskin-Robbins, Baskin-Robbins Cappy Blast Bar - mocha cappuccino blast | Frozen treats | Milk desserts, frozen |
| ice cream and frozen desserts, treats (frozen prepackaged), brand name listing, Baskin-Robbins, Baskin-Robbins Chillyburger - chocolate chip | Frozen treats | Milk desserts, frozen |
| ice cream and frozen desserts, treats (frozen prepackaged), brand name listing, Baskin-Robbins, Baskin-Robbins Chillyburger - mint chocolate chip | Frozen treats | Milk desserts, frozen |
| ice cream and frozen desserts, treats (frozen prepackaged), brand name listing, Baskin-Robbins, Baskin-Robbins Sundae Bar - jamocha almond fudge | Frozen treats | Milk desserts, frozen |
| ice cream and frozen desserts, treats (frozen prepackaged), brand name listing, Baskin-Robbins, Baskin-Robbins Sundae Bar - peanut butter chocolate | Frozen treats | Milk desserts, frozen |
| ice cream and frozen desserts, treats (frozen prepackaged), brand name listing, Baskin-Robbins, Baskin-Robbins Sundae Bar - pralines 'n cream | Frozen treats | Milk desserts, frozen |
| ice cream and frozen desserts, treats (frozen prepackaged), brand name listing, Baskin-Robbins, Baskin-Robbins Tiny Toon Bar - chocolate chip | Frozen treats | Milk desserts, frozen |
| ice cream and frozen desserts, treats (frozen prepackaged), brand name listing, Ben & Jerry's, Ben & Jerry's Chunky Monkey Peace Pop | Frozen treats | Milk desserts, frozen |
| ice cream and frozen desserts, treats (frozen prepackaged), brand name listing, Ben & Jerry's, Ben & Jerry's Doonesberry Sorbet Squeeze Up | Frozen treats | Milk desserts, frozen |
| ice cream and frozen desserts, treats (frozen prepackaged), brand name listing, Ben & Jerry's, Ben & Jerry's Strawberry Kiwi Sorbet Squeeze Up | Frozen treats | Milk desserts, frozen |
| ice cream and frozen desserts, treats (frozen prepackaged), brand name listing, Ben & Jerry's, Ben & Jerry's Vanilla Brownie Bar | Frozen treats | Milk desserts, frozen |
| ice cream and frozen desserts, treats (frozen prepackaged), brand name listing, Ben & Jerry's, Ben & Jerry's Vanilla Peace Pop | Frozen treats | Milk desserts, frozen |
| ice cream and frozen desserts, treats (frozen prepackaged), brand name listing, Blue Bell, Blue Bell Almond Bar | Frozen treats | Milk desserts, frozen |
| ice cream and frozen desserts, treats (frozen prepackaged), brand name listing, Blue Bell, Blue Bell Cookies 'N Cream Mini | Frozen treats | Milk desserts, frozen |
| ice cream and frozen desserts, treats (frozen prepackaged), brand name listing, Blue Bell, Blue Bell Country Cone - mini | Frozen treats | Milk desserts, frozen |
| ice cream and frozen desserts, treats (frozen prepackaged), brand name listing, Blue Bell, Blue Bell Country Cone - vanilla | Frozen treats | Milk desserts, frozen |
| ice cream and frozen desserts, treats (frozen prepackaged), brand name listing, Blue Bell, Blue Bell Eskimo Pie | Frozen treats | Milk desserts, frozen |
| ice cream and frozen desserts, treats (frozen prepackaged), brand name listing, Blue Bell, Blue Bell Fudge Bar | Frozen treats | Milk desserts, frozen |
| ice cream and frozen desserts, treats (frozen prepackaged), brand name listing, Blue Bell, Blue Bell Fudge Bar - mini | Frozen treats | Milk desserts, frozen |
| ice cream and frozen desserts, treats (frozen prepackaged), brand name listing, Blue Bell, Blue Bell Fudge Blast | Frozen treats | Milk desserts, frozen |
| ice cream and frozen desserts, treats (frozen prepackaged), brand name listing, Blue Bell, Blue Bell Ice Cream Cup - cookies 'n cream | Ice cream, ice milk, sherbet, nondairy frozen dessert, and milkshakes | Milk desserts, frozen |
| ice cream and frozen desserts, treats (frozen prepackaged), brand name listing, Blue Bell, Blue Bell Ice Cream Stick Slices - chocolate | Frozen treats | Milk desserts, frozen |
| ice cream and frozen desserts, treats (frozen prepackaged), brand name listing, Blue Bell, Blue Bell Ice Cream Stick Slices - vanilla | Frozen treats | Milk desserts, frozen |
| ice cream and frozen desserts, treats (frozen prepackaged), brand name listing, Blue Bell, Blue Bell Krunch Bar | Frozen treats | Milk desserts, frozen |
| ice cream and frozen desserts, treats (frozen prepackaged), brand name listing, Blue Bell, Blue Bell Megabite | Frozen treats | Ices and popsicles |
| ice cream and frozen desserts, treats (frozen prepackaged), brand name listing, Blue Bell, Blue Bell Mini Mooos | Frozen treats | Milk desserts, frozen |
| ice cream and frozen desserts, treats (frozen prepackaged), brand name listing, Blue Bell, Blue Bell Orange Cream Bar | Frozen treats | Milk desserts, frozen |
| ice cream and frozen desserts, treats (frozen prepackaged), brand name listing, Blue Bell, Blue Bell Orange Sherbet Pop Ups | Frozen treats | Milk desserts, frozen |
| ice cream and frozen desserts, treats (frozen prepackaged), brand name listing, Blue Bell, Blue Bell Snow Cone | Frozen treats | Ices and popsicles |
| ice cream and frozen desserts, treats (frozen prepackaged), brand name listing, Blue Bell, Blue Bell Twistik | Frozen treats | Ices and popsicles |
| ice cream and frozen desserts, treats (frozen prepackaged), brand name listing, Borden, Borden Chocolate Coated Toffee Ice Cream Bar | Frozen treats | Milk desserts, frozen |
| ice cream and frozen desserts, treats (frozen prepackaged), brand name listing, Borden, Borden Frosty Pops | Frozen treats | Ices and popsicles |
| ice cream and frozen desserts, treats (frozen prepackaged), brand name listing, Borden, Borden Ice Cream Sandwich - vanilla with vanilla wafers | Frozen treats | Milk desserts, frozen |
| ice cream and frozen desserts, treats (frozen prepackaged), brand name listing, Dole, Dole Fruit Juice Bar no sugar added - all flavors | Frozen treats | Mixtures of fruits and nonfruit items |
| ice cream and frozen desserts, treats (frozen prepackaged), brand name listing, Dole, Dole Fruit 'N Cream Mixed Berry Bar | Frozen treats | Milk desserts, frozen |
| ice cream and frozen desserts, treats (frozen prepackaged), brand name listing, Dove, Dove Bar - green mint & chocolate fudge truffle swirl with dark chocolate | Frozen treats | Milk desserts, frozen |
| ice cream and frozen desserts, treats (frozen prepackaged), brand name listing, Dove, Dove Bar - vanilla with white coating | Frozen treats | Milk desserts, frozen |
| ice cream and frozen desserts, treats (frozen prepackaged), brand name listing, Eskimo Pie, Eskimo Pie - reduced fat | Frozen treats | Milk desserts, frozen |
| ice cream and frozen desserts, treats (frozen prepackaged), brand name listing, Eskimo Pie, Eskimo Pie Pudding Bar - 97% fat free | Frozen treats | Milk desserts, frozen |
| ice cream and frozen desserts, treats (frozen prepackaged), brand name listing, Edy's, Edy's Fruit Bar - lime | Frozen treats | Mixtures of fruits and nonfruit items |
| ice cream and frozen desserts, treats (frozen prepackaged), brand name listing, Edy's, Edy's Fruit Bar - raspberry kiwi | Frozen treats | Mixtures of fruits and nonfruit items |
| ice cream and frozen desserts, treats (frozen prepackaged), brand name listing, Edy's, Edy's Fruit Bar - strawberry | Frozen treats | Mixtures of fruits and nonfruit items |
| ice cream and frozen desserts, treats (frozen prepackaged), brand name listing, Edy's, Edy's Grand Bar - cookies 'n cream | Frozen treats | Milk desserts, frozen |
| ice cream and frozen desserts, treats (frozen prepackaged), brand name listing, Edy's, Edy's Grand Bar - vanilla & almonds | Frozen treats | Milk desserts, frozen |
| ice cream and frozen desserts, treats (frozen prepackaged), brand name listing, Edy's, Edy's Ice Cream Bar - vanilla 'n milk chocolate | Frozen treats | Milk desserts, frozen |
| ice cream and frozen desserts, treats (frozen prepackaged), brand name listing, Edy's, Edy's Sundae Cone - cookies 'n cream | Frozen treats | Milk desserts, frozen |
| ice cream and frozen desserts, treats (frozen prepackaged), brand name listing, Edy's, Edy's Sundae Cone - vanilla fudge | Frozen treats | Milk desserts, frozen |
| ice cream and frozen desserts, treats (frozen prepackaged), brand name listing, Good Humor, Good Humor Fudgsicle | Frozen treats | Milk desserts, frozen |
| ice cream and frozen desserts, treats (frozen prepackaged), brand name listing, Good Humor, Good Humor Fudgsicle - sugar free | Frozen treats | Milk desserts, frozen |
| ice cream and frozen desserts, treats (frozen prepackaged), brand name listing, Good Humor, Good Humor Klondike - reduced fat no sugar added | Frozen treats | Milk desserts, frozen |
| ice cream and frozen desserts, treats (frozen prepackaged), brand name listing, Good Humor, Good Humor Popsicle - sugar free | Frozen treats | Ices and popsicles |
| ice cream and frozen desserts, treats (frozen prepackaged), brand name listing, Good Humor, Good Humor Reese's Peanut Butter Ice Cream Cups | Frozen treats | Milk desserts, frozen |
| ice cream and frozen desserts, treats (frozen prepackaged), brand name listing, Haagen-Dazs, Haagen-Dazs Ice Cream Bar Extraas - caramel cone explosion | Frozen treats | Milk desserts, frozen |
| ice cream and frozen desserts, treats (frozen prepackaged), brand name listing, Haagen-Dazs, Haagen-Dazs Ice Cream Bar Extraas - cookie dough dynamo | Frozen treats | Milk desserts, frozen |
| ice cream and frozen desserts, treats (frozen prepackaged), brand name listing, Haagen-Dazs, Haagen-Dazs Ice Cream Bar Extraas - iced cappuccino | Frozen treats | Milk desserts, frozen |
| ice cream and frozen desserts, treats (frozen prepackaged), brand name listing, Haagen-Dazs, Haagen-Dazs Ice Cream Bar Extraas - triple brownie overload | Frozen treats | Milk desserts, frozen |
| ice cream and frozen desserts, treats (frozen prepackaged), brand name listing, Haagen-Dazs, Haagen-Dazs Sorbet Bar - chocolate | Frozen treats | Milk desserts, frozen |
| ice cream and frozen desserts, treats (frozen prepackaged), brand name listing, Haagen-Dazs, Haagen-Dazs Sorbet Bar - wild berry | Frozen treats | Milk desserts, frozen |
| ice cream and frozen desserts, treats (frozen prepackaged), brand name listing, Haagen-Dazs, Haagen-Dazs Sorbet 'N Yogurt Bar | Frozen treats | Milk desserts, frozen |
| ice cream and frozen desserts, treats (frozen prepackaged), brand name listing, Kemps, Kemps Dad's Root Beer Floats | Frozen treats | Milk desserts, frozen |
| ice cream and frozen desserts, treats (frozen prepackaged), brand name listing, Kemps, Kemps Chocolate Mousse - fat free | Frozen treats | Milk desserts, frozen |
| ice cream and frozen desserts, treats (frozen prepackaged), brand name listing, Kemps, Kemps Fruit 'N Cream - fat free | Frozen treats | Milk desserts, frozen |
| ice cream and frozen desserts, treats (frozen prepackaged), brand name listing, Kemps, Kemps Sherburst on a Stick - fat free | Frozen treats | Milk desserts, frozen |
| ice cream and frozen desserts, treats (frozen prepackaged), brand name listing, Kemps, Kemps Fudge Jr.'s - no sugar added | Frozen treats | Milk desserts, frozen |
| ice cream and frozen desserts, treats (frozen prepackaged), brand name listing, Kemps, Kemps Ice Cream Bar - reduced fat | Frozen treats | Milk desserts, frozen |
| ice cream and frozen desserts, treats (frozen prepackaged), brand name listing, Kemps, Kemps Ice Cream Cup Jr.'s - strawberry | Ice cream, ice milk, sherbet, nondairy frozen dessert, and milkshakes | Milk desserts, frozen |
| ice cream and frozen desserts, treats (frozen prepackaged), brand name listing, Kemps, Kemps Krunch Bar - reduced fat | Frozen treats | Milk desserts, frozen |
| ice cream and frozen desserts, treats (frozen prepackaged), brand name listing, Kemps, Kemps Krunch Jr.'s | Frozen treats | Milk desserts, frozen |
| ice cream and frozen desserts, treats (frozen prepackaged), brand name listing, Kemps, Kemps Chocolate Malt Cups - lowfat | Ice cream, ice milk, sherbet, nondairy frozen dessert, and milkshakes | Milk desserts, frozen |
| ice cream and frozen desserts, treats (frozen prepackaged), brand name listing, Kemps, Kemps Mini Ice Cream Sandwich Jr.'s - lowfat | Frozen treats | Milk desserts, frozen |
| ice cream and frozen desserts, treats (frozen prepackaged), brand name listing, Kemps, Kemps Mini Ice Cream Sandwich | Frozen treats | Milk desserts, frozen |
| ice cream and frozen desserts, treats (frozen prepackaged), brand name listing, Kemps, Kemps Moo Jr.'s | Frozen treats | Milk desserts, frozen |
| ice cream and frozen desserts, treats (frozen prepackaged), brand name listing, Kemps, Kemps Neopolitan Ice Cream Cups | Ice cream, ice milk, sherbet, nondairy frozen dessert, and milkshakes | Milk desserts, frozen |
| ice cream and frozen desserts, treats (frozen prepackaged), brand name listing, Kemps, Kemps 100% Fruit Juice Bars | Frozen treats | Mixtures of fruits and nonfruit items |
| ice cream and frozen desserts, treats (frozen prepackaged), brand name listing, Kemps, Kemps Pops | Frozen treats | Ices and popsicles |
| ice cream and frozen desserts, treats (frozen prepackaged), brand name listing, Kemps, Kemps Pops - sugar free | Frozen treats | Ices and popsicles |
| ice cream and frozen desserts, treats (frozen prepackaged), brand name listing, Kemps, Kemps Twirler Cones | Frozen treats | Milk desserts, frozen |
| ice cream and frozen desserts, treats (frozen prepackaged), brand name listing, Kemps, Kemps Watermelon Sherbet Treats | Frozen treats | Milk desserts, frozen |
| ice cream and frozen desserts, treats (frozen prepackaged), brand name listing, Kemps, Kemps What's Up! Orange Sherbet Treats | Frozen treats | Milk desserts, frozen |
| ice cream and frozen desserts, treats (frozen prepackaged), brand name listing, M & M/Mars, Milky Way Chocolate Malt Milkshake - lowfat | Ice cream, ice milk, sherbet, nondairy frozen dessert, and milkshakes | Milk desserts, frozen |
| ice cream and frozen desserts, treats (frozen prepackaged), brand name listing, M & M/Mars, Milky Way Ice Cream Bars - reduced fat | Frozen treats | Milk desserts, frozen |
| ice cream and frozen desserts, treats (frozen prepackaged), brand name listing, M & M/Mars, Snickers Ice Cream Cone | Frozen treats | Milk desserts, frozen |
| ice cream and frozen desserts, treats (frozen prepackaged), brand name listing, M & M/Mars, Starburst Fruit Juice Bars | Frozen treats | Mixtures of fruits and nonfruit items |
| ice cream and frozen desserts, treats (frozen prepackaged), brand name listing, Nestle, Nestle Cool Creations - Cookies & Cream Sandwich | Frozen treats | Milk desserts, frozen |
| ice cream and frozen desserts, treats (frozen prepackaged), brand name listing, Nestle, Nestle Cool Creations - Ice Pop | Frozen treats | Ices and popsicles |
| ice cream and frozen desserts, treats (frozen prepackaged), brand name listing, Nestle, Nestle Cool Creations - Lion King Cone | Frozen treats | Milk desserts, frozen |
| ice cream and frozen desserts, treats (frozen prepackaged), brand name listing, Nestle, Nestle Crunch Crunch King | Frozen treats | Milk desserts, frozen |
| ice cream and frozen desserts, treats (frozen prepackaged), brand name listing, Nestle, Nestle Crunch Ice Cream Bar - reduced fat | Frozen treats | Milk desserts, frozen |
| ice cream and frozen desserts, treats (frozen prepackaged), brand name listing, Nestle, Nestle Crunch Triple Treats | Frozen treats | Milk desserts, frozen |
| ice cream and frozen desserts, treats (frozen prepackaged), brand name listing, Nestle, Nestle Drumstick - mint | Frozen treats | Milk desserts, frozen |
| ice cream and frozen desserts, treats (frozen prepackaged), brand name listing, Nestle, Nestle Drumstick - vanilla caramel | Frozen treats | Milk desserts, frozen |
| ice cream and frozen desserts, treats (frozen prepackaged), brand name listing, Nestle, Flintstones Push-Up Sherbet Treats | Frozen treats | Milk desserts, frozen |
| ice cream and frozen desserts, treats (frozen prepackaged), brand name listing, Schwan's, Schwan's Ice Cream Cup - strawberry nonfat | Ice cream, ice milk, sherbet, nondairy frozen dessert, and milkshakes | Milk desserts, frozen |
| ice cream and frozen desserts, treats (frozen prepackaged), brand name listing, Schwan's, Schwan's Ice Cream Cup - vanilla nonfat | Ice cream, ice milk, sherbet, nondairy frozen dessert, and milkshakes | Milk desserts, frozen |
| ice cream and frozen desserts, treats (frozen prepackaged), brand name listing, Schwan's, Schwan's Krispie Krunch Bar | Frozen treats | Milk desserts, frozen |
| ice cream and frozen desserts, treats (frozen prepackaged), brand name listing, Snackwell's, SnackWell's Frozen Yogurt Bar - lowfat, all flavors | Frozen treats | Milk desserts, frozen |
| ice cream and frozen desserts, treats (frozen prepackaged), brand name listing, Snackwell's, SnackWell's Ice Cream Sandwich - chocolate lowfat | Frozen treats | Milk desserts, frozen |
| ice cream and frozen desserts, treats (frozen prepackaged), brand name listing, Snackwell's, SnackWell's Ice Cream Sandwich - vanilla lowfat | Frozen treats | Milk desserts, frozen |
| ice cream and frozen desserts, treats (frozen prepackaged), brand name listing, TCBY, TCBY Yog-A-Bar - orange swirl | Frozen treats | Milk desserts, frozen |
| ice cream and frozen desserts, treats (frozen prepackaged), brand name listing, TCBY, TCBY Yog-A-Bar - vanilla with Heath toffee pieces | Frozen treats | Milk desserts, frozen |
| ice cream and frozen desserts, treats (frozen prepackaged), brand name listing, TCBY, TCBY Yog-A-Bar - vanilla with no sugar added | Frozen treats | Milk desserts, frozen |
| ice cream and frozen desserts, treats (frozen prepackaged), brand name listing, TCBY, TCBY Yog-A-Bar - vanilla with toasted almonds | Frozen treats | Milk desserts, frozen |
| ice cream and frozen desserts, treats (frozen prepackaged), brand name listing, Tofutti, Tofutti Cuties - Wildberry | Frozen treats | Soybean derived products (excluding milks) |
| ice cream and frozen desserts, treats (frozen prepackaged), brand name listing, Wells' Blue Bunny, Blue Bunny Cool Tubes Rainbow Sherbet Push-Up | Frozen treats | Milk desserts, frozen |
| ice cream and frozen desserts, treats (frozen prepackaged), brand name listing, Wells' Blue Bunny, Blue Bunny Extremes Premium Homemade Vanilla Ice Cream Bar | Frozen treats | Milk desserts, frozen |
| ice cream and frozen desserts, treats (frozen prepackaged), brand name listing, Wells' Blue Bunny, Blue Bunny Super Premium Homemade Vanilla Ice Cream Bar with Almonds | Frozen treats | Milk desserts, frozen |
| ice cream and frozen desserts, treats (frozen prepackaged), brand name listing, Wells' Blue Bunny, Blue Bunny Frozen Yogurt and Fruit Snacks - lowfat | Frozen treats | Milk desserts, frozen |
| ice cream and frozen desserts, treats (frozen prepackaged), brand name listing, Wells' Blue Bunny, Blue Bunny Fruit Juice Bar | Frozen treats | Mixtures of fruits and nonfruit items |
| ice cream and frozen desserts, treats (frozen prepackaged), brand name listing, Wells' Blue Bunny, Blue Bunny Health Smart Fudge Bar - fat free, no sugar added | Frozen treats | Milk desserts, frozen |
| ice cream and frozen desserts, treats (frozen prepackaged), brand name listing, Wells' Blue Bunny, Blue Bunny Health Smart Mint Fudge & Cappuccino Vanilla Bar - fat free | Frozen treats | Milk desserts, frozen |
| ice cream and frozen desserts, treats (frozen prepackaged), brand name listing, Wells' Blue Bunny, Blue Bunny Health Smart Orange/Raspberry Creme Bar - fat free | Frozen treats | Milk desserts, frozen |
| ice cream and frozen desserts, treats (frozen prepackaged), brand name listing, Wells' Blue Bunny, Blue Bunny Health Smart Strawberry/Tropical Sorbet & Creme Bar - fat free | Frozen treats | Milk desserts, frozen |
| ice cream and frozen desserts, treats (frozen prepackaged), brand name listing, Wells' Blue Bunny, Blue Bunny Ice Cream Cup - strawberry marble | Ice cream, ice milk, sherbet, nondairy frozen dessert, and milkshakes | Milk desserts, frozen |
| ice cream and frozen desserts, treats (frozen prepackaged), brand name listing, Wells' Blue Bunny, Looney Tunes Rainbow Sherbet Push Ups | Frozen treats | Milk desserts, frozen |
| ice cream and frozen desserts, treats (frozen prepackaged), brand name listing, Wells' Blue Bunny, Blue Bunny Sweet Freedom English Toffee Lites - sugar free | Frozen treats | Milk desserts, frozen |
| ice cream and frozen desserts, treats (frozen prepackaged), brand name listing, Welch's, Welch's Light Juice Bar - all flavors | Frozen treats | Mixtures of fruits and nonfruit items |
| ice cream and frozen desserts, treats (frozen prepackaged), brand name listing, Rice Dream, Rice Dream Bar - Chocolate | Frozen treats | Cooked cereals, rice |
| ice cream and frozen desserts, treats (frozen prepackaged), brand name listing, Rice Dream, Rice Dream Bar - Chocolate Nutty Bar | Frozen treats | Cooked cereals, rice |
| ice cream and frozen desserts, treats (frozen prepackaged), brand name listing, Rice Dream, Rice Dream Bar - Strawberry | Frozen treats | Milk desserts, frozen |
| ice cream and frozen desserts, treats (frozen prepackaged), brand name listing, Rice Dream, Rice Dream Bar - Vanilla | Frozen treats | Cooked cereals, rice |
| ice cream and frozen desserts, treats (frozen prepackaged), brand name listing, Rice Dream, Rice Dream Bar - Vanilla Nutty Bar | Frozen treats | Cooked cereals, rice |
| ice cream and frozen desserts, treats (frozen prepackaged), brand name listing, Rice Dream, Rice Dream Pie - Chocolate | Frozen treats | Cooked cereals, rice |
| ice cream and frozen desserts, treats (frozen prepackaged), brand name listing, Rice Dream, Rice Dream Pie - Mint | Frozen treats | Cooked cereals, rice |
| ice cream and frozen desserts, treats (frozen prepackaged), brand name listing, Rice Dream, Rice Dream Pie - Mocha | Frozen treats | Cooked cereals, rice |
| ice cream and frozen desserts, treats (frozen prepackaged), brand name listing, Rice Dream, Rice Dream Pie - Vanilla | Frozen treats | Cooked cereals, rice |
| ice cream and frozen desserts, treats (frozen prepackaged), brand name listing, Tofutti, Tofutti Cuties - Blueberry Wave | Frozen treats | Soybean derived products (excluding milks) |
| ice cream and frozen desserts, treats (frozen prepackaged), brand name listing, Tofutti, Tofutti Cuties - Chocolate Wave | Frozen treats | Soybean derived products (excluding milks) |
| ice cream and frozen desserts, treats (frozen prepackaged), brand name listing, Tofutti, Tofutti Cuties - Coffee Break | Frozen treats | Soybean derived products (excluding milks) |
| ice cream and frozen desserts, treats (frozen prepackaged), brand name listing, Tofutti, Tofutti Cuties - Cookies N Cream | Frozen treats | Soybean derived products (excluding milks) |
| ice cream and frozen desserts, treats (frozen prepackaged), brand name listing, Tofutti, Tofutti Cuties - Jazzy | Frozen treats | Soybean derived products (excluding milks) |
| ice cream and frozen desserts, treats (frozen prepackaged), brand name listing, Tofutti, Tofutti Cuties - Mint Chocolate Chip | Frozen treats | Soybean derived products (excluding milks) |
| ice cream and frozen desserts, treats (frozen prepackaged), brand name listing, Tofutti, Tofutti Cuties - Peanut Butter | Frozen treats | Soybean derived products (excluding milks) |
| ice cream and frozen desserts, treats (frozen prepackaged), brand name listing, Tofutti, Tofutti Cuties - Strawberry Wave | Frozen treats | Soybean derived products (excluding milks) |
| ice cream and frozen desserts, treats (frozen prepackaged), brand name listing, Tofutti, Tofutti Cuties - Totally Vanilla | Frozen treats | Soybean derived products (excluding milks) |
| ice cream and frozen desserts, treats (frozen prepackaged), tofu sandwich | Frozen treats | Soybean derived products (excluding milks) |
| ice cream and frozen desserts, treats (frozen prepackaged), rice dessert (non-dairy), carob coated | Frozen treats | Cooked cereals, rice |
| ice cream and frozen desserts, treats (frozen prepackaged), rice dessert (non-dairy), chocolate coated | Frozen treats | Cooked cereals, rice |
| ice cream and frozen desserts, treats (frozen prepackaged), sherbet push-up or pop | Frozen treats | Milk desserts, frozen |
| ice cream and frozen desserts, treats (frozen prepackaged), fudge type bar (fudgesicle), reduced fat and no sugar added | Frozen treats | Milk desserts, frozen |
| ice cream and frozen desserts, treats (frozen prepackaged), fudge type bar (fudgesicle), low carbohydrate | Frozen treats | Milk desserts, frozen |
| ice cream and frozen desserts, treats (frozen prepackaged), Dreamsicle or Creamsicle, no sugar added | Frozen treats | Milk desserts, frozen |
| ice cream and frozen desserts, treats (frozen prepackaged), Dreamsicle or Creamsicle, sugar free | Frozen treats | Milk desserts, frozen |
| ice cream and frozen desserts, treats (frozen prepackaged), ice cream bar, regular, with crunch | Frozen treats | Milk desserts, frozen |
| ice cream and frozen desserts, treats (frozen prepackaged), ice cream bar, regular, with nuts | Frozen treats | Milk desserts, frozen |
| ice cream and frozen desserts, treats (frozen prepackaged), ice cream bar, reduced fat and no sugar added | Frozen treats | Milk desserts, frozen |
| ice cream and frozen desserts, treats (frozen prepackaged), ice cream bar, low carbohydrate | Frozen treats | Milk desserts, frozen |
| ice cream and frozen desserts, treats (frozen prepackaged), drumstick (sundae cone), reduced fat | Frozen treats | Milk desserts, frozen |
| ice cream and frozen desserts, treats (frozen prepackaged), drumstick (sundae cone), no sugar added | Frozen treats | Milk desserts, frozen |
| ice cream and frozen desserts, treats (frozen prepackaged), drumstick (sundae cone), low carbohydrate | Frozen treats | Milk desserts, frozen |
| ice cream and frozen desserts, treats (frozen prepackaged), ice cream sandwich, reduced fat | Frozen treats | Milk desserts, frozen |
| ice cream and frozen desserts, treats (frozen prepackaged), ice cream sandwich, reduced fat and no sugar added | Frozen treats | Milk desserts, frozen |
| ice cream and frozen desserts, treats (frozen prepackaged), ice cream sandwich, low carbohydrate | Frozen treats | Milk desserts, frozen |
| ice cream and frozen desserts, treats (frozen prepackaged), fudge type bar (fudgesicle), fat free | Frozen treats | Milk desserts, frozen |
| ice cream and frozen desserts, treats (frozen prepackaged), ice cream bar, reduced fat | Frozen treats | Milk desserts, frozen |
| desserts - miscellaneous, snacks - commercial packaged, brand name listing, Tastykake, Tastykake Sensables Cupcake - Cream Filled Chocolate, sugar free | Commercial snack cakes and muffins | Cakes |
| desserts - miscellaneous, snacks - commercial packaged, brand name listing, Tastykake, Tastykake Sensables Cupcake - Cream Filled Koffee Kake, sugar free | Commercial snack cakes and muffins | Cakes |
| desserts - miscellaneous, snacks - commercial packaged, brand name listing, Tastykake, Tastykake Sensables Finger Cakes - all flavors, sugar free | Commercial snack cakes and muffins | Cakes |
| desserts - miscellaneous, snacks - commercial packaged, brand name listing, Weight Watchers, Weight Watchers Caramel Cake | Commercial snack cakes and muffins | Cakes |
| desserts - miscellaneous, snacks - commercial packaged, brand name listing, Weight Watchers, Weight Watchers Carrot Cake | Commercial snack cakes and muffins | Cakes |
| desserts - miscellaneous, snacks - commercial packaged, brand name listing, Weight Watchers, Weight Watchers Chocolate Cake | Commercial snack cakes and muffins | Cakes |
| desserts - miscellaneous, snacks - commercial packaged, brand name listing, Weight Watchers, Weight Watchers Golden Sponge Cake | Commercial snack cakes and muffins | Cakes |
| desserts - miscellaneous, snacks - commercial packaged, brand name listing, Weight Watchers, Weight Watchers Lemon Cake | Commercial snack cakes and muffins | Cakes |
| desserts - miscellaneous, snacks - commercial packaged, brand name listing, Weight Watchers, Weight Watchers Smart Ones Key Lime Pie | Commercial snack pies | Pies |
| desserts - miscellaneous, snacks - commercial packaged, brand name listing, Weight Watchers, Weight Watchers Smart Ones Mint Chocolate Chip Sundae | Commercial snack - miscellaneous desserts | Milk desserts, frozen |
| desserts - miscellaneous, snacks - commercial packaged, brand name listing, Weight Watchers, Weight Watchers Smart Ones Mocha Fudge Sundae | Commercial snack - miscellaneous desserts | Milk desserts, frozen |
| desserts - miscellaneous, snacks - commercial packaged, brand name listing, Weight Watchers, Weight Watchers Smart Ones Peanut Butter Cup Sundae | Commercial snack - miscellaneous desserts | Milk desserts, frozen |
| desserts - miscellaneous, snacks - commercial packaged, brand name listing, Weight Watchers, Weight Watchers Smart Ones Strawberry Shortcake | Commercial snack cakes and muffins | Cakes |
| desserts - miscellaneous, snacks - commercial packaged, brand name listing, Dolly Madison, Dolly Madison Pie - Coconut | Commercial snack pies | Pies |
| desserts - miscellaneous, snacks - commercial packaged, brand name listing, Entenmann's, Entenmann's Single Serve Pie - Apple | Commercial snack pies | Pies |
| desserts - miscellaneous, snacks - commercial packaged, brand name listing, Entenmann's, Entenmann's Single Serve Pie - Apple Snack | Commercial snack pies | Pies |
| desserts - miscellaneous, snacks - commercial packaged, brand name listing, Entenmann's, Entenmann's Single Serve Pie - Cherry | Commercial snack pies | Pies |
| desserts - miscellaneous, snacks - commercial packaged, brand name listing, Entenmann's, Entenmann's Single Serve Pie - Cherry Snack | Commercial snack pies | Pies |
| desserts - miscellaneous, snacks - commercial packaged, brand name listing, Entenmann's, Entenmann's Single Serve Pie - Lemon Snack | Commercial snack pies | Pies |
| desserts - miscellaneous, snacks - commercial packaged, brand name listing, Entenmann's, Entenmann's Single Serve Pie - Peach | Commercial snack pies | Pies |
| desserts - miscellaneous, snacks - commercial packaged, brand name listing, Entenmann's, Entenmann's Single Serve Pie - Pineapple | Commercial snack pies | Pies |
| desserts - miscellaneous, snacks - commercial packaged, brand name listing, Hostess, Hostess Fruit Pie - Blackberry | Commercial snack pies | Pies |
| desserts - miscellaneous, snacks - commercial packaged, brand name listing, Little Debbie, Little Debbie Pie - Apple | Commercial snack pies | Pies |
| desserts - miscellaneous, snacks - commercial packaged, brand name listing, Little Debbie, Little Debbie Pie - Cherry | Commercial snack pies | Pies |
| desserts - miscellaneous, snacks - commercial packaged, brand name listing, Little Debbie, Little Debbie Pie - Lemon | Commercial snack pies | Pies |
| desserts - miscellaneous, snacks - commercial packaged, brand name listing, Tastykake, Tastykake Pie - Banana Cream | Commercial snack pies | Pies |
| desserts - miscellaneous, snacks - commercial packaged, brand name listing, Tastykake, Tastykake Pie - Cheesecake | Commercial snack pies | Pies |
| desserts - miscellaneous, snacks - commercial packaged, brand name listing, Tastykake, Tastykake Pie - Tasty Grahams Chocolate Pudding | Commercial snack pies | Pies |
| desserts - miscellaneous, snacks - commercial packaged, brand name listing, Tastykake, Tastykake Pie - Tasty Grahams Vanilla Pudding | Commercial snack pies | Pies |
| desserts - miscellaneous, snacks - commercial packaged, brand name listing, Hostess, Hostess 100 Calorie Packs Carrot Cakes | Commercial snack cakes and muffins | Cakes |
| desserts - miscellaneous, snacks - commercial packaged, brand name listing, Hostess, Hostess 100 Calorie Packs Chocolate Cupcakes | Commercial snack cakes and muffins | Cakes |
| desserts - miscellaneous, snacks - commercial packaged, brand name listing, Hostess, Hostess 100 Calorie Packs Cinnamon Coffee Cakes | Commercial snack sweet rolls and coffee cakes | Coffee cakes, not yeast |
| desserts - miscellaneous, snacks - commercial packaged, brand name listing, Hostess, Hostess 100 Calorie Packs Golden Cakes | Commercial snack cakes and muffins | Cakes |
| desserts - miscellaneous, snacks - commercial packaged, brand name listing, Hostess, Hostess 100 Calorie Packs Strawberry Cupcakes | Commercial snack cakes and muffins | Cakes |
| desserts - miscellaneous, snacks - commercial packaged, brand name listing, Hostess, Hostess 100 Calorie Packs Twinkie Bites | Commercial snack cakes and muffins | Cakes |
| desserts - miscellaneous, snacks - commercial packaged, brand name listing, Hostess, Hostess Cinnamon Streusel Cakes | Commercial snack sweet rolls and coffee cakes | Coffee cakes, not yeast |
| desserts - miscellaneous, snacks - commercial packaged, brand name listing, Hostess, Hostess Cupcake - Golden | Commercial snack cakes and muffins | Cakes |
| desserts - miscellaneous, snacks - commercial packaged, brand name listing, Hostess, Hostess Pound Cakes | Commercial snack cakes and muffins | Cakes |
| desserts - miscellaneous, snacks - commercial packaged, brand name listing, Hostess, Hostess Twinkies - Banana | Commercial snack cakes and muffins | Cakes |
| desserts - miscellaneous, snacks - commercial packaged, brand name listing, Hostess, Hostess Zingers - Butterscotch | Commercial snack cakes and muffins | Cakes |
| desserts - miscellaneous, snacks - commercial packaged, brand name listing, Hostess, Hostess Zingers - Devil's Food | Commercial snack cakes and muffins | Cakes |
| desserts - miscellaneous, snacks - commercial packaged, brand name listing, Hostess, Hostess Zingers - Raspberry | Commercial snack cakes and muffins | Cakes |
| desserts - miscellaneous, snacks - commercial packaged, brand name listing, Hostess, Hostess Zingers - Iced Vanilla Cake | Commercial snack cakes and muffins | Cakes |
| desserts - miscellaneous, snacks - commercial packaged, brand name listing, Tastykake, Tastykake 100 Calorie Carrot Cake | Commercial snack cakes and muffins | Cakes |
| desserts - miscellaneous, snacks - commercial packaged, brand name listing, Tastykake, Tastykake Bunny Treats | Commercial snack cakes and muffins | Cakes |
| desserts - miscellaneous, snacks - commercial packaged, brand name listing, Tastykake, Tastykake Cupcake - Chocolate Iced | Commercial snack cakes and muffins | Cakes |
| desserts - miscellaneous, snacks - commercial packaged, brand name listing, Tastykake, Tastykake Hippity Hops | Commercial snack cakes and muffins | Cakes |
| desserts - miscellaneous, snacks - commercial packaged, brand name listing, Tastykake, Tastykake Junior - Strawberry Shortcake | Commercial snack cakes and muffins | Cakes |
| desserts - miscellaneous, snacks - commercial packaged, brand name listing, Tastykake, Tastykake Kandy Kake - Peanut Butter Doublicious | Commercial snack cakes and muffins | Cakes |
| desserts - miscellaneous, snacks - commercial packaged, brand name listing, Tastykake, Tastykake Kandy Kake - Raspberry | Commercial snack cakes and muffins | Cakes |
| desserts - miscellaneous, snacks - commercial packaged, brand name listing, Tastykake, Tastykake Krimpet - Kreme Filled Chocolate | Commercial snack cakes and muffins | Cakes |
| desserts - miscellaneous, snacks - commercial packaged, brand name listing, Tastykake, Tastykake Krimpet - Lemon | Commercial snack cakes and muffins | Cakes |
| desserts - miscellaneous, snacks - commercial packaged, brand name listing, Tastykake, Tastykake Krimpet - Pancake | Commercial snack cakes and muffins | Cakes |
| desserts - miscellaneous, snacks - commercial packaged, brand name listing, Tastykake, Tastykake Krimpet - Spice Kake | Commercial snack cakes and muffins | Cakes |
| desserts - miscellaneous, snacks - commercial packaged, brand name listing, Tastykake, Tastykake Snowballs | Commercial snack cakes and muffins | Cakes |
| desserts - miscellaneous, snacks - commercial packaged, brand name listing, Tastykake, Tastykake Sparklecakes | Commercial snack cakes and muffins | Cakes |
| desserts - miscellaneous, snacks - commercial packaged, brand name listing, Tastykake, Tastykake Sweetiekakes | Commercial snack cakes and muffins | Cakes |
| desserts - miscellaneous, snacks - commercial packaged, brand name listing, Little Debbie, Little Debbie Boston Creme Rolls | Commercial snack cakes and muffins | Cakes |
| desserts - miscellaneous, snacks - commercial packaged, brand name listing, Little Debbie, Little Debbie Christmas Tree Cakes - Vanilla Frosted | Commercial snack cakes and muffins | Cakes |
| desserts - miscellaneous, snacks - commercial packaged, brand name listing, Little Debbie, Little Debbie Cupcakes - Orange | Commercial snack cakes and muffins | Cakes |
| desserts - miscellaneous, snacks - commercial packaged, brand name listing, Little Debbie, Little Debbie Cupcakes - Strawberry | Commercial snack cakes and muffins | Cakes |
| desserts - miscellaneous, snacks - commercial packaged, brand name listing, Little Debbie, Little Debbie Double Chocolate Rolls | Commercial snack cakes and muffins | Cakes |
| desserts - miscellaneous, snacks - commercial packaged, brand name listing, Little Debbie, Little Debbie Stars and Stripes Cakes | Commercial snack cakes and muffins | Cakes |
| desserts - miscellaneous, snacks - commercial packaged, brand name listing, Drake's, Drake's Devil Dogs reduced fat | Commercial snack cakes and muffins | Cakes |
| desserts - miscellaneous, snacks - commercial packaged, brand name listing, Entenmann's, Entenmann's Little Bites 100 Calorie Snack Cakes - Carrot Creme | Commercial snack cakes and muffins | Cakes |
| desserts - miscellaneous, snacks - commercial packaged, brand name listing, Entenmann's, Entenmann's Little Bites 100 Calorie Snack Cakes - Golden Creme | Commercial snack cakes and muffins | Cakes |
| desserts - miscellaneous, snacks - commercial packaged, brand name listing, Entenmann's, Entenmann's Single Serve - Crumb Cake | Commercial snack sweet rolls and coffee cakes | Coffee cakes, not yeast |
| desserts - miscellaneous, snacks - commercial packaged, brand name listing, Entenmann's, Entenmann's Single Serve - Crumb Coffee Cake | Commercial snack sweet rolls and coffee cakes | Coffee cakes, not yeast |
| desserts - miscellaneous, snacks - commercial packaged, brand name listing, Entenmann's, Entenmann's Single Serve - Fudge Iced Golden Cake | Commercial snack cakes and muffins | Cakes |
| desserts - miscellaneous, snacks - commercial packaged, brand name listing, Entenmann's, Entenmann's Single Serve - Loaf Cake | Commercial snack cakes and muffins | Cakes |
| desserts - miscellaneous, snacks - commercial packaged, brand name listing, Entenmann's, Entenmann's Single Serve - Marble Loaf Cake | Commercial snack cakes and muffins | Cakes |
| desserts - miscellaneous, snacks - commercial packaged, brand name listing, Moon Pie, Moon Pie - Banana | Commercial snack cakes and muffins | Cookies |
| desserts - miscellaneous, snacks - commercial packaged, brand name listing, Moon Pie, Moon Pie - Banana Double Decker | Commercial snack cakes and muffins | Cookies |
| desserts - miscellaneous, snacks - commercial packaged, brand name listing, Moon Pie, Moon Pie - Banana Mini | Commercial snack cakes and muffins | Cookies |
| desserts - miscellaneous, snacks - commercial packaged, brand name listing, Moon Pie, Moon Pie - Chocolate Double Decker | Commercial snack cakes and muffins | Cookies |
| desserts - miscellaneous, snacks - commercial packaged, brand name listing, Moon Pie, Moon Pie - Chocolate Mini | Commercial snack cakes and muffins | Cookies |
| desserts - miscellaneous, snacks - commercial packaged, brand name listing, Moon Pie, Moon Pie - Lemon Double Decker | Commercial snack cakes and muffins | Cookies |
| desserts - miscellaneous, snacks - commercial packaged, brand name listing, Moon Pie, Moon Pie - Orange Double Decker | Commercial snack cakes and muffins | Cookies |
| desserts - miscellaneous, snacks - commercial packaged, brand name listing, Moon Pie, Moon Pie - Strawberry Double Decker | Commercial snack cakes and muffins | Cookies |
| desserts - miscellaneous, snacks - commercial packaged, brand name listing, Moon Pie, Moon Pie - Vanilla Double Decker | Commercial snack cakes and muffins | Cookies |
| desserts - miscellaneous, snacks - commercial packaged, brand name listing, Moon Pie, Moon Pie - Vanilla Mini | Commercial snack cakes and muffins | Cookies |
| desserts - miscellaneous, snacks - commercial packaged, brand name listing, Tastykake, Tastykake Kandy Kake - Chocolate with Mint | Commercial snack cakes and muffins | Cakes |
| desserts - miscellaneous, snacks - commercial packaged, brand name listing, Entenmann's, Entenmann's Single Serve Enten-Mini's Cupcake - Chocolate Creme | Commercial snack cakes and muffins | Cakes |
| desserts - miscellaneous, snacks - commercial packaged, brand name listing, Entenmann's, Entenmann's Single Serve Enten-Mini's Cupcake - Sponge Creme | Commercial snack cakes and muffins | Cakes |
| desserts - miscellaneous, snacks - commercial packaged, brand name listing, Entenmann's, Entenmann's Single Serve Enten-Mini's Butterscotch Cake | Commercial snack cakes and muffins | Cakes |
| desserts - miscellaneous, snacks - commercial packaged, brand name listing, Entenmann's, Entenmann's Single Serve Enten-Mini's Carrot Cake | Commercial snack cakes and muffins | Cakes |
| desserts - miscellaneous, snacks - commercial packaged, brand name listing, Hostess, Hostess Bear Claw | Commercial snack sweet rolls and coffee cakes | Danish, breakfast pastries, doughnuts |
| desserts - miscellaneous, snacks - commercial packaged, brand name listing, Lance, Lance Honey Bun - Glazed | Commercial snack sweet rolls and coffee cakes | White breads, rolls |
| desserts - miscellaneous, snacks - commercial packaged, brand name listing, Lance, Lance Honey Bun - Iced | Commercial snack sweet rolls and coffee cakes | White breads, rolls |
| desserts - miscellaneous, snacks - commercial packaged, brand name listing, Little Debbie, Little Debbie Iced Honey Buns | Commercial snack sweet rolls and coffee cakes | White breads, rolls |
| desserts - miscellaneous, snacks - commercial packaged, brand name listing, Pillsbury, Pillsbury Grands! Cinnabon Cinnamon Roll with Icing - all flavors | Commercial snack sweet rolls and coffee cakes | White breads, rolls |
| desserts - miscellaneous, snacks - commercial packaged, brand name listing, Pillsbury, Pillsbury Cinnamon Mini-Bites with Icing | Commercial snack sweet rolls and coffee cakes | White breads, rolls |
| desserts - miscellaneous, snacks - commercial packaged, brand name listing, Pillsbury, Pillsbury Cinnamon Roll Reduced Fat with Icing | Commercial snack sweet rolls and coffee cakes | White breads, rolls |
| desserts - miscellaneous, snacks - commercial packaged, brand name listing, Drake's, Drake's Coffee Cake low fat | Commercial snack sweet rolls and coffee cakes | Coffee cakes, not yeast |
| desserts - miscellaneous, snacks - commercial packaged, brand name listing, Weight Watchers, Weight Watchers Smart Ones Raspberry Cheesecake Sundae | Commercial snack - miscellaneous desserts | Milk desserts, frozen |
| desserts - miscellaneous, snacks - commercial packaged, brand name listing, Weight Watchers, Weight Watchers Smart Ones Turtle Sundae | Commercial snack - miscellaneous desserts | Milk desserts, frozen |

# **Supplementary Table S3: Microwave Food**

| **Food Description** | **NCC Database Food Group Description** | **USDA Food Group Description** |
| --- | --- | --- |
| fish and seafood, commercial "pre-coated", brand name listing, Gorton's, Gorton's Crunchy Shrimp - microwave | Shellfish | Shellfish |
| mixed dish, brand name listing, Dinty Moore, Dinty Moore Chicken Stew microwave cup | Commercial entrees and dinners - single item | Frozen or shelf-stable plate meals with meat, poultry, fish as major ingredient |
| mixed dish, brand name listing, Dinty Moore, Dinty Moore Corned Beef Hash microwave cup | Commercial entrees and dinners - single item | Frozen or shelf-stable plate meals with meat, poultry, fish as major ingredient |
| mixed dish, brand name listing, Dinty Moore, Dinty Moore Meatball Stew microwave cup | Commercial entrees and dinners - single item | Meat, poultry, fish with starch item and vegetables |
| snacks, popcorn, brand name listing, microwave popcorn - unknown manufacturer | Popcorn | Salty snacks from grain products |
| snacks, popcorn, microwave popped from package, regular | Popcorn | Salty snacks from grain products |
| snacks, popcorn, microwave popped from package, regular, low sodium | Popcorn | Salty snacks from grain products |
| snacks, popcorn, microwave popped from package, light | Popcorn | Salty snacks from grain products |
| mixed dish, brand name listing, Dinty Moore, Dinty Moore Hearty Burger Stew microwave cup | Commercial entrees and dinners - single item | Frozen or shelf-stable plate meals with meat, poultry, fish as major ingredient |
| mixed dish, brand name listing, Dinty Moore, Dinty Moore Turkey Stew microwave cup | Commercial entrees and dinners - single item | Frozen or shelf-stable plate meals with meat, poultry, fish as major ingredient |
| mixed dish, brand name listing, Dinty Moore, Dinty Moore Corned Beef Hash microwave cup | Commercial entrees and dinners - single item | Frozen or shelf-stable plate meals with meat, poultry, fish as major ingredient |
| snacks, popcorn, microwave popped from package, 94% fat free | Popcorn | Salty snacks from grain products |

**Supplemental Table S4**: Mean (standard deviation) of recoded questionnaire responses by quintile of BPAe (Composite BPA Exposure Score). Higher values indicate more potential exposure to BPA.

|  | **1 (Low Risk)** | **2** | **3** | **4** | **5 (High risk)** | **Total** |
| --- | --- | --- | --- | --- | --- | --- |
|  | n=81 | n=82 | n=80 | n=81 | n=80 | n=404 |
| **In a typical week, how often do you eat canned food?** | 1.7 (±0.8) | 1.8 (±0.7) | 1.7 (±0.6) | 1.8 (±0.6) | 2.0 (±0.9) | 1.8 (±0.7) |
| Missing | 1 (1.2%) | 0 (0%) | 0 (0%) | 0 (0%) | 0 (0%) | 1 (0.2%) |
| **How often do you microwave food stored in plastic containers?** | 1.4 (±0.6) | 2.1 (±0.9) | 2.6 (±0.8) | 2.9 (±0.9) | 3.6 (±0.8) | 2.5 (±1.1) |
| Missing | 4 (4.9%) | 5 (6.1%) | 9 (11.2%) | 4 (4.9%) | 4 (5.0%) | 26 (6.4%) |
| **How often do you drink beverages from a re-usable, hard plastic bottle?** | 3.3 (±1.2) | 3.2 (±1.3) | 3.2 (±1.3) | 3.0 (±1.3) | 2.8 (±1.3) | 3.1 (±1.3) |
| Missing | 0 (0%) | 1 (1.2%) | 0 (0%) | 1 (1.2%) | 0 (0%) | 2 (0.5%) |
| **Do you drink hot beverages from a hard, clear plastic cup?** | 1.8 (±1.0) | 2.1 (±1.0) | 2.7 (±1.1) | 2.5 (±1.1) | 2.6 (±1.0) | 2.3 (±1.1) |
| Missing | 28 (34.6%) | 25 (30.5%) | 15 (18.8%) | 15 (18.5%) | 23 (28.7%) | 106 (26.2%) |
| **How often do you microwave your food with plastic stretch wrap on?** | 1.1 (±0.3) | 1.3 (±0.6) | 1.3 (±0.6) | 1.8 (±0.8) | 2.6 (±1.0) | 1.6 (±0.9) |
| Missing | 25 (30.9%) | 14 (17.1%) | 14 (17.5%) | 11 (13.6%) | 16 (20.0%) | 80 (19.8%) |
| **In a typical day, how many prepared, microwaveable meals do you eat?** | 3.0 (±2.2) | 3.7 (±2.0) | 3.9 (±1.6) | 3.9 (±1.5) | 4.1 (±1.3) | 3.7 (±1.8) |
| Missing | 1 (1.2%) | 0 (0%) | 1 (1.2%) | 1 (1.2%) | 1 (1.2%) | 4 (1.0%) |
| **In a typical day, how many packaged food items do you eat?** | 1.8 (±0.6) | 2.3 (±0.8) | 2.4 (±0.9) | 3.0 (±1.1) | 3.4 (±1.4) | 2.6 (±1.1) |
| Missing | 0 (0%) | 0 (0%) | 2 (2.5%) | 1 (1.2%) | 0 (0%) | 3 (0.7%) |

Responses to questions recoded so that higher values indicate more exposure. For example, 1=never, 2=occasionally, 3=sometimes, 4=often, 5=always.
